# Prediction model for cognitive impairment in maintenance hemodialysis patients: the role of diabetes

**DOI:** 10.3389/fendo.2025.1594605

**Published:** 2025-11-26

**Authors:** Jia Xu, Hao Li, Hualin Qi, Xinhui Zhao

**Affiliations:** 1 Department of Nephrology, The People’s Hospital of Pudong New District in Shanghai, Shanghai, China; 2 Department of Internal Medicine, The People’s Hospital of Pudong New District in Shanghai, Shanghai, China

**Keywords:** cognitive impairment, hemodialysis, diabetes, handgrip strength, TUGT, gait speed

## Abstract

**Objective:**

The aim of this study was to identify risk factors for cognitive impairment (CI) in maintenance hemodialysis (MHD) patients and to develop a predictive model.

**Methods:**

A total of 151 MHD patients from our hospital were recruited between July 2020 and April 2021. Sociodemographic, clinical, and laboratory data were collected. Cognitive function was assessed using the Mini-Mental State Examination (MMSE), whereas physical performance was evaluated using handgrip strength, the Timed Up and Go Test (TUGT), and 4-m gait speed. Univariate and multivariate logistic regression analyses identified risk factors, which were used to develop original and simplified predictive models.

**Results:**

CI was present in 43 patients (28.5%). The simplified model demonstrated discriminatory ability comparable to that of the original model (AUC: 0.737; 95% CI: 0.648–0.818) and was easier to use. A robust nomogram was developed on the basis of the simplified model. Decision curve analysis (DCA) confirmed the clinical utility of both models. Diabetes was identified as an independent risk factor, whereas dialysis duration was not associated with CI.

**Conclusions:**

This study provides a simple predictive model for CI in MHD patients, which could aid in clinical decision-making.

## Introduction

Chronic kidney disease (CKD), with a global prevalence of 14.3%, has become a serious public health problem worldwide (1). End-stage kidney disease (ESKD) is the final stage of CKD, in which renal replacement therapy, such as kidney transplantation, hemodialysis (HD), and peritoneal dialysis, is needed. According to the Chinese Research Data Services Platform (https://www.cnrds.com), 916,647 patients with ESKD were undergoing hemodialysis by the end of 2023. Cognitive impairment (CI) is commonly observed in patients receiving hemodialysis, whose prevalence has been reported to range between 30% and 80% (2, 3). CI has been linked to several negative outcomes, including a lower quality of life, poor medication adherence, and impaired decision-making capacity. Additionally, it is an independent predictor of all-cause premature mortality in individuals receiving maintenance hemodialysis (MHD) (4–6). Therefore, early identification of CI is crucial for timely treatment and delaying the progression of CI in MHD patients.

Cognitive decline is frequently affected by various factors, including age, educational background, cerebrovascular conditions, and a range of metabolic abnormalities (7, 8). Poor physical performance also accompanies CI in HD patients (9). Engaging in multicomponent physical activities, such as balance training, aerobic exercises and muscle strengthening, has been demonstrated to enhance cognition and reduce the risk of dementia (10). This study was conducted to identify factors associated with CI and to develop a predictive model for CI among MHD patients.

## Materials and methods

### Study design and patient population

**Figure 1:**
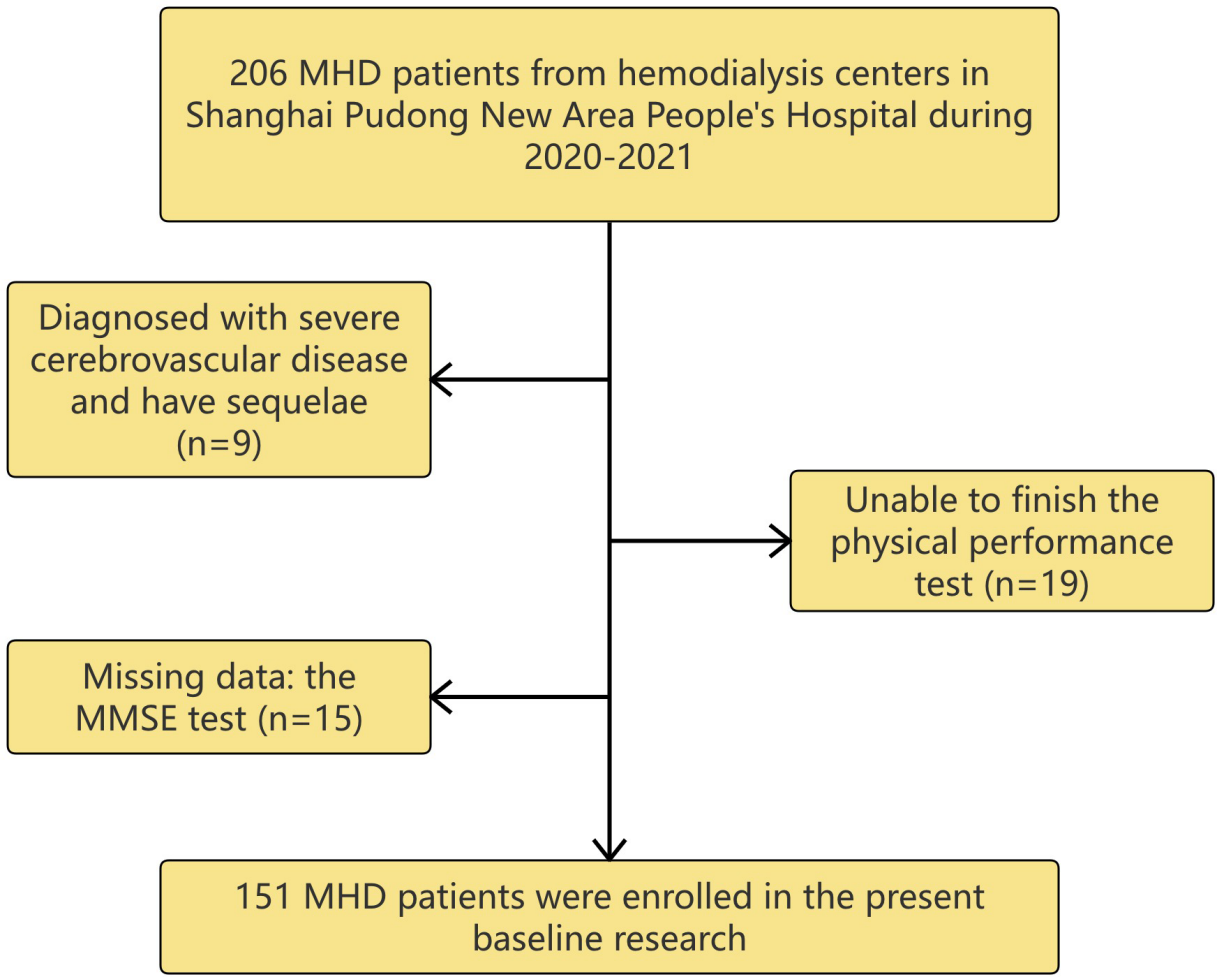
The flowchart of enrollment in this study. A total of 206 participants were collected and 151 patients that underwent maintenance hemodialysis (MHD) were enrolled in this study.

This cross-sectional study enrolled patients who underwent MHD at the blood purification center in Shanghai Pudong New Area People’s Hospital between July 2020 and April 2021. Patients aged 18 years or older who were receiving hemodialysis for 4 hours per session, three times a week, and for more than 3 months were included in the study. Participants with the following conditions were excluded: (1) were diagnosed with severe cerebrovascular disease and had sequelae; (2) were unable to finish the physical performance test; and (3) did not complete the MMSE test (**Figure 1**). All the investigations were conducted in accordance with the Declaration of Helsinki, and the study was approved by the Ethics Committee of Shanghai Pudong New Area People’s Hospital (PRYLW2018-01). All of the participants in this study provided written informed consent. All the details and privacy of the patients have been deidentified.

### Baseline variables

All participants were required to complete a detailed questionnaire on social demographics and clinical characteristics. The demographic characteristics included age, sex, education level, and health behaviors, such as sleep duration and smoking and drinking habits. Clinical characteristics included height, weight, dialysis duration, ultrafiltration volume and concurrent ailments. Their biochemical laboratory indices, including levels of hemoglobin (Hb) and C-reactive protein (CRP), vitamin D, parathyrin, ferritin, trioxypurine, urea, creatinine, alanine aminotransferase (ALT), aspartate aminotransferase (AST), total protein, albumin, cholesterol, triglycerides, high density lipoprotein (HDL), and low density lipoprotein (LDL), were measured before hemodialysis within 1 week of cognitive function assessment. Dialysis adequacy was defined as the total fractional clearance index for urea (Kt/V).

### Assessment of cognitive impairment

The Mini Mental State Examination (MMSE) was used to assess cognitive function. It includes a broad set of cognitive domains that measure the following: time orientation (5 points), place orientation (5 points), registration (3 points), attention and calculation (5 points), recall (3 points) and language (9 points) (11). The possible scores ranged from 0 to 30, with higher scores indicating better cognitive status. The cut-off points used for CI were as follows: 17 for illiterate people, 20 for people with only primary school education, and 24 for people with middle school or higher levels of education (12).

### Assessment of physical performance

The assessment of physical performance consisted of handgrip strength (HS), the Timed Up and Go Test (TUGT) and 4-m gait speed (GS). HS was assessed on their nonfistula hand or dominant hand with an indwelling dialysis catheter using a dynamometer (GRIP-D; Takei, Niigata, Japan). Participants were asked to exert maximum effort twice, and the better of the two measurements was recorded (13). The TUGT counts the number of seconds needed for an individual to stand up from a chair, walk 3 m, turn around, walk back to the chair and sit down again with their back against the chair (14). To determine GS, the participants were asked to walk 4 m twice at their usual pace on a flat surface. GS was calculated as the time taken (s) to complete the 4-m distance (m/s), and the mean speed from the two tests was used in the study (13). Walking aids were allowed during the TUGT and GS test. These performance-based tests were performed before the hemodialysis session.

### Statistical analyses

Data management and statistical analyses were performed in R v4.3.1 (https://www.r-project.org/). The normality of the continuous variables was tested with the Shapiro–Wilk test. Normally distributed data are described as the means ± standard deviations (SDs) and were analyzed by t tests; nonnormally distributed data are presented as medians and interquartile ranges (IQRs) and were analyzed by the Mann–Whitney test. Categorical variables are expressed as percentages and were analyzed through the chi-square test.

Logistic regression was conducted with the R software stats package, and the factors whose p value was < 0.1 were considered related to the CI and then analyzed by multivariate logistic regression. The Hosmer–Lemeshow test, which is a statistical test for goodness of fit for logistic regression models, was conducted with the R software ResourceSelection package. The forest plots were drawn by the R software ggstats package. The nomogram plot and calibration plot were drawn by the R software rms package and ggplot2 package. The ROC analyses were performed by the R software pROC package (15). The 95% confidence interval (CI) for the area under curve(AUC) was calculated using the bootstrap method, a commonly used algorithm in the field of machine learning. The cut-off values for the ROC curve were selected using the Youden index, which maximizes the sum of sensitivity and specificity. The DCA curve was drawn by the R software rmda package. To model the nonlinear relationships between variables, restricted cubic spline regression was employed with the R software rms package. This method offers the advantage of flexibly capturing complex nonlinear associations while maintaining smoothness, without imposing strict assumptions on the functional form. P values < 0.05 were considered to indicate statistical significance.

## Results

### Baseline characteristics of the included patients

A total of 151 patients who underwent MHD at the blood purification center were included in this study. The baseline clinical, biochemical and physical characteristics are shown in **Table 1–1** and **Table 1-2**. The median age of the study patients was 62.0 years (IQR: 53.0-69.0 years), and males accounted for 53.0%. Among MHD patients, 43 (28.5%) had CI. The median dialysis duration was 54.0 months (IQR: 25.5–99.5 months).

**Table 1-1:** Basic characteristics of MHD patients (demographics variables).

Characteristics	Total (N = 151)	Cognitive impairment		P value
		No (N = 108)	Yes (N = 43)	
age	62.0 [53.0;69.0]	59.5 [51.0;67.5]	64.0 [61.0;70.5]	0.006
gender				0.122
male	80 (53.0%)	62 (57.4%)	18 (41.9%)	
female	71 (47.0%)	46 (42.6%)	25 (58.1%)	
BMI	24.3 ± 3.3	24.3 ± 3.4	24.1 ± 3.2	0.667
education				0.112
≤6 years	45 (29.8%)	29 (26.9%)	16 (37.2%)	
6–12 years	92 (60.9%)	66 (61.1%)	26 (60.5%)	
>12 years	14 (9.3%)	13 (12.0%)	1 (2.3%)	
HS	23.4 [18.4;29.2]	25.0 [19.1;30.6]	21.0 [15.9;28.2]	0.009
TUGT	7.4 [6.5; 9.1]	7.1 [6.3; 8.7]	8.0 [6.8;11.5]	0.022
GS	1.1 [0.9; 1.3]	1.1 [1.0; 1.3]	1.0 [0.7; 1.2]	0.006
dialysis duration	54.0 [25.5;99.5]	55.0 [26.0;105.0]	46.0 [24.5;83.5]	0.305
sleep time	6.0 [5.0; 8.0]	6.5 [5.0; 8.0]	6.0 [4.0; 8.0]	0.463
diabetes				0.003
no	117 (77.5%)	91 (84.3%)	26 (60.5%)	
yes	34 (22.5%)	17 (15.7%)	17 (39.5%)	
hypertension				0.224
no	14 (9.3%)	8 (7.4%)	6 (14.0%)	
yes	137 (90.7%)	100 (92.6%)	37 (86.0%)	
coronary heart disease				0.53
no	92 (60.9%)	68 (63.0%)	24 (55.8%)	
yes	59 (39.1%)	40 (37.0%)	19 (44.2%)	
stroke				0.136
no	131 (86.8%)	97 (89.8%)	34 (79.1%)	
yes	20 (13.2%)	11 (10.2%)	9 (20.9%)	
congestive heart failure				0.347
no	105 (69.5%)	78 (72.2%)	27 (62.8%)	
yes	46 (30.5%)	30 (27.8%)	16 (37.2%)	
tobacco				0.207
no	122 (80.8%)	84 (77.8%)	38 (88.4%)	
yes	29 (19.2%)	24 (22.2%)	5 (11.6%)	
alcohol				0.407
no	144 (95.4%)	104 (96.3%)	40 (93.0%)	
yes	7 (4.6%)	4 (3.7%)	3 (7.0%)	

BMI, body mass index; HS, handgrip strength; TUGT, Timed Up and Go Test; GS, 4-m gait speed; P: Values calculated by t-tests, Mann-Whitney test or chi-square test.

**Table 1-2:** Basic characteristics of MHD patients (laboratory variables).

Characteristics	Total (N = 151)	Cognitive impairment		P value
		No (N = 108)	Yes (N = 43)	
Hb	105.0 [93.0;116.0]	106.5 [92.5;119.5]	103.0 [95.0;112.5]	0.507
CRP	1.7 [0.6; 3.6]	1.8 [0.5; 3.5]	1.4 [0.6; 4.0]	0.836
vitamin D	18.9 [14.2;25.0]	19.2 [14.4;25.1]	18.5 [14.1;23.4]	0.698
parathyrin	182.4 [94.8;410.6]	183.2 [96.2;461.1]	180.1 [88.1;300.9]	0.704
ferritin	206.0 [191.0;596.5]	201.5 [191.0;590.5]	218.0 [191.0;670.5]	0.497
Kt/V	1.4 [1.3; 1.6]	1.4 [1.3; 1.6]	1.5 [1.3; 1.7]	0.226
percent of ultrafiltration	5.6 [5.0; 6.8]	5.6 [5.0; 6.9]	5.5 [5.0; 6.7]	0.399
trioxypurine	0.5 [0.4; 0.6]	0.5 [0.4; 0.6]	0.5 [0.3; 0.5]	0.113
urea	28.2 ± 6.9	28.4 ± 7.0	27.8 ± 6.8	0.632
creatinine	1047.0 [891.0;1243.5]	1066.0 [898.0;1252.0]	1027.0 [859.0;1144.0]	0.363
ALT	9.0 [6.0;13.0]	9.0 [7.0;13.0]	8.0 [6.0;12.0]	0.218
AST	11.0 [8.0;14.0]	11.0 [8.0;14.0]	11.0 [8.0;14.0]	0.611
total protein	70.4 [67.8;73.8]	70.7 [67.9;74.0]	70.3 [67.8;72.6]	0.513
albumin	38.5 [37.0;40.8]	38.6 [37.2;40.7]	38.4 [35.9;41.4]	0.598
cholesterol	3.8 [3.2; 4.4]	3.8 [3.3; 4.5]	3.7 [3.1; 4.3]	0.399
triglyceride	2.0 [1.4; 3.3]	1.9 [1.5; 3.3]	2.0 [1.4; 3.1]	0.83
HDL	0.9 [0.8; 1.1]	0.9 [0.8; 1.1]	0.9 [0.7; 1.2]	0.97
LDL	2.1 [1.6; 2.7]	2.2 [1.6; 2.8]	2.1 [1.6; 2.7]	0.763

CRP, C-reactive protein; Kt/V, an indicator for evaluating dialysis adequacy; ALT, aminotransferase; AST, aspartate aminotransferase; HDL, high density lipoprotein; LDL, low density lipoprotein; P, Values calculated by t-tests, Mann-Whitney test or chi-square test.

Among the patients included in this study, some characteristics differed between those with and without CI. Age significantly differed between the two groups (P = 0.006). The median age in the CI group was 64.0 years (IQR: 61.0–70.5), which was greater than the median age of 59.5 years (IQR: 51.0–67.5 years) in the group without CI. These findings suggest that older age may be associated with an increased risk of CI. Additionally, there was a significant difference in the incidence of diabetes between the two groups (P = 0.003). A greater percentage of patients in the CI group had diabetes (39.5%) than in the group without CI (15.7%). These findings suggest that diabetes may influence cognitive function, although the exact relationship remains unclear.

In addition, physical tests revealed significant differences between the two groups of patients: hand grip strength (P = 0.009), TUGT (P = 0.022) and gait speed (P = 0.006). The median hand grip strength in the group without cognitive impairment was 25.0 kg (IQR: 19.1–30.6 kg), whereas it was 21.0 kg (IQR: 15.9–28.2 kg) in the group with cognitive impairment. The TUGT of patients with CI was 8.0 seconds (IQR: 6.8–11.5 s), which was longer than the 7.1 seconds (IQR: 6.3–8.7 s) recorded in patients without CI. Similarly, the gait speed in the group without CI was 1.1 m/s (IQR: 1.0–1.3 m/s), whereas it was 1.0 m/s (IQR: 0.7–1.2 m/s) in the CI group. Poor physical performance may be associated with poor cognitive function, indicating a potential relationship between physical function and cognitive health.

Furthermore, we compared the sex distribution; 57.4% of the participants without CI were male, whereas 41.9% of the participants with CI were male. Conversely, a greater percentage of females were in the CI group (58.1%) than in the group without CI (42.6%). However, this sex difference was not statistically significant (P = 0.122).

### Univariate logistic analysis and multivariate analysis

To identify variables that may be related to CI, we first used univariate logistic analysis for calculation and screening. The outcomes are shown in **Table 2**. Univariate logistic regression analysis revealed several significant associations between baseline characteristics and cognitive impairment. Age was significantly associated with CI, with an odds ratio (OR) of 1.047 (95% CI: 1.012–1.086; P = 0.01). Compared with males, females had a significantly greater likelihood of having a CI (OR: 1.872; 95% CI: 0.92–3.874; P = 0.086), although this association did not reach statistical significance. Education level showed a nonsignificant trend, with patients with more than 12 years of education being less likely to have cognitive impairment (OR: 0.139; 95% CI: 0.007–0.801; P = 0.069). Physical performance was also directly associated with CI: HS was inversely associated, with an OR of 0.936 (95% CI: 0.889–0.982; P = 0.009); the TUGT was directly associated, with an OR of 1.11 (95% CI: 1.018–1.216; P = 0.019); and GS was directly associated, with an OR of 0.134 (95% CI: 0.034–0.483; P = 0.003). Diabetes was also significantly associated with CI, with an OR of 3.5 (95% CI: 1.572–7.872; P = 0.002). Stroke was also significantly related to CI, with an odds ratio (OR) of 2.334 (95% CI: 0.872–6.132; P = 0.085).

**Table 2:** Related factors for patients with CI.

		Univariate analysis		Multivariate analysis	
		OR (95% CI)	P^$^ value	OR (95% CI)	P^ value
age	cont. var.	1.047 (1.012,1.086)	0.01	1.028 (0.983,1.079)	0.239
gender	ref: male				
	female	1.872 (0.92,3.874)	0.086	2.357 (0.863,6.823)	0.101
BMI	cont. var.	0.977 (0.876,1.086)	0.664		
education	ref: ≤6 years				
	6–12 years	0.714 (0.335,1.543)	0.385	1.583 (0.615,4.345)	0.354
	>12 years	0.139 (0.007,0.801)	0.069	0.419 (0.017,3.671)	0.491
HS	cont. var.	0.936 (0.889,0.982)	0.009	0.984 (0.910,1.064)	0.691
TUGT	cont. var.	1.11 (1.018,1.216)	0.019	0.937 (0.804,1.086)	0.391
GS	cont. var.	0.134 (0.034,0.483)	0.003	0.167 (0.016,1.496)	0.117
dialysis duration	cont. var.	0.996 (0.989,1.003)	0.268		
sleep time	cont. var.	0.948 (0.798,1.122)	0.537		
diabetes	ref: no				
	yes	3.5 (1.572,7.872)	0.002	3.013 (1.155,8.062)	0.025
hypertension	ref: no				
	yes	0.493 (0.161,1.587)	0.218		
coronary heart disease	ref: no				
	yes	1.346 (0.653,2.758)	0.417		
stroke	ref: no				
	yes	2.334 (0.872,6.132)	0.085	1.335 (0.428,4.013)	0.609
congestive heart failure	ref: no				
	yes	1.541 (0.721,3.244)	0.257		
tobacco	ref: no				
	yes	0.46 (0.146,1.212)	0.143		
alcohol	ref: no				
	yes	1.95 (0.371,9.223)	0.396		
HB	cont. var.	0.995 (0.975,1.015)	0.604		
CRP	cont. var.	1.01 (0.985,1.036)	0.404		
vitamin D	cont. var.	0.992 (0.949,1.035)	0.725		
parathyrin	cont. var.	1 (0.999,1.001)	0.556		
ferritin	cont. var.	1 (1,1.001)	0.331		
Kt/V	cont. var.	2.651 (0.833,8.779)	0.101		
percent of ultrafiltration	cont. var.	0.908 (0.715,1.143)	0.415		
trioxypurine	cont. var.	0.208 (0.026,1.688)	0.138		
urea	cont. var.	0.988 (0.938,1.04)	0.63		
creatinine	cont. var.	1 (0.999,1.001)	0.862		
ALT	cont. var.	1.005 (0.965,1.041)	0.803		
AST	cont. var.	1.042 (0.991,1.102)	0.115		
total_protein	cont. var.	0.97 (0.905,1.04)	0.39		
albumin	cont. var.	1.012 (0.996,NA)	0.36		
cholesterol	cont. var.	0.875 (0.616,1.118)	0.397		
triglyceride	cont. var.	0.972 (0.802,1.14)	0.746		
HDL	cont. var.	1.497 (0.453,4.811)	0.5		
LDL	cont. var.	0.983 (0.638,1.497)	0.937		

HB, hemoglobin; BMI, body mass index; CRP, C-reactive protein; Kt/V, an indicator for evaluating dialysis adequacy; HS, handgrip strength; TUGT, Timed Up and Go Test;GS, 4-m gait speed; ALT, aminotransferase; AST, aspartate aminotransferase; HDL, high density lipoprotein; LDL, low density lipoprotein; P$, Values calculated by univariate logistics regression analysis; P^, Values calculated by multivariate logistics regression analysis. Red values: P$, Value which is <0.1; P^, Value which is <0.05.

For other variables, such as body mass index (BMI), dialysis duration, hypertension, coronary heart disease, congestive heart failure, tobacco use, alcohol consumption, Hb, CRP, vitamin D, parathyrin, ferritin, Kt/V, urea, creatinine, liver enzymes (ALT, AST), total protein, albumin, cholesterol, triglycerides, HDL, and LDL, no statistically significant differences were detected in the univariate analysis.

Afterwards, 8 variables, namely, age, sex, stroke status, HS, TUGT results, GS, diabetes status, and education level, were chosen to develop a multivariate logistic regression model (Model 1).

**Figure 2:**
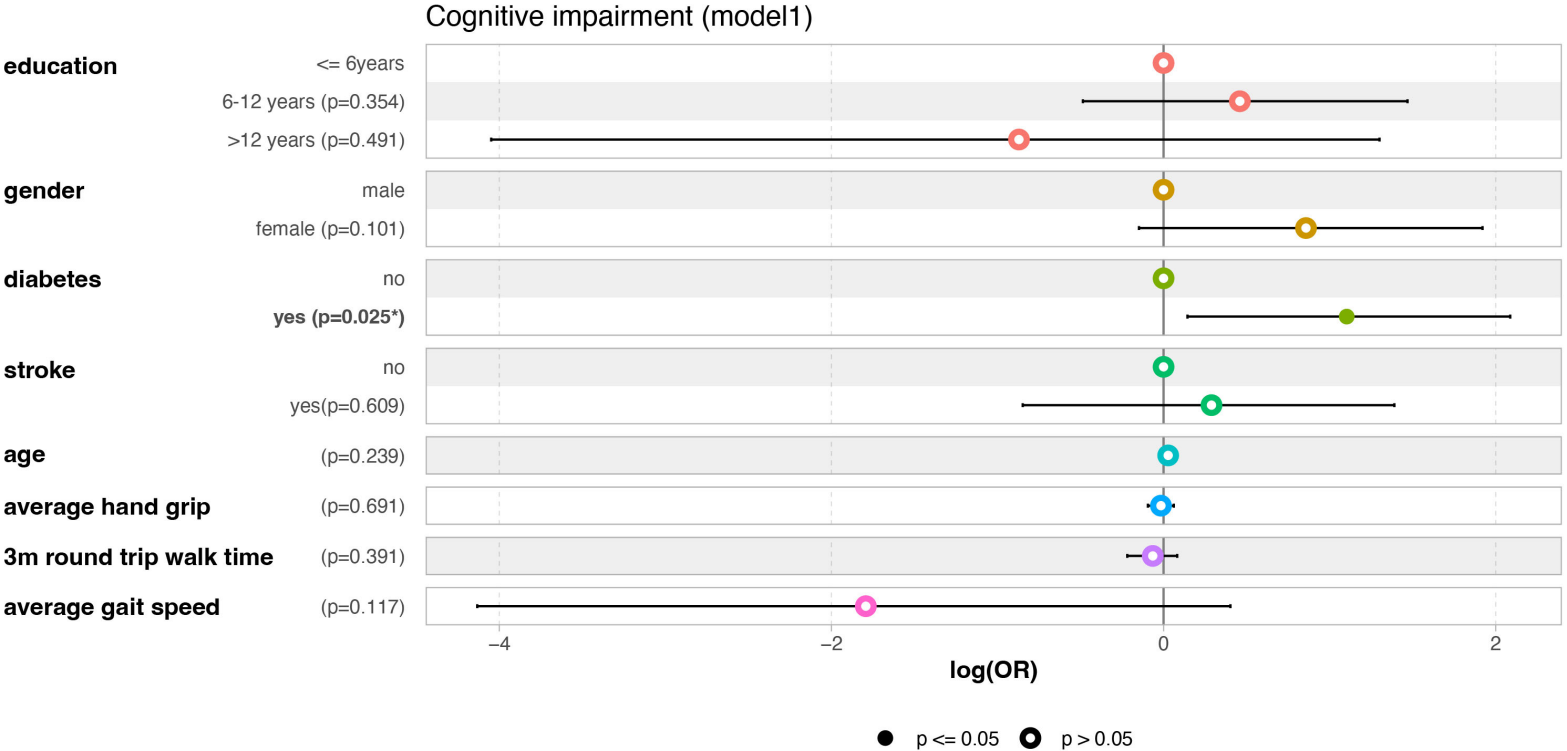
The forest plot of model 1, including 8 variables.

First, the VIF values of these eight variables were calculated to evaluate multicollinearity; the VIF values of these eight variables were all < 2.7. This absence of multicollinearity increases the robustness of the predictive capabilities of the model, underscoring its reliability in capturing the complexities of the underlying relationships among the variables. This model provides important insights into the factors associated with CI in the MHD patient population (**Figure 2**). Among these factors, it is particularly important to pay attention to diabetes, which remains a significant predictor even after adjusting for other variables, with an OR of 3.013 (95% CI: 1.155–8.062; P = 0.025), indicating that diabetes is an independent risk factor for CI. The Hosmer–Lemeshow (H-L) test indicated good predictive performance of the model (P = 0.497).

### A simplified multivariate model and comparisons

**Figure 3:**
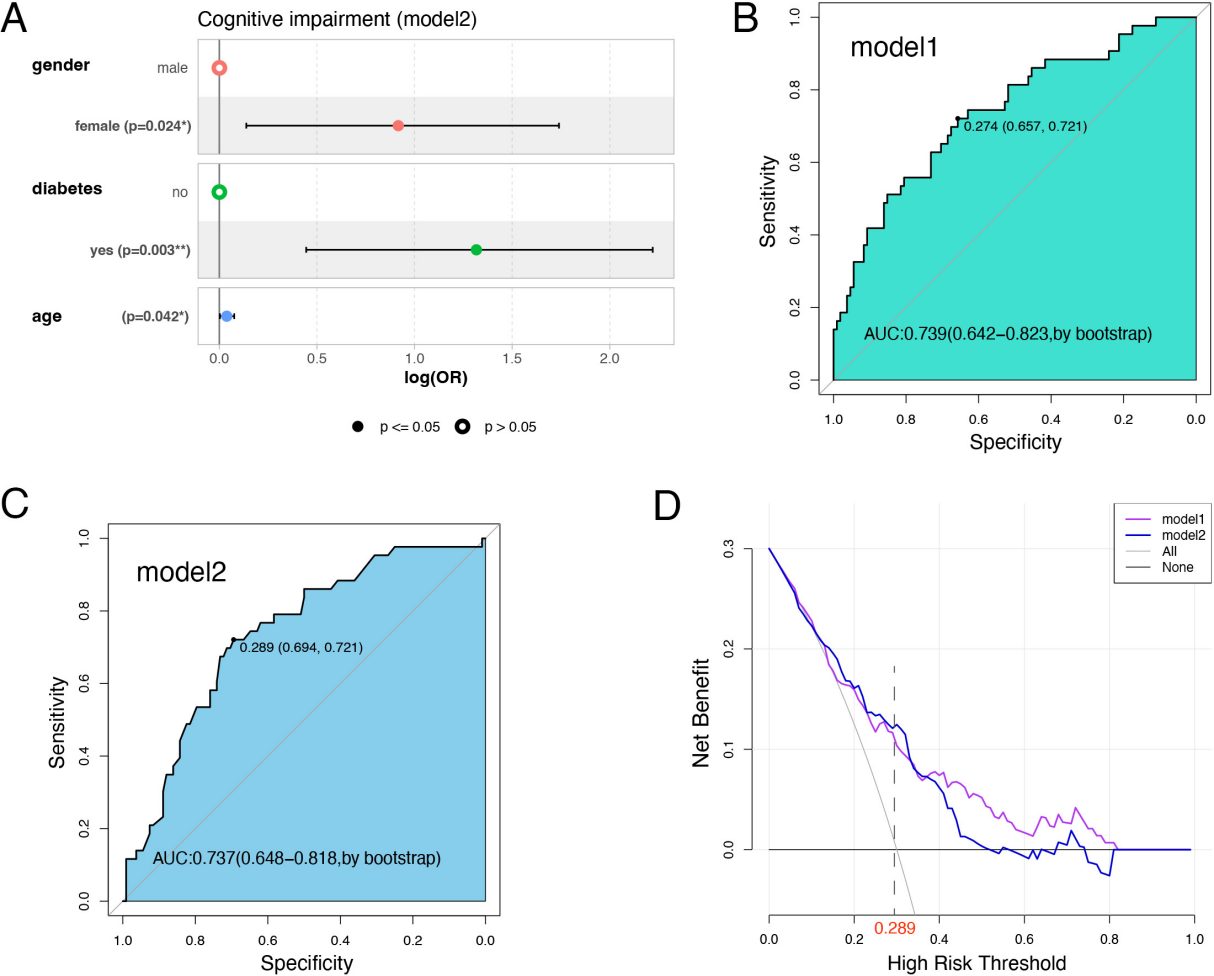
The forest plot of model 2 and comparisons between model1 and model2. **(A)** The forest plot of model1, including 3 variables. **(B)** The ROC curve of model1 and cut-off point. **(C)** The ROC curve of model2 and cut-off point. **(D)** The DCA curve of model1 and model2.

In practical applications, simpler models are often more easily accepted. Therefore, to simplify the model while maintaining good predictive performance, in this study, stepwise regression was applied on the basis of AIC values to reduce the complexity of Model 1 using multivariate logistic regression. The final simplified model (Model 2) consisted of four variables: age, sex, diabetes status, and GS. However, the H-L test did not show a good fit for this model. Despite this, when age, sex, and diabetes status were retained, the model still demonstrated good predictive performance, with no substantial increase in the AIC value, indicating that the model complexity was well controlled (**Figure 3A**). The VIF values of these 3 variables are all < 1.2, suggesting little multicollinearity. Furthermore, a chi-square test comparing the two models revealed no significant difference between them (P = 0.523).

As demonstrated by the ROC curve of Model 1, the AUC was 0.739 (95% CI: 0.642–0.823, by bootstrap), with an optimal cut-off of 0.274 (**Figure 3B**). For Model 2, the AUC was 0.737 (95% CI: 0.648–0.818, by bootstrap), with an optimal cut-off of 0.289 (**Figure 3C**). The ROC curves of both models were similar, suggesting comparable predictive performance. Additionally, a formal comparison of the ROC curves indicated no significant difference between the two models (P = 0.916). This further confirms that the simplified model achieves a predictive performance very similar to that of the original, more complex model.

Moreover, according to the DCA curve, the performance of the two models is remarkably similar. At the cut-off point (0.289), the benefits of both models are comparable, suggesting that Model 2 could serve as a viable substitute for Model 1 (**Figure 3D**). These observations underscore that these two models not only have similar performance in mathematical calculations but also have comparable practical utility.

Notably, on the basis of our literature review, a model developed by Chen et al. in a similar study (PMID: 37828422) can be deployed in this study; however, it demonstrated an AUC of 0.575 and a specificity of 0.259, with a sensitivity of 0.930. This indicates superior predictive performance for Model 1 and Model 2, which were developed in this study (16).

### Development of the nomogram

**Figure 4:**
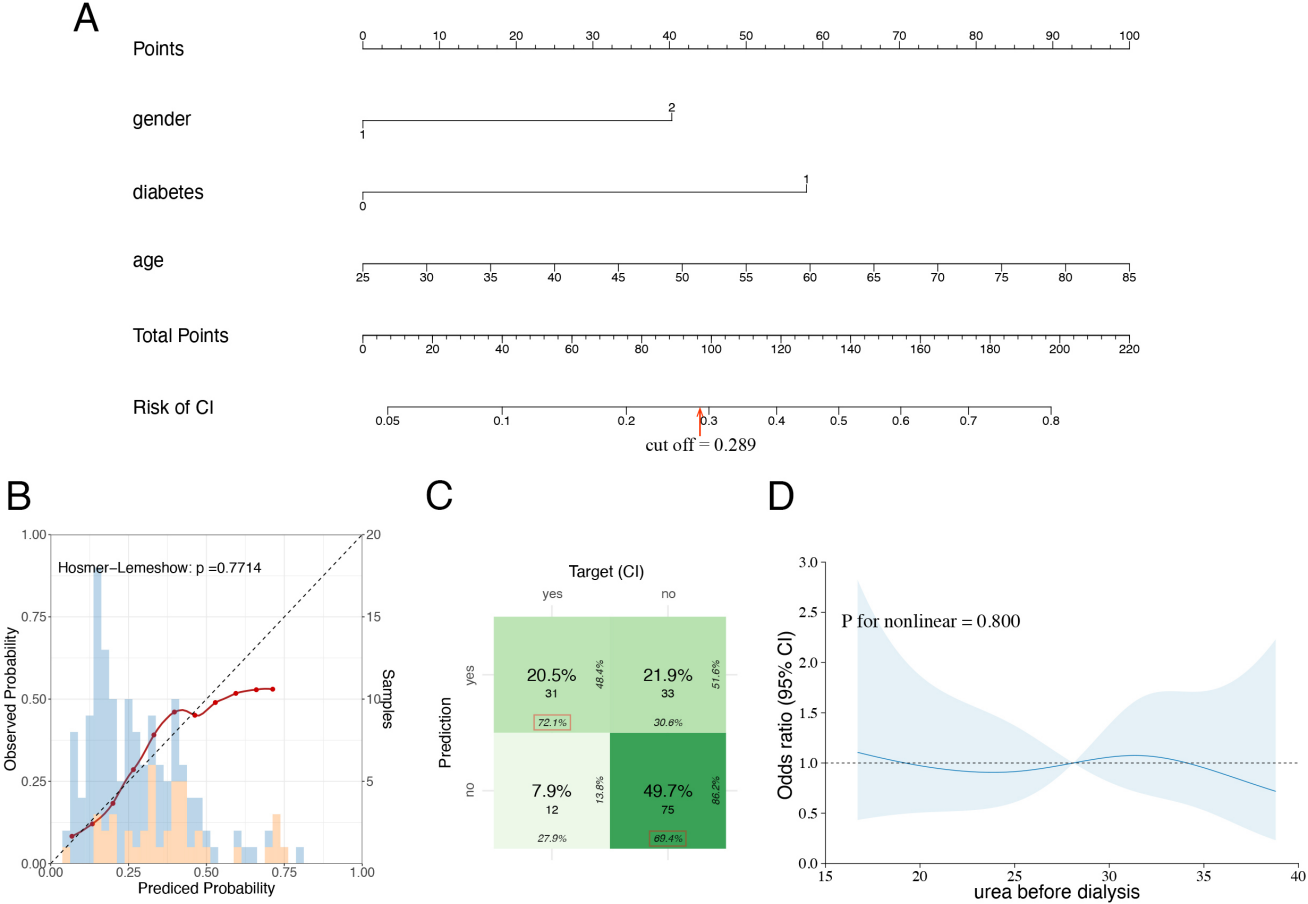
The exhibition and evaluation of model2 and RCS plot of dialysis time. **(A)** The diagnostic nomogram of CI in MHD patients(model2). **(B)** The calibration plot of model2 and Hosmer-Lemeshow test. Blue: the histogram of patients without CI; orange: the histogram of patients with CI. **(C)** The confusion matrix of model2; the cut-off value was 0.289, with a sensitivity of 0.721 and specificity of 0.694. **(D)** Restricted cubic spline plot of dialysis duration.

To enhance the presentation of the multivariate logistic regression model, a nomogram was developed for the diagnosis of CI (**Figure 4A**). By adding the points of each variable in the first line, a certain score can be obtained. Afterwards, the score can be used to determine the probability of the CI. Additionally, the H-L test of Model 2 also indicated good performance (**Figure 4B**, P = 0.771). The cut-off was set at 0.289, as determined using the Youden index, with a sensitivity of 0.721 and a specificity of 0.694 (**Figure 4C**).

In summary, in contrast to the original complex model, the simplified model has good performance, simplicity and user-friendliness.

### Dialysis duration

The impact of dialysis duration on patient health remains controversial, including in the context of CI. This study also addresses this metric. The dialysis duration for patients without a CI was 55.0 months (IQR: 26.0–105.0 months). For the CI group, the dialysis duration was 46.0 months (IQR: 24.5–83.5 months). Despite the shorter dialysis duration in the CI group, the difference was not statistically significant (P = 0.305). Additionally, univariate analysis revealed that the OR value was 0.996 (0.989, 1.003), which was not statistically significant (p = 0.268). Additionally, to further investigate the impact of dialysis duration on CI, restricted cubic spline regression was employed (**Figure 4D**).

The results indicate that the relationship between dialysis duration and CI is linear (nonlinear test: P = 0.928). Moreover, the OR remains close to 1, suggesting that dialysis duration does not significantly impact the risk of CI. This study revealed no significant association between dialysis duration and patients’ cognitive function.

## Discussion

Maintenance hemodialysis is the primary treatment for patients with ESRD. Currently, the prevention and treatment of dialysis-related complications and comorbidities have received widespread attention, among which CI significantly affects patients’ quality of life and self-care ability. Furthermore, few studies have explored the relationship between physical performance and cognitive function. Predictive models incorporating physical performance as a factor for CI prediction in MHD patients remain scarce. Therefore, this study explores the factors associated with CI, with a particular focus on the relationship between physical performance and CI.

In this study, the incidence of CI among MHD patients was 28.5%, which is lower than the 30–80% range reported in previous studies. This difference may be due in part to the exclusion of patients with severe cerebrovascular disease and sequelae in our study. To ensure the rigor of this study, patients with severe cerebrovascular diseases and sequelae were excluded to control for potential confounding effects. In addition, the clinical heterogeneity in prevalence is likely influenced by variations in population demographics, sample sizes, diagnostic criteria, and assessment methods for CI.

Consistent with findings in the general population, advanced age and female sex were identified as potential risk factors for CI among MHD patients in this study. Although the p values of these two indicators did not reach less than 0.05 in the multivariate analysis involving eight variables (Model 1), they demonstrated significant p-values in the simplified model (Model 2). These two indicators, which should not be ignored, can be used to assist in the diagnosis of CI. Ageing is strongly associated with dementia through mechanisms such as brain atrophy, amyloid-β deposition, and neuroinflammation (17). Additionally, female patients appear to be more vulnerable to CI because of hormonal changes, particularly a decrease in estrogen after menopause, which has been linked to impairments in learning and memory (18, 19). The majority of female MHD patients are postmenopausal and therefore require more attention. These findings align with those of previous studies, including those by Odagiri et al., Kurella Tamura et al. and Lee Heeryong et al., which reported similar associations in MHD populations (20–23).

Diabetes is a common comorbidity, affecting up to 45% of patients with ESKD (24). Moreover, diabetes emerged as a significant, independent risk factor for CI in our study. It can assist in predicting the CI by the original model (Model 1) or simplified model (Model 2). This may be explained by the effects of persistently high blood glucose levels, which induce oxidative stress and generate large amounts of reactive oxygen species. This process can lead to chronic cerebral ischemia, amyloid deposition, and the accumulation of toxic advanced glycation end products, ultimately causing direct neuronal damage (25, 26). Numerous studies have demonstrated that diabetes is associated with an increased risk of general or specific CI in ESKD patients undergoing hemodialysis, peritoneal dialysis, or kidney transplantation (27–29). Similarly, this study revealed that diabetes increased the risk of CI in MHD patients. This may also be related to the absolute or relative insufficiencies of insulin secretion in diabetic patients. Indeed, insulin also plays important roles in neuronal synaptic plasticity and facilitates learning and memory in humans; therefore, impaired insulin signaling can contribute directly to neuronal dysfunction and degeneration (30). Furthermore, insulin regulates cerebral bioenergetics, increases synaptic activity and dendritic spine formation, and increases the turnover of neurotransmitters (31). Insulin receptors and components of the insulin signaling pathway are widely distributed in the brain, especially in cognition-related regions, such as the cerebral cortex, olfactory bulb, hippocampus, and hypothalamus (32). On the basis of these findings, effective diabetes management and brain insulin resistance could be therapeutic targets to help control CI, which could be meaningful for its treatment.

Moreover, this study revealed that lower HS, longer TUGT, and slower GS were associated with CI. Patients with CI exhibited poorer physical performance, suggesting that better physical performance may serve as a protective factor against cognitive impairment. A meta-analysis reported a strong link between CKD progression and reduced walking speed (33). Another meta-analysis of longitudinal observational studies (1–21 years) revealed that regular exercise was associated with a reduced risk of dementia (34). Yu Ho Lee et al. demonstrated that MHD patients with low GS and HS scores exhibited significantly impaired cognitive function, as assessed by the MMSE and KDQOL-SF (35). Similarly, Jyotish Chalil Gopinathan et al. reported that MHD patients with CI had a significantly greater prevalence of frailty, including weak grip strength and slow walking speed (36). Moreover, another study revealed that 12 HD patients who received three months of tablet-based cognitive training during dialysis experienced improvements in MMSE scores, Montreal Cognitive Assessment (MoCA) scores, and executive function (9). However, in the multivariate logistic regression analysis, physical performance did not reach statistical significance. Although physical performance may aid in the diagnosis of CI, it cannot be considered an independent risk factor. Some studies have suggested that low muscle mass and strength can be improved through exercise, and physical activity in MHD patients has been shown to improve vascular function by reducing systemic inflammation, oxidative stress, and arterial stiffness (37). Nevertheless, these conclusions should be interpreted with caution in the context of this study. These differences may arise from variations in the study population and inclusion criteria. Moreover, relevant cohort studies are lacking. Therefore, the relationship between physical performance and CI requires further investigation. However, as this study was confined to individuals with CI and did not include individuals with mild cognitive impairment (MCI), the relationships between these three indicators and MCI warrant further investigation.

In addition, the association between dialysis duration and CI remains controversial. Currently, there is a perspective suggesting that with increasing dialysis duration, repeated circulatory stress during hemodialysis may contribute to ischemic brain injury. This is largely due to recurrent cerebral hypoperfusion during hemodialysis, which can accelerate cognitive decline (38). Ding Chen et al. reported that a dialysis duration of ≥5 years was associated with an increased risk of CI (16). In contrast, there was no statistically significant difference in dialysis duration between the CI group and the non-CI group in this study. Furthermore, these findings were validated through univariate analysis and restricted cubic spline regression. Using multiple analytical approaches, this study ultimately revealed that dialysis duration was not associated with CI. Therefore, controversy remains concerning the association between hemodialysis duration and CI in MHD patients. However, each dialysis session differs in terms of frequency, adequacy, dialysate temperature, and even the type of dialyzer, all of which may influence dialysis outcomes. Therefore, further longitudinal studies may be needed to clarify the relationship between hemodialysis duration and CI.

Beyond these, some other factors also deserve attention. Several studies have shown that lower educational levels are associated with CI (39, 40). However, we found that patients with more than 12 years of education were less likely to have CI (OR: 0.139; 95% CI: 0.007–0.801; P = 0.069). This may be explained by the link between lower education levels and reduced cognitive and functional reserves. However, education does not have statistical significance. Stroke was not a significant risk factor in our cohort, possibly because of the exclusion of patients with severe cerebrovascular disease.

Interestingly, this study revealed no difference in the percentage of ultrafiltration volume between patients with CI and those without CI. Hemodialysis results in rapid fluid shifts that can often lead to wide swings in blood pressure (41). A possible explanation for this could be that timely treatment was administered to patients when their blood pressure decreased. Further exploration of the relationship between changes in circulating volume and their impact on CI in MHD patients may be valuable.

In summary, this study explored the factors related to CI, and a simplified predictive model was developed. This model not only provides a practical and user-friendly tool for clinical application but also offers valuable insights into the potential risk factors for CI. By incorporating multiple analytical approaches, this study enhances the understanding of CI in MHD patients and offers assistance for future research in this area.

Finally, this study has several limitations. First, patients who were unable to complete the physical performance assessments were excluded, which may have resulted in a biased sample. Second, although the MMSE offers high clinical utility and efficiency for global cognitive screening, its well-documented limitations in sensitivity for mild cognitive impairment (MCI) must be acknowledged. Its speed and simplicity come at the cost of insufficient assessment of executive functions and complex attention, leading to potential under detection of early cognitive decline and possible underestimation of effects in this study. Future studies should incorporate more sensitive tools, such as the Montreal Cognitive Assessment (MoCA), or detailed neuropsychological batteries to improve the accurate identification of MCI and ensure a more comprehensive evaluation of cognitive domains. Finally, its single-center, cross-sectional design hinders generalizability and prevents the establishment of causal relationships. Therefore, a prospective cohort study design is recommended. The sample size, although adequate for initial analysis, may limit the statistical power for detecting subtle effects. Due to the limitation of single-center, future multicenter studies with larger cohorts and longitudinal designs are needed to validate these findings and explore causality.

## Conclusions

In conclusion, this study revealed a high incidence of cognitive impairment (CI) among MHD patients. A predictive model for CI was developed using eight variables: age, sex, stroke status, HS, TUGT results, GS, diabetes status, and education level. To improve clinical applicability, a simplified model incorporating only diabetes, age, and sex was constructed, which demonstrated good diagnostic accuracy while being more practical for use in clinical settings. Additionally, diabetes was identified as an independent risk factor for CI, whereas dialysis duration was not found to be associated with CI. These findings provide valuable insights into the risk factors for CI in MHD patients and offer a practical tool to aid in early identification and intervention.

## Data Availability

The raw data supporting the conclusions of this article will be made available by the authors, without undue reservation.

## Supplementary material

The Supplementary Material for this article can be found onlineat: https://www.frontiersin.org/articles/10.3389/fendo.2025.1594605/full#supplementary-material
