# Supplementary material for: Prediction model for cognitive impairment in maintenance hemodialysis patients: the role of diabetes
Source: Front Endocrinol (Lausanne). 2025 Nov 26;16:1594605. doi: 10.3389/fendo.2025.1594605 (PMC12689383; doi:10.3389/fendo.2025.1594605)
Supplement: Supplementary file 1 [file DataSheet1.zip › Supplement/Suppl.Fig.docx]

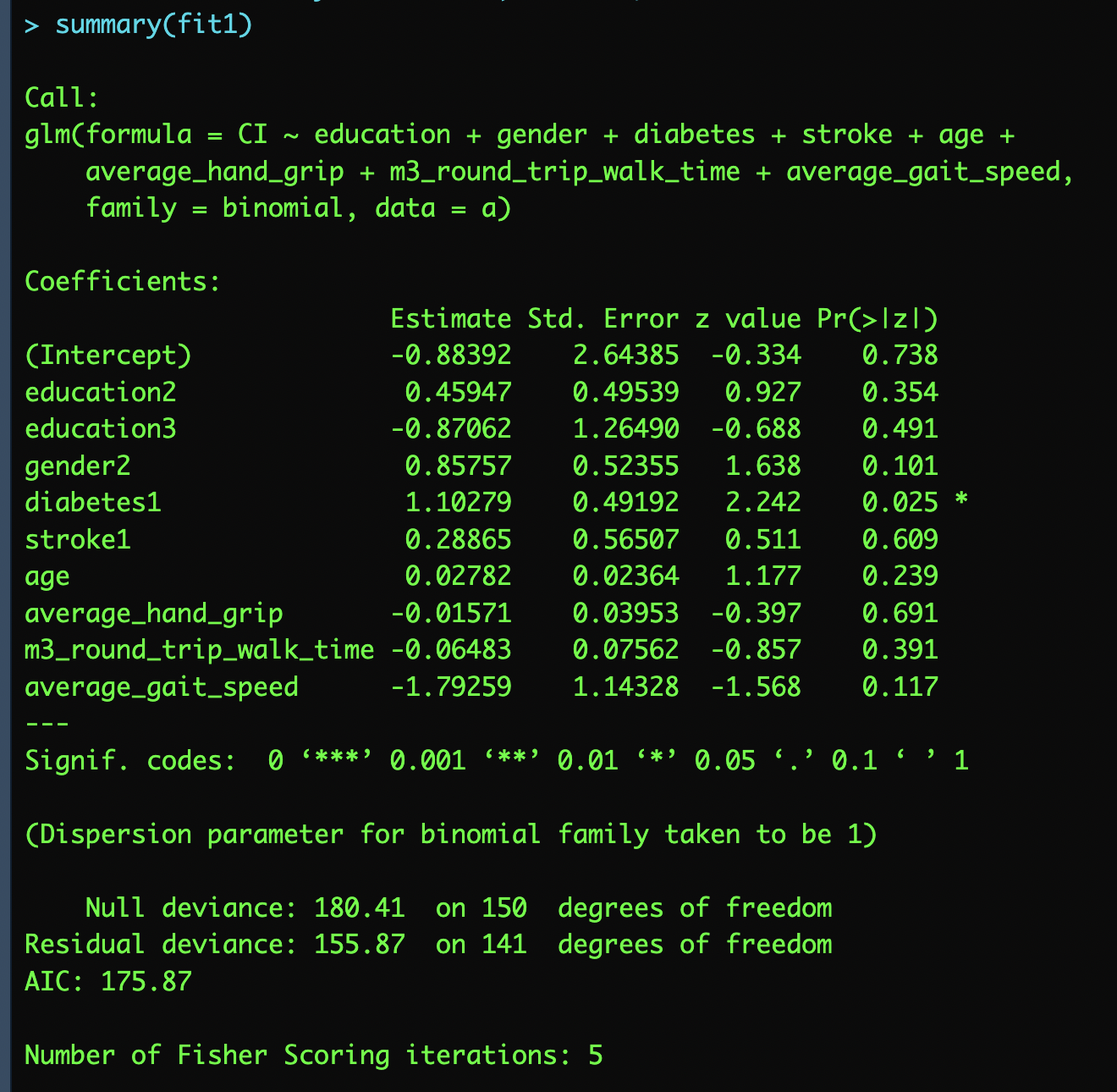


Fig.S1: The formula, OR and p value of model1.


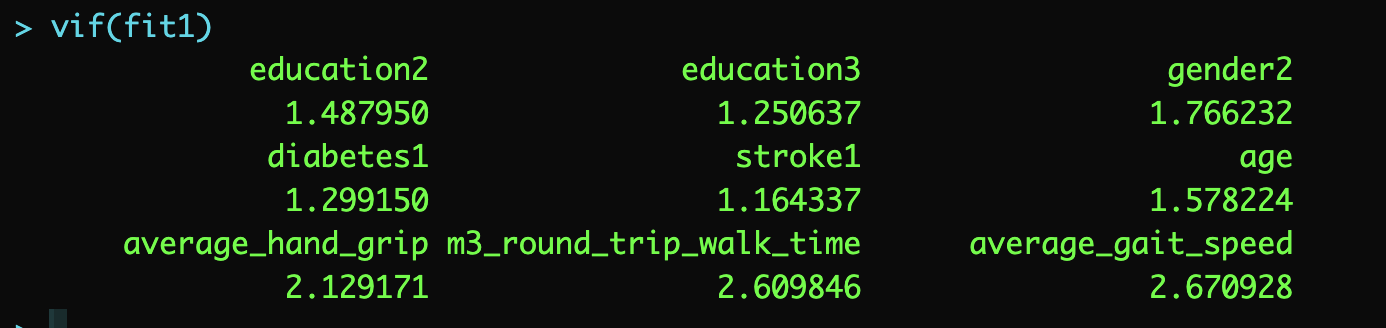


Fig.S2: The variance inflation factor(VIF) of model1, which was used to assess the degree of multicollinearity present in a multiple regression model.


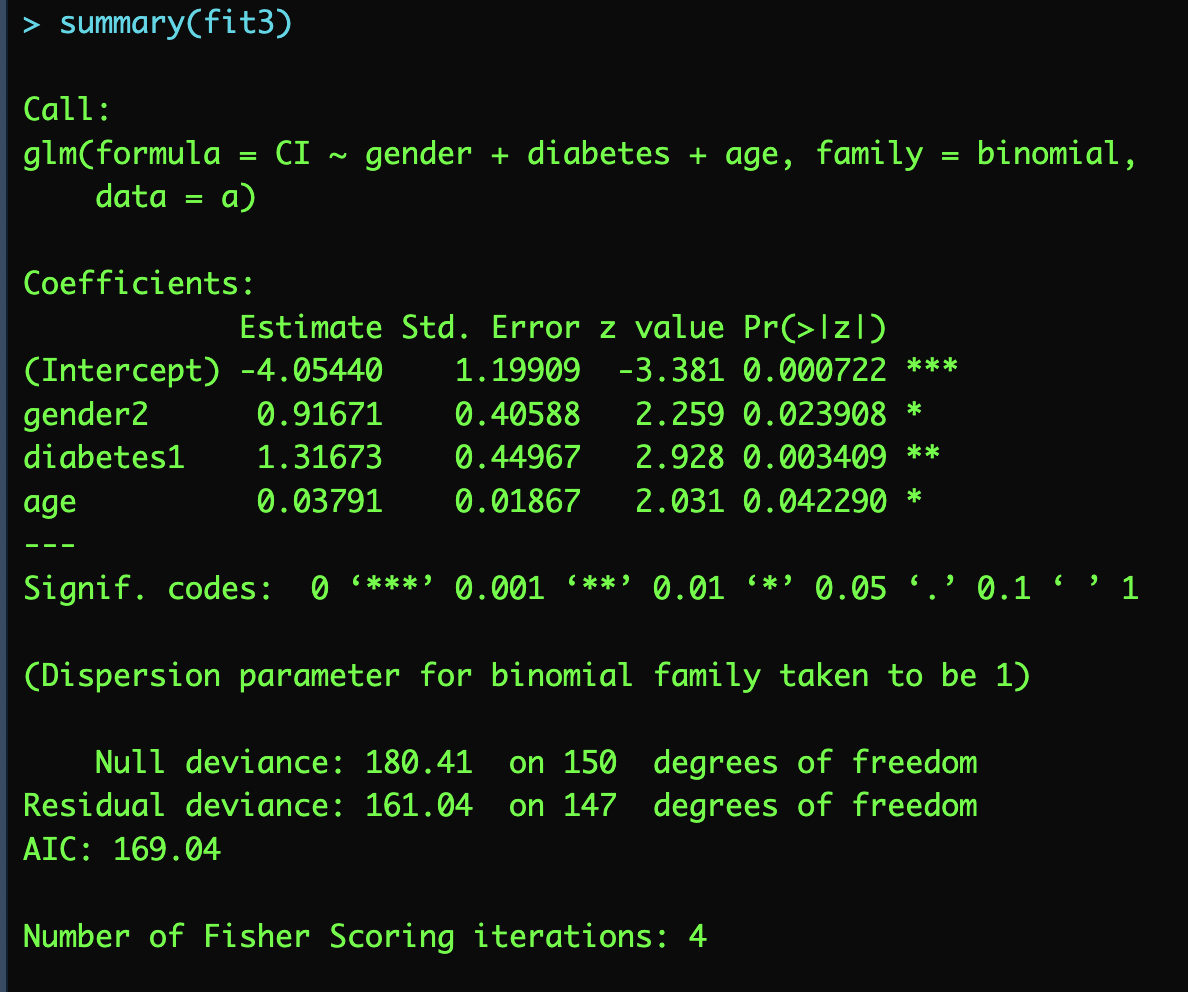


Fig.S3: The formula, OR and p value of model2.


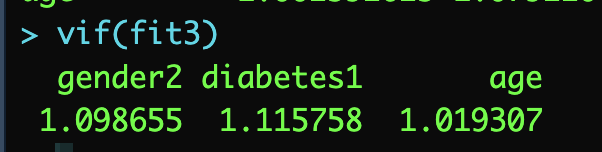


Fig.S4: The variance inflation factor(VIF) of model2.


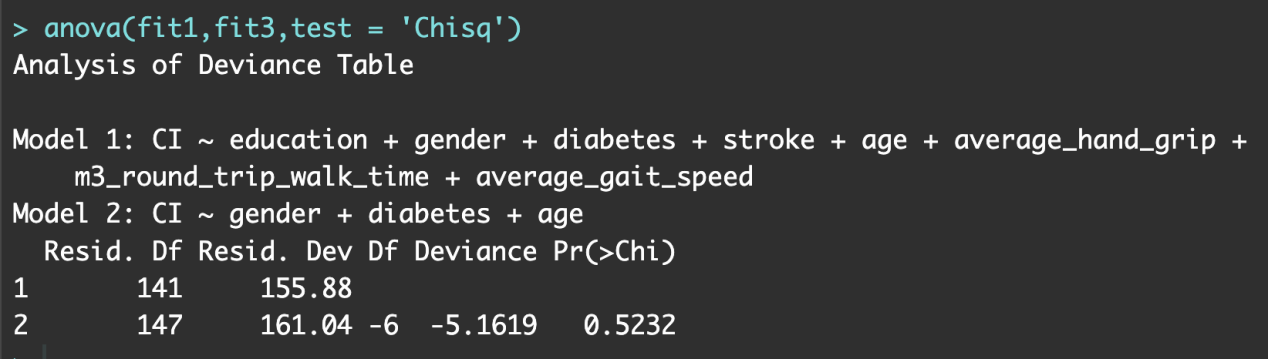


Fig.S5: The wilcoxon test between model1(fit1) and model2(fit3), which compared the predictive outcomes to assess differences.


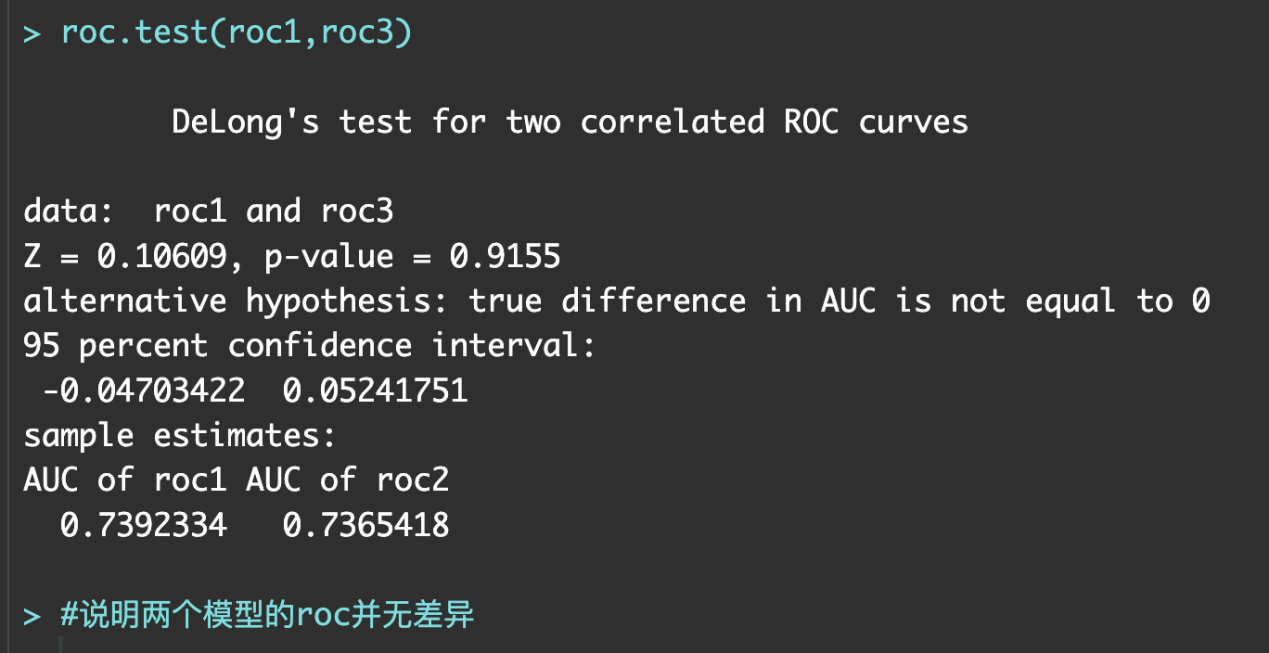


Fig.S6: The test for two ROC curves between model1(roc1) and model2(roc3).


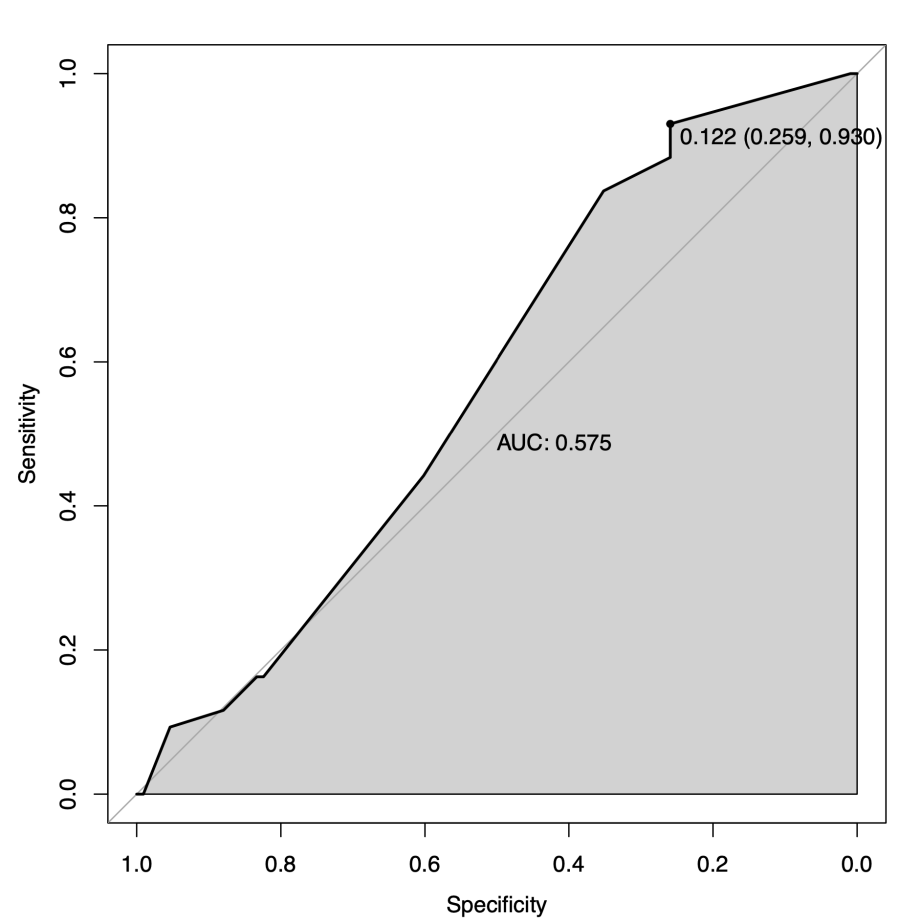


Fig.S7: The ROC curves of model developed by Chen et al.
