# Supplementary figures and images for: Prediction model for cognitive impairment in maintenance hemodialysis patients: the role of diabetes
Source: Front Endocrinol (Lausanne). 2025 Nov 26;16:1594605. doi: 10.3389/fendo.2025.1594605 (PMC12689383; doi:10.3389/fendo.2025.1594605)

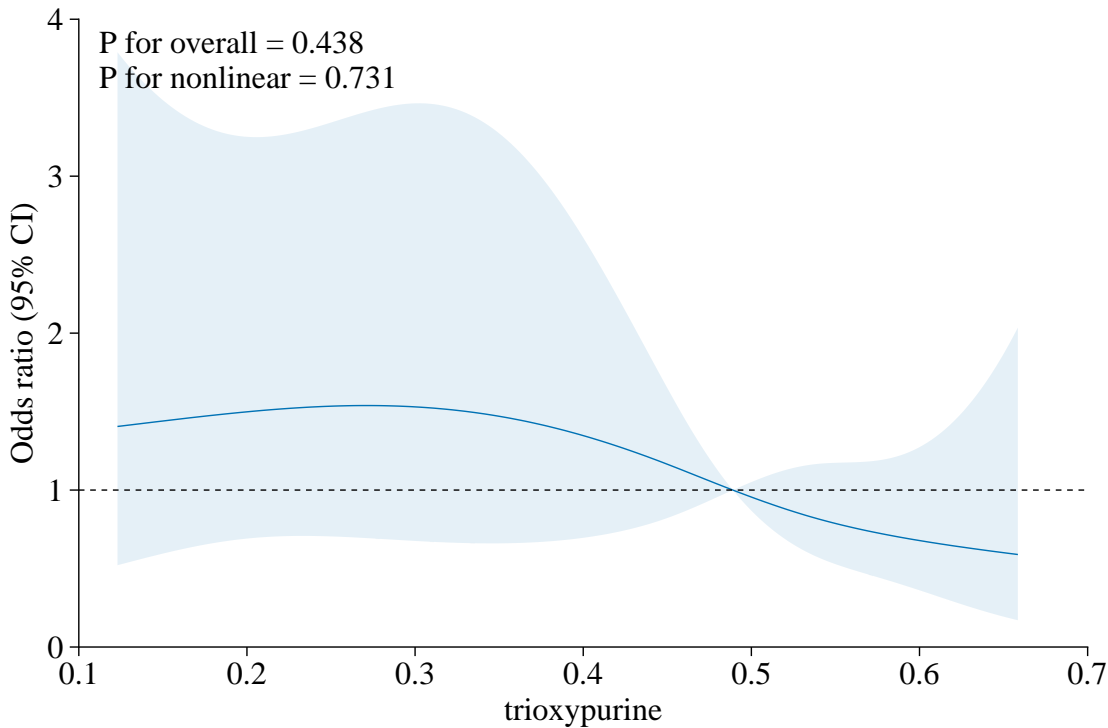

Supplement: Supplementary file 1 [file DataSheet1.zip › Supplement/RCS/trioxypurine.pdf]

P for overall = 0.954  
P for nonlinear = 0.902

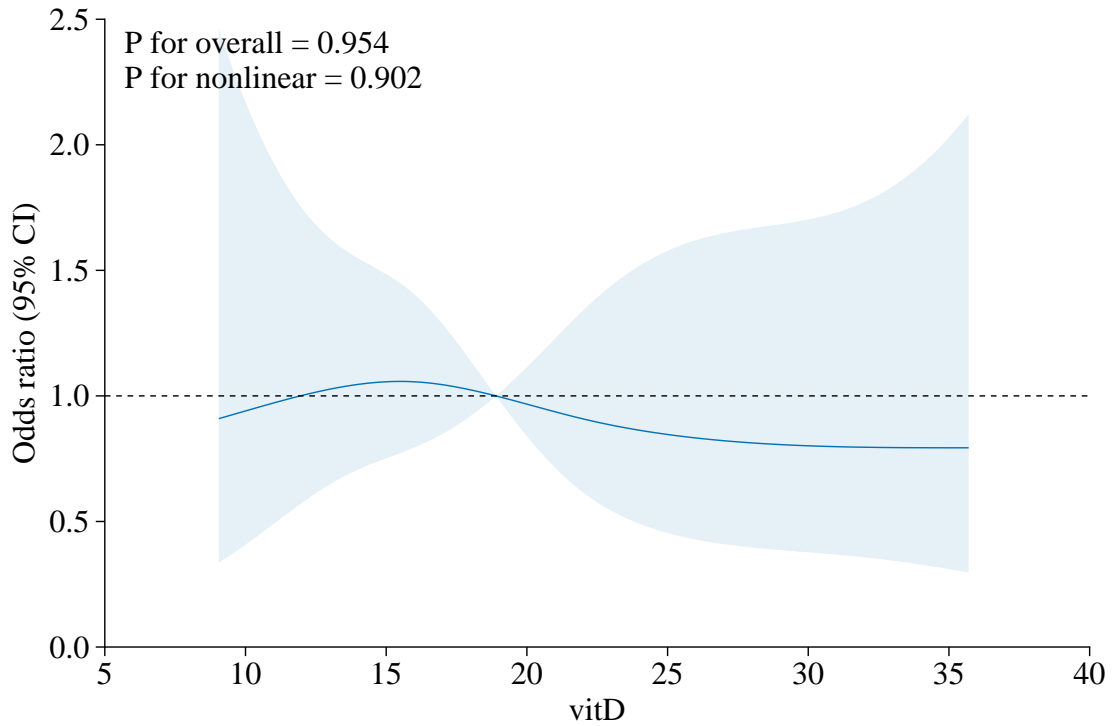

Supplement: Supplementary file 1 [file DataSheet1.zip › Supplement/RCS/vitD.pdf]

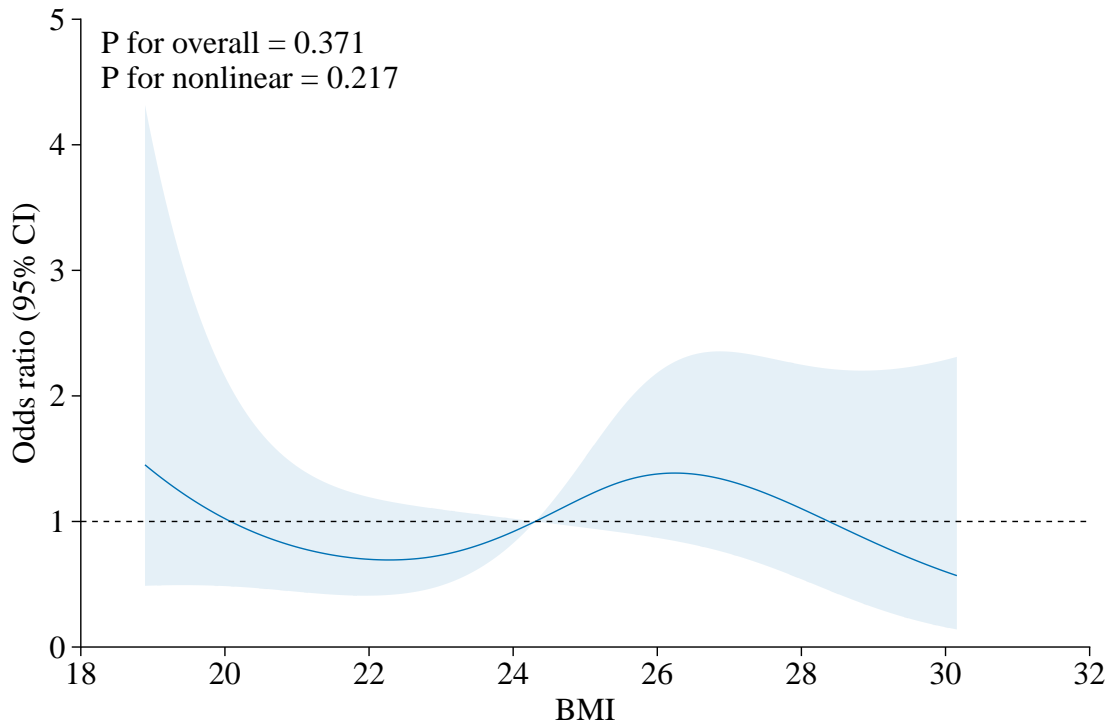

Supplement: Supplementary file 1 [file DataSheet1.zip › Supplement/RCS/BMI.pdf]

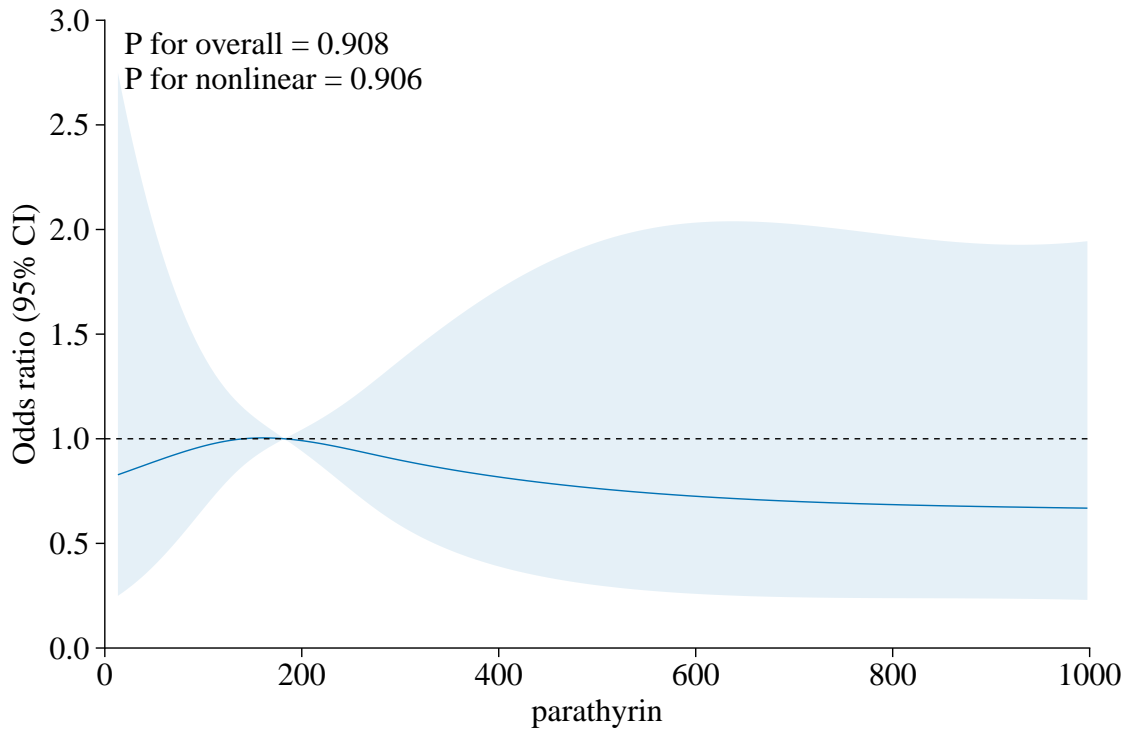

Supplement: Supplementary file 1 [file DataSheet1.zip › Supplement/RCS/parathyrin.pdf]

P for overall = 0.885  
P for nonlinear = 0.765

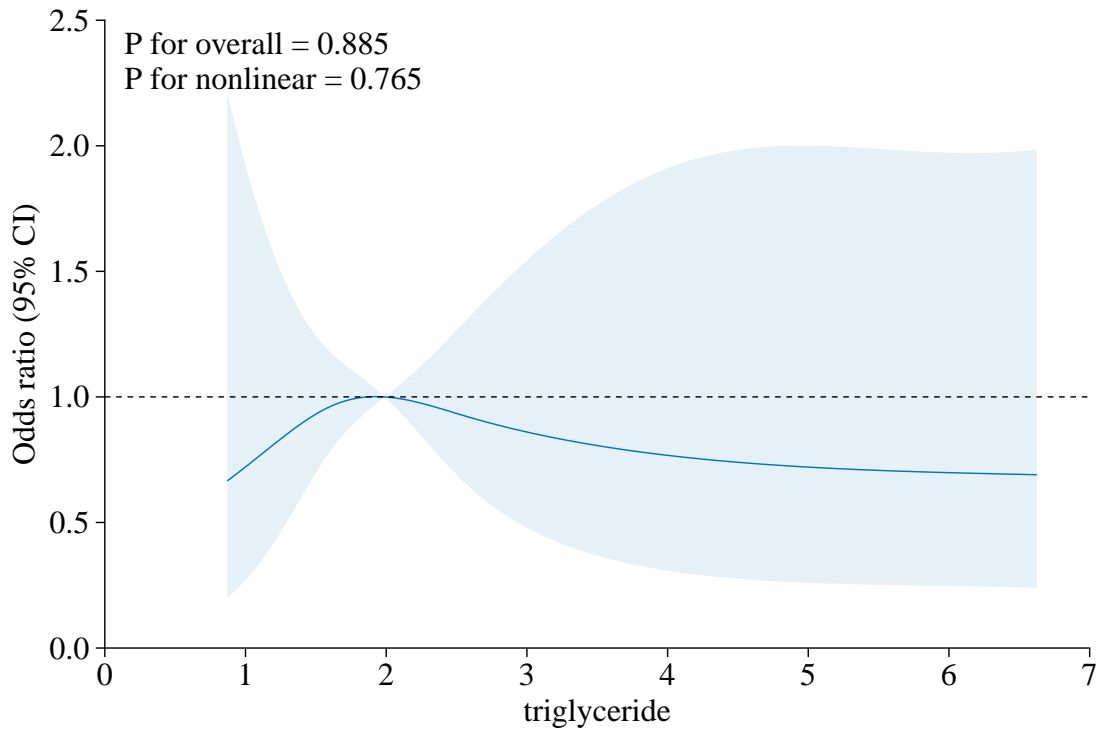

Supplement: Supplementary file 1 [file DataSheet1.zip › Supplement/RCS/triglyceride.pdf]

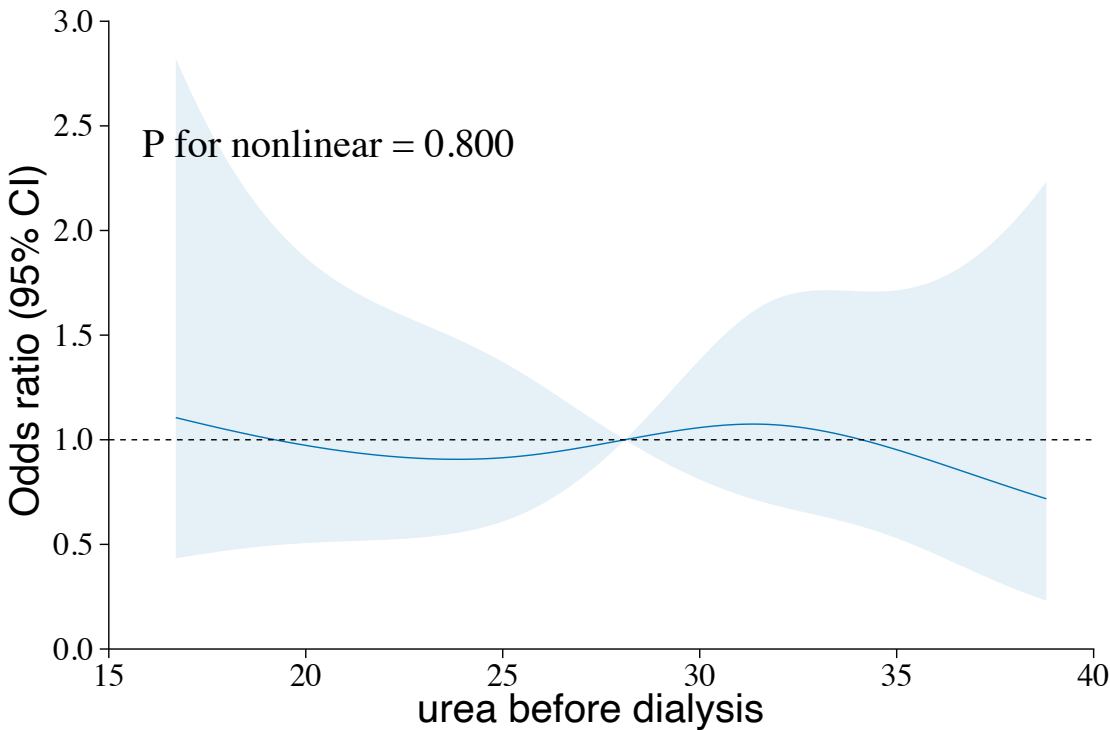

Supplement: Supplementary file 1 [file DataSheet1.zip › Supplement/RCS/urea_before_dialysis.pdf]

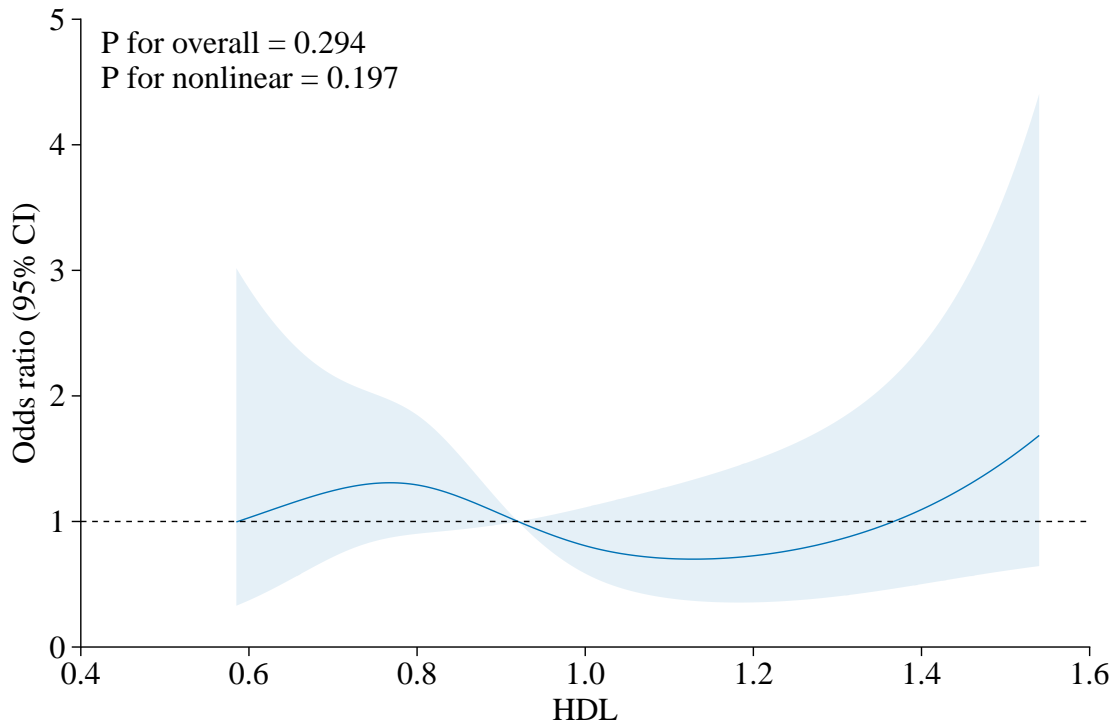

Supplement: Supplementary file 1 [file DataSheet1.zip › Supplement/RCS/HDL.pdf]

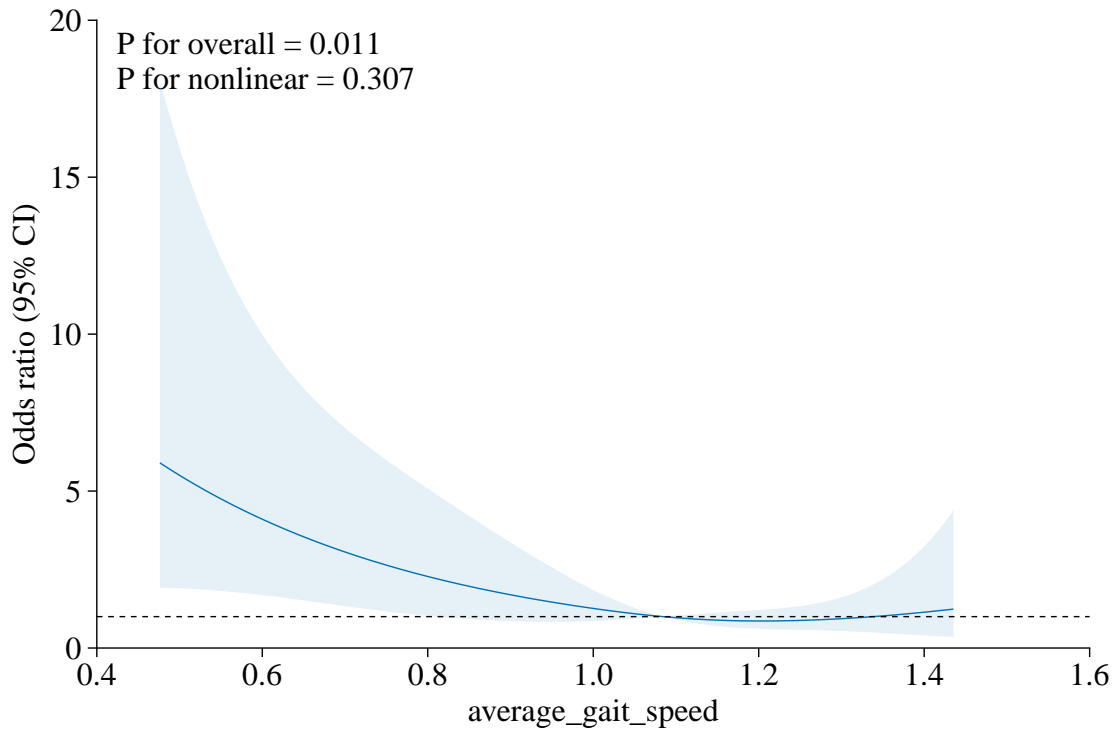

Supplement: Supplementary file 1 [file DataSheet1.zip › Supplement/RCS/average_gait_speed.pdf]

P for overall = 0.821  
P for nonlinear = 0.920

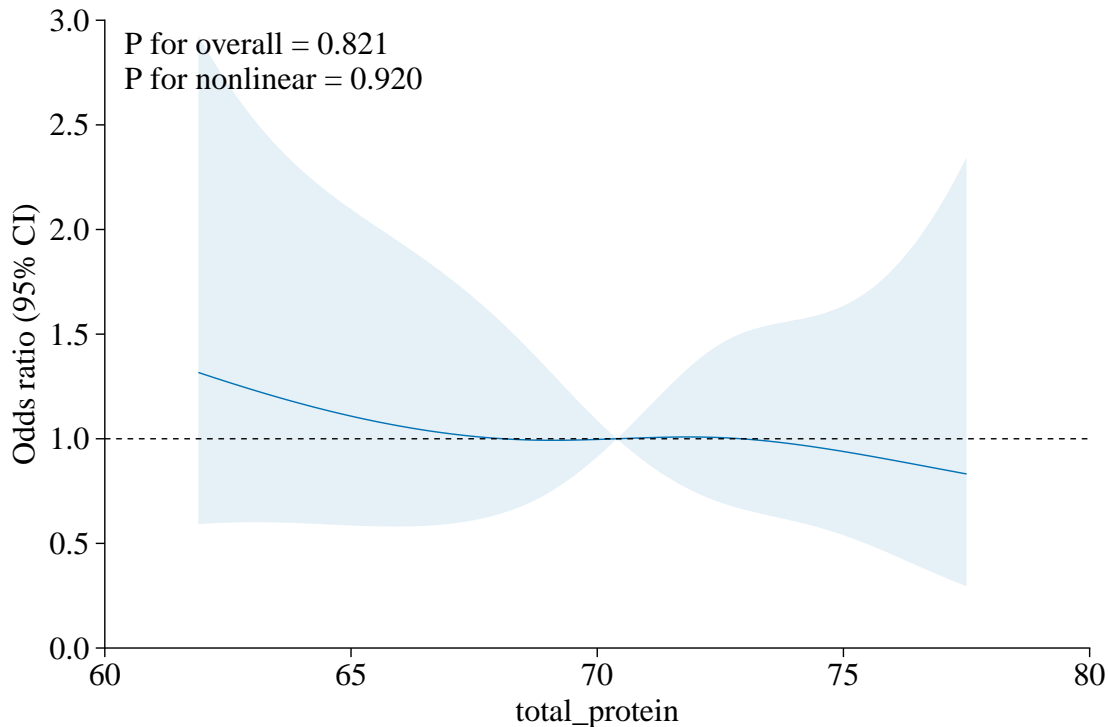

Supplement: Supplementary file 1 [file DataSheet1.zip › Supplement/RCS/total_protein.pdf]

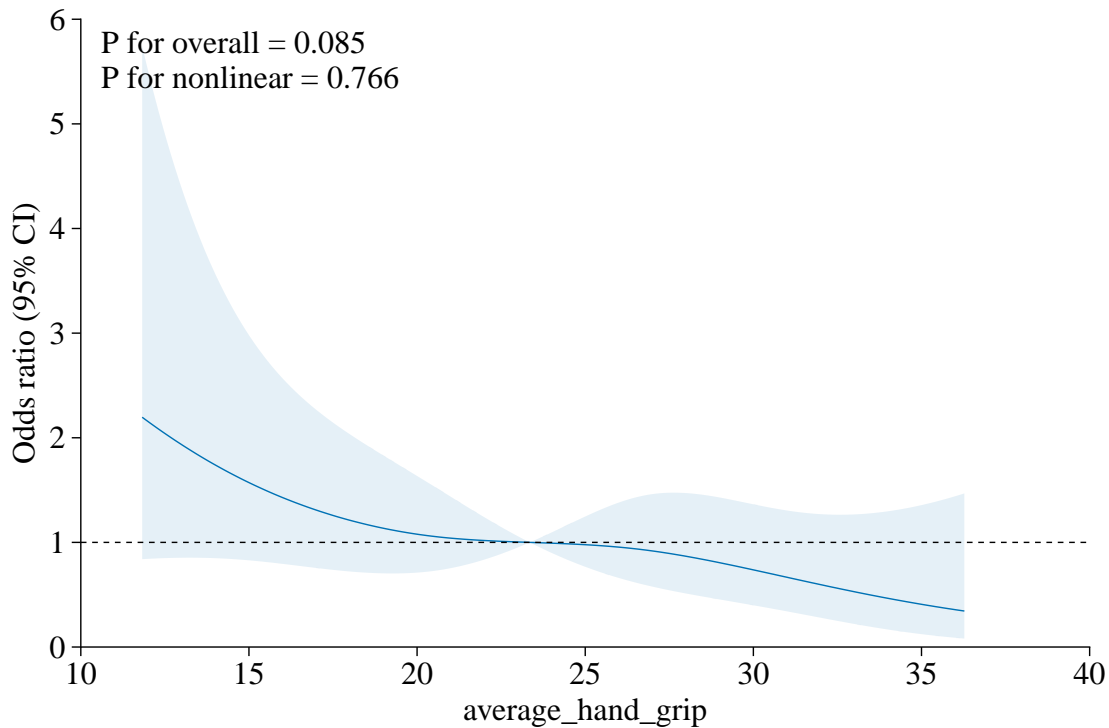

Supplement: Supplementary file 1 [file DataSheet1.zip › Supplement/RCS/average_hand_grip.pdf]

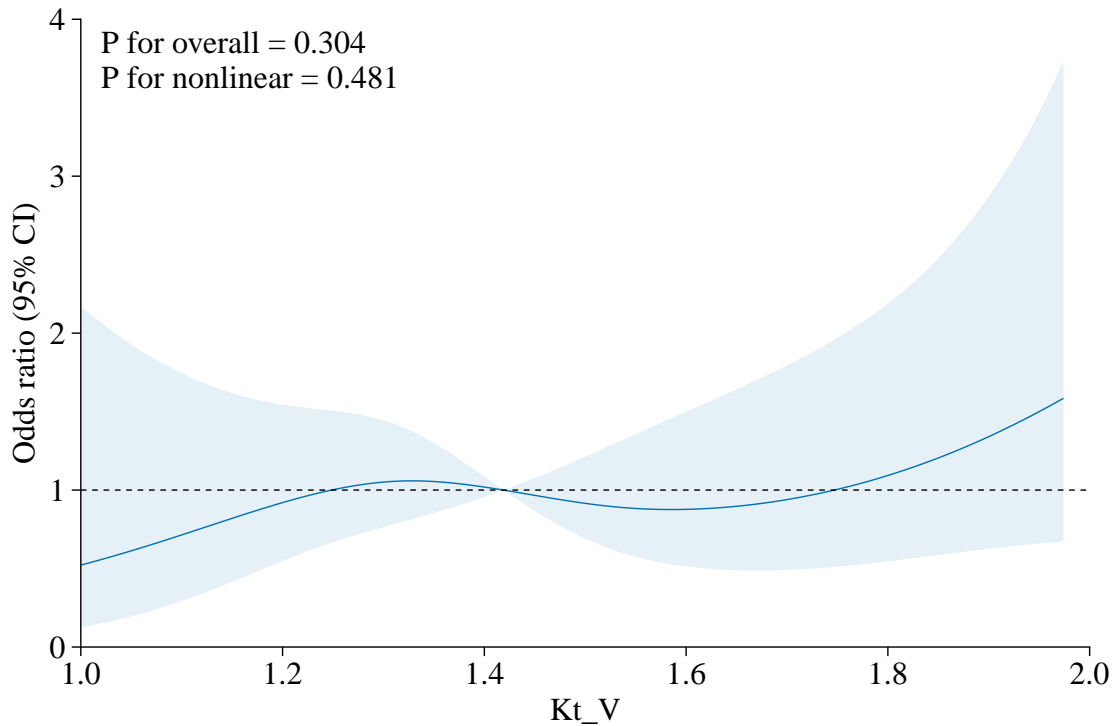

Supplement: Supplementary file 1 [file DataSheet1.zip › Supplement/RCS/Kt_V.pdf]

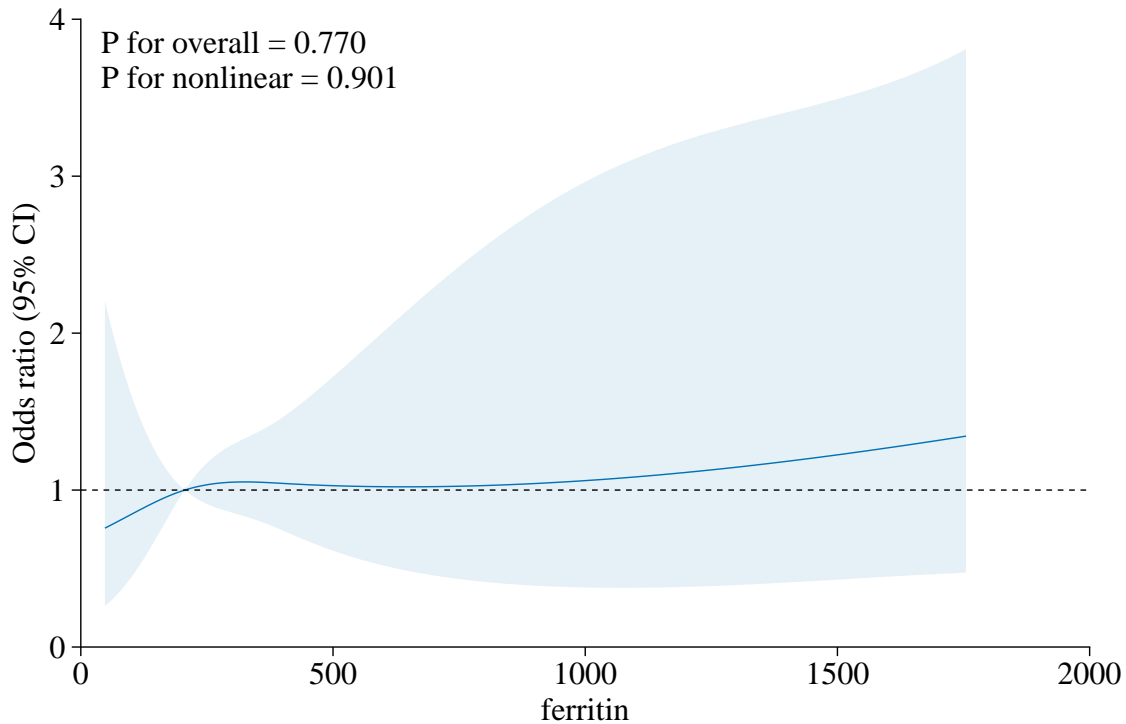

Supplement: Supplementary file 1 [file DataSheet1.zip › Supplement/RCS/ferritin.pdf]

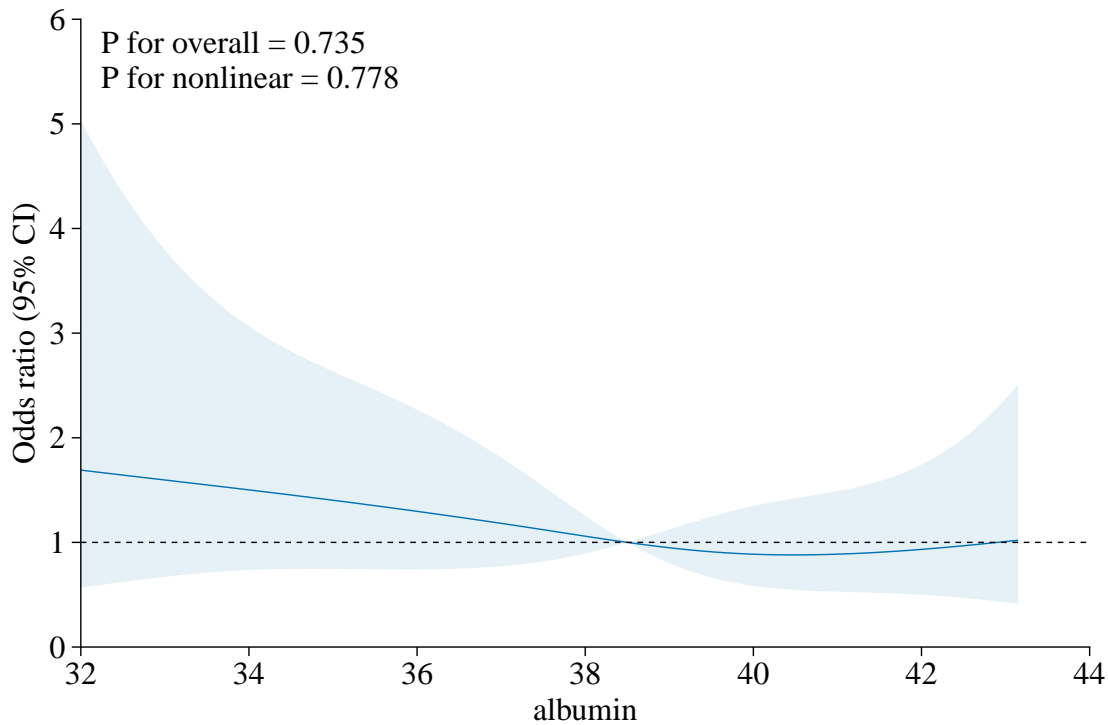

Supplement: Supplementary file 1 [file DataSheet1.zip › Supplement/RCS/albumin.pdf]

P for overall = 0.178  
P for nonlinear = 0.087

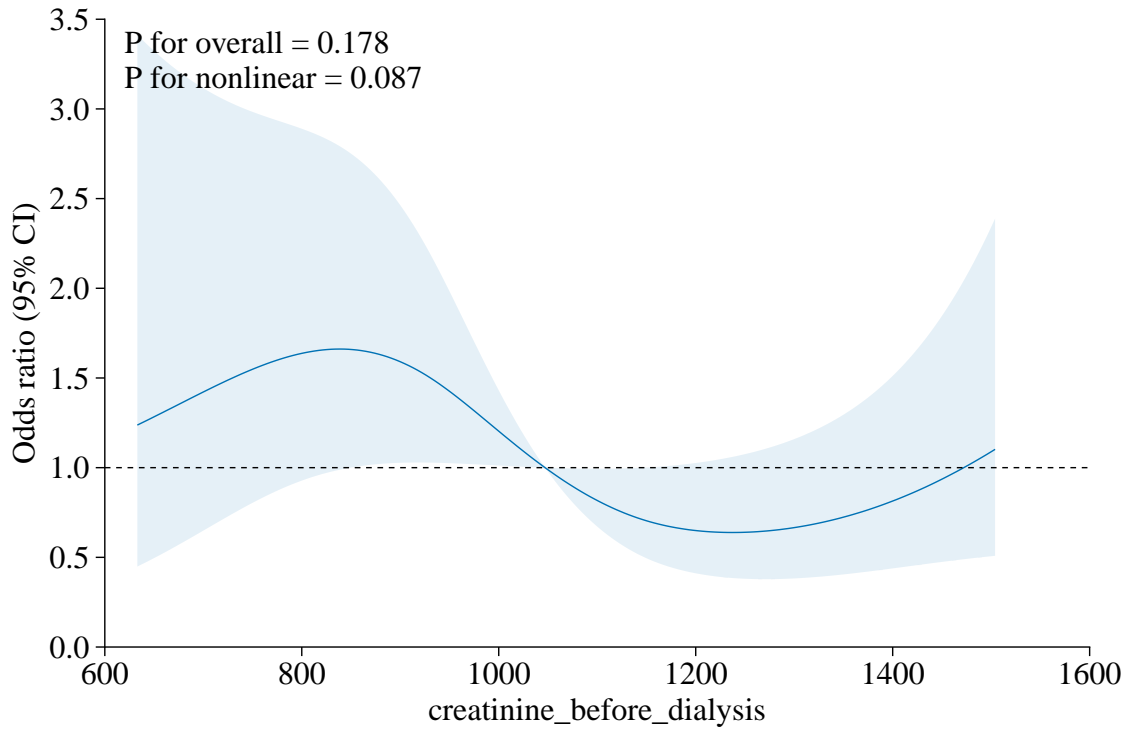

Supplement: Supplementary file 1 [file DataSheet1.zip › Supplement/RCS/creatinine_before_dialysis.pdf]

P for overall = 0.874  
P for nonlinear = 0.708

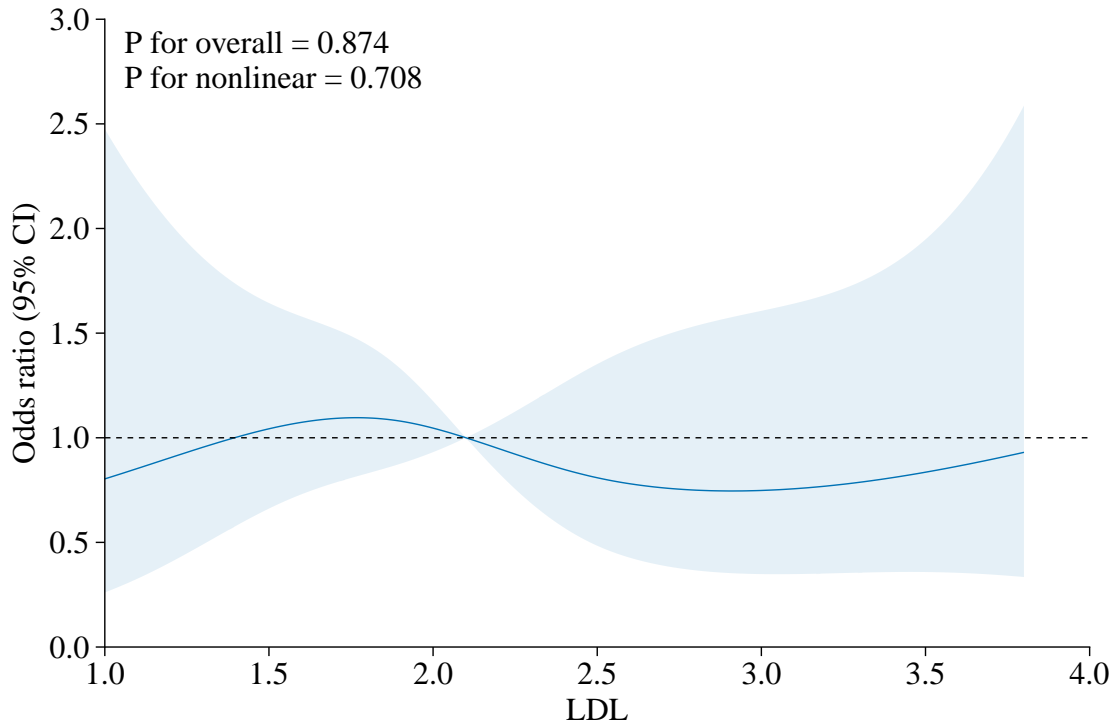

Supplement: Supplementary file 1 [file DataSheet1.zip › Supplement/RCS/LDL.pdf]

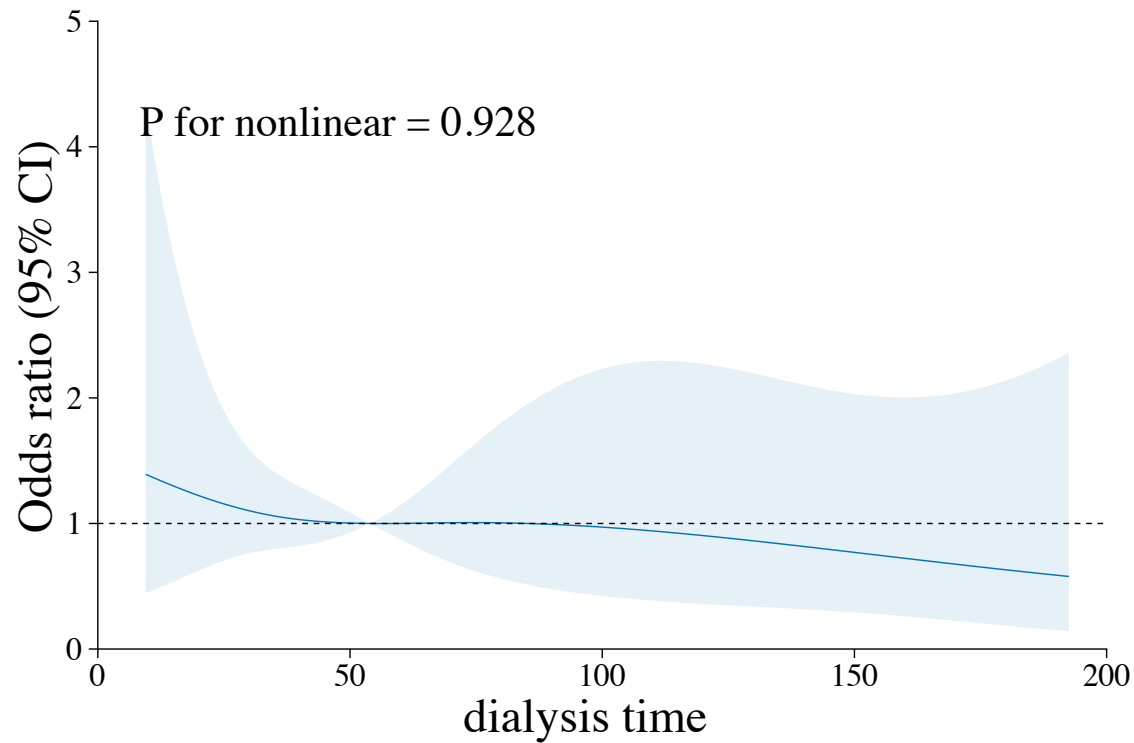

Supplement: Supplementary file 1 [file DataSheet1.zip › Supplement/RCS/dialysis_time.pdf]

P for overall = 0.328  
P for nonlinear = 0.229

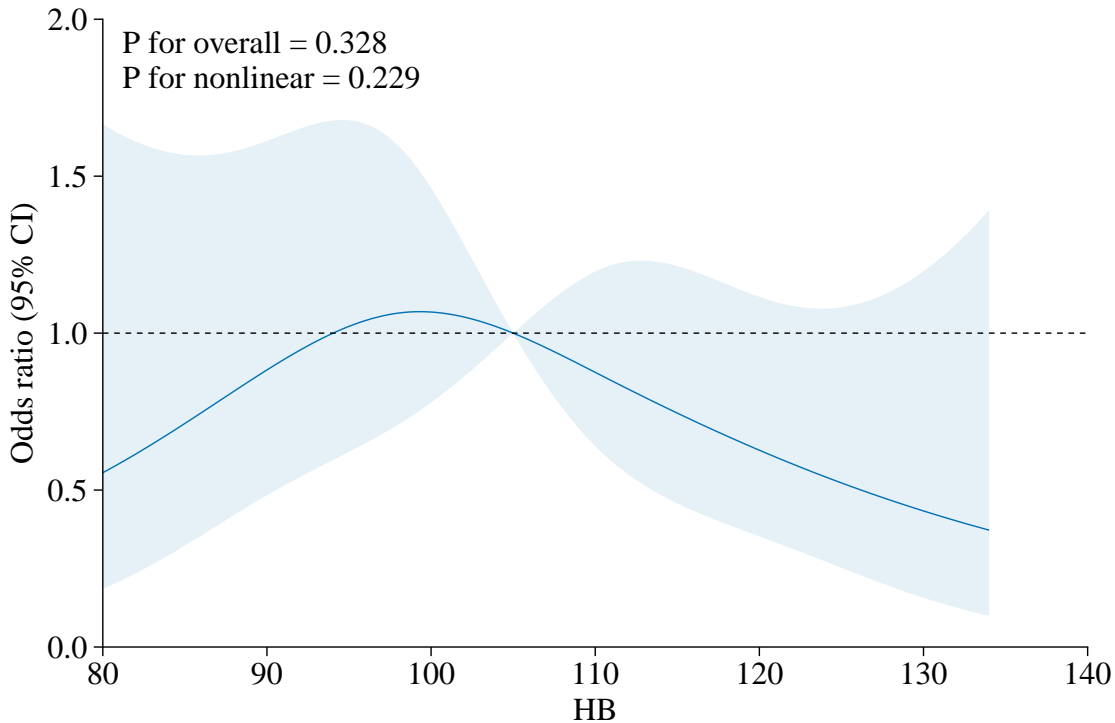

Supplement: Supplementary file 1 [file DataSheet1.zip › Supplement/RCS/HB.pdf]

P for overall = 0.614  
P for nonlinear = 0.579

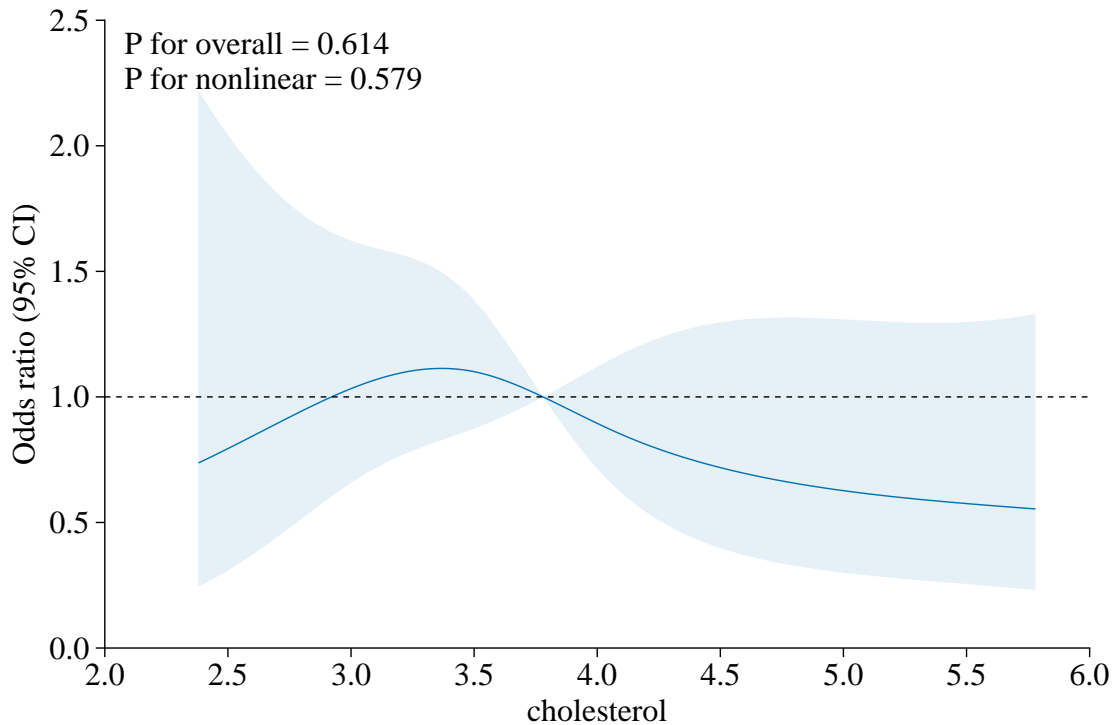

Supplement: Supplementary file 1 [file DataSheet1.zip › Supplement/RCS/cholesterol.pdf]

P for overall = 0.850  
P for nonlinear = 0.944

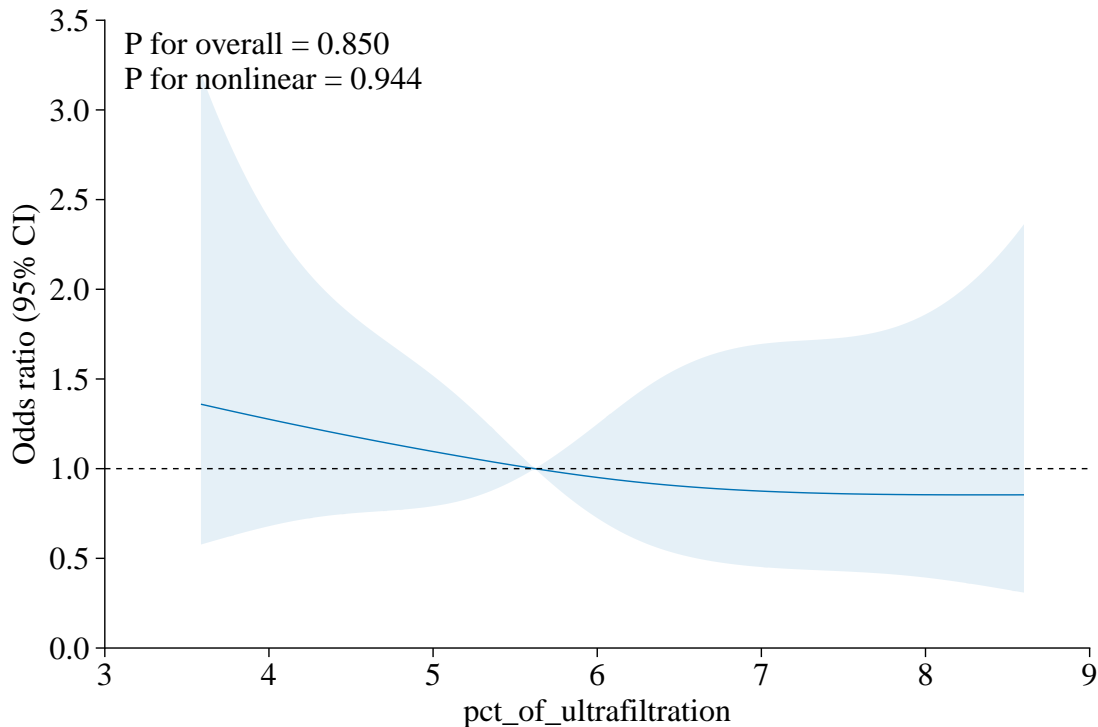

Supplement: Supplementary file 1 [file DataSheet1.zip › Supplement/RCS/pct_of_ultrafiltration.pdf]

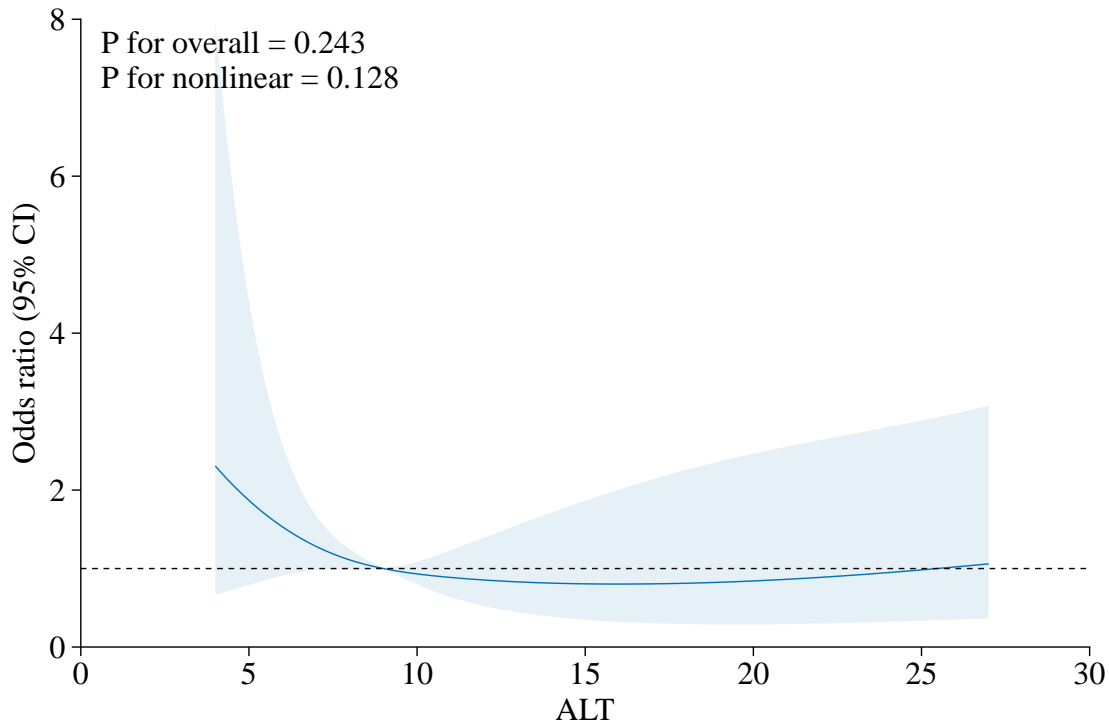

Supplement: Supplementary file 1 [file DataSheet1.zip › Supplement/RCS/ALT.pdf]

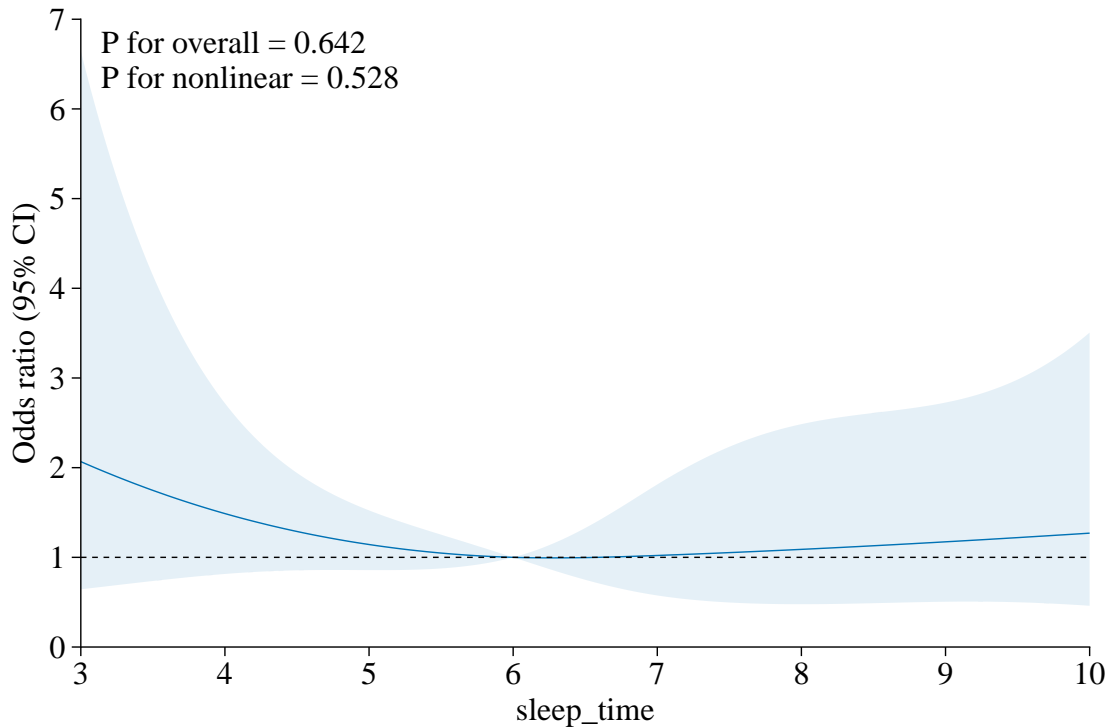

Supplement: Supplementary file 1 [file DataSheet1.zip › Supplement/RCS/sleep_time.pdf]

P for overall = 0.031  
P for nonlinear = 0.212

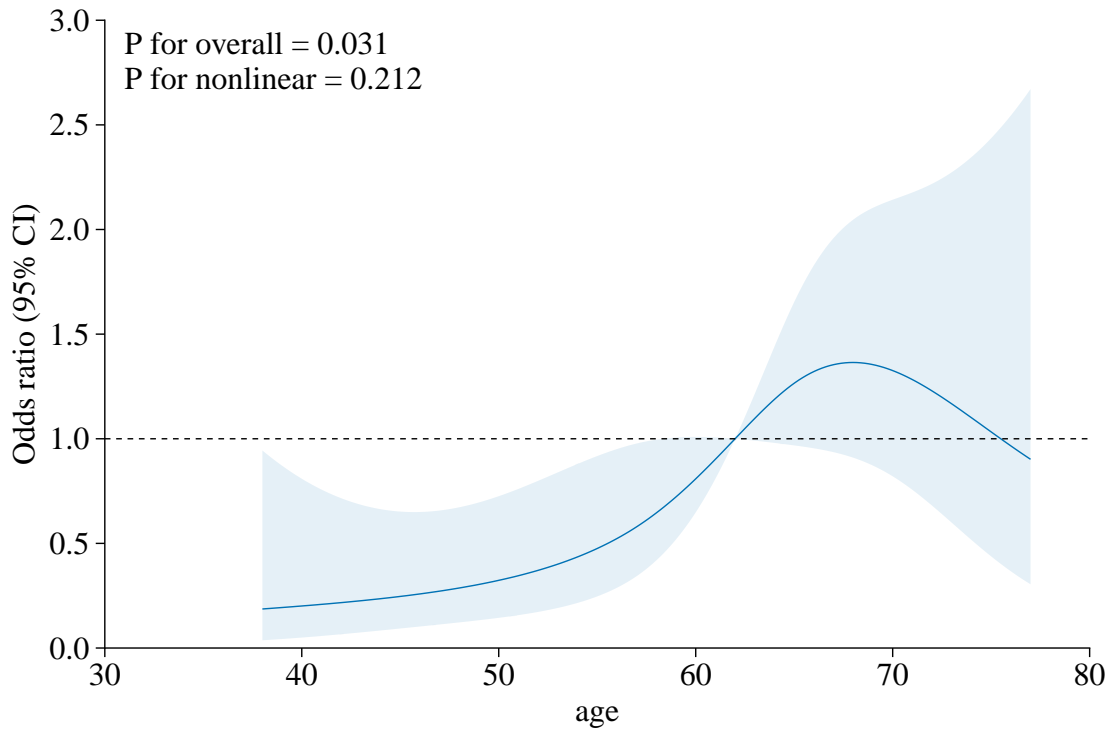

Supplement: Supplementary file 1 [file DataSheet1.zip › Supplement/RCS/age.pdf]

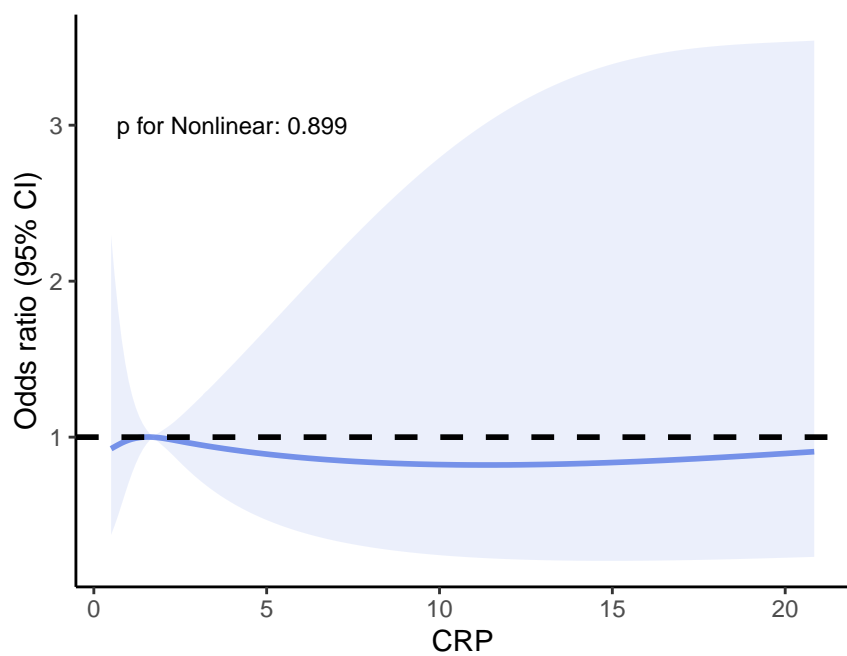

Supplement: Supplementary file 1 [file DataSheet1.zip › Supplement/RCS/CRP.pdf]

P for overall = 0.309  
P for nonlinear = 0.410

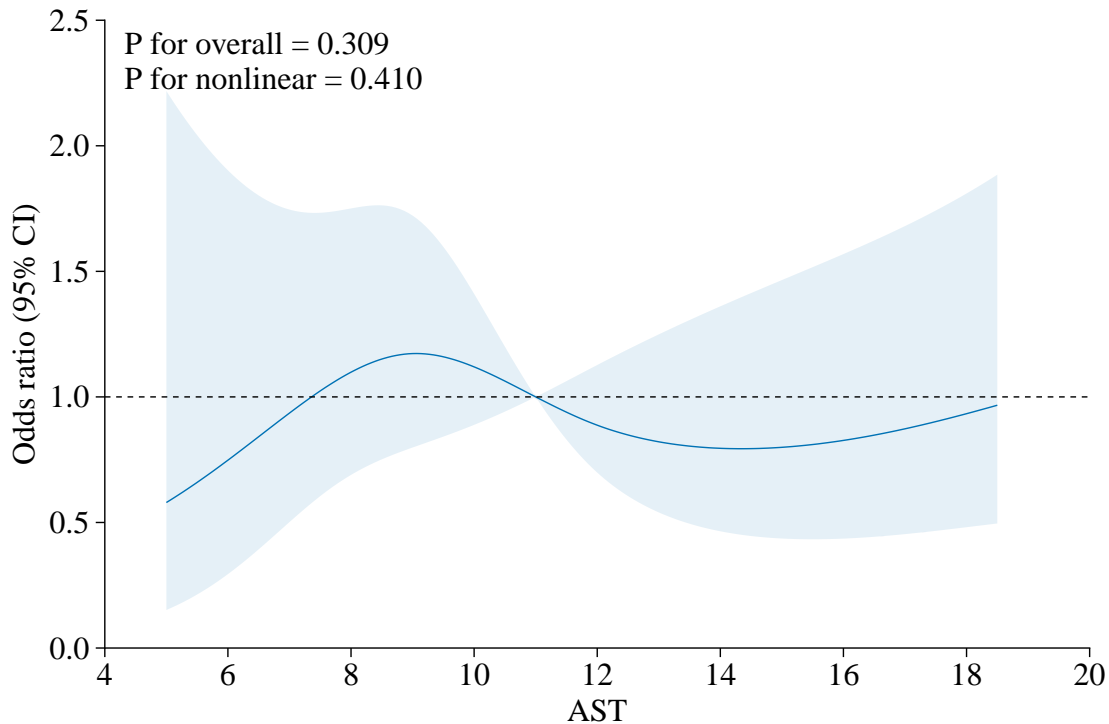

Supplement: Supplementary file 1 [file DataSheet1.zip › Supplement/RCS/AST.pdf]

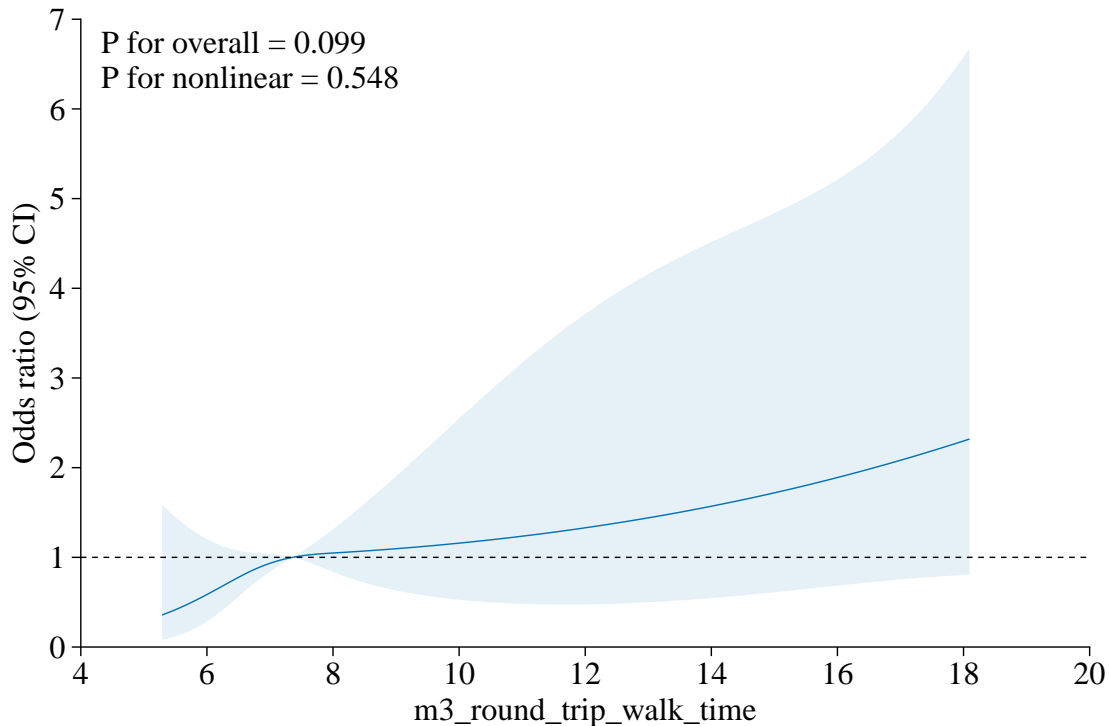

Supplement: Supplementary file 1 [file DataSheet1.zip › Supplement/RCS/m3_round_trip_walk_time.pdf]

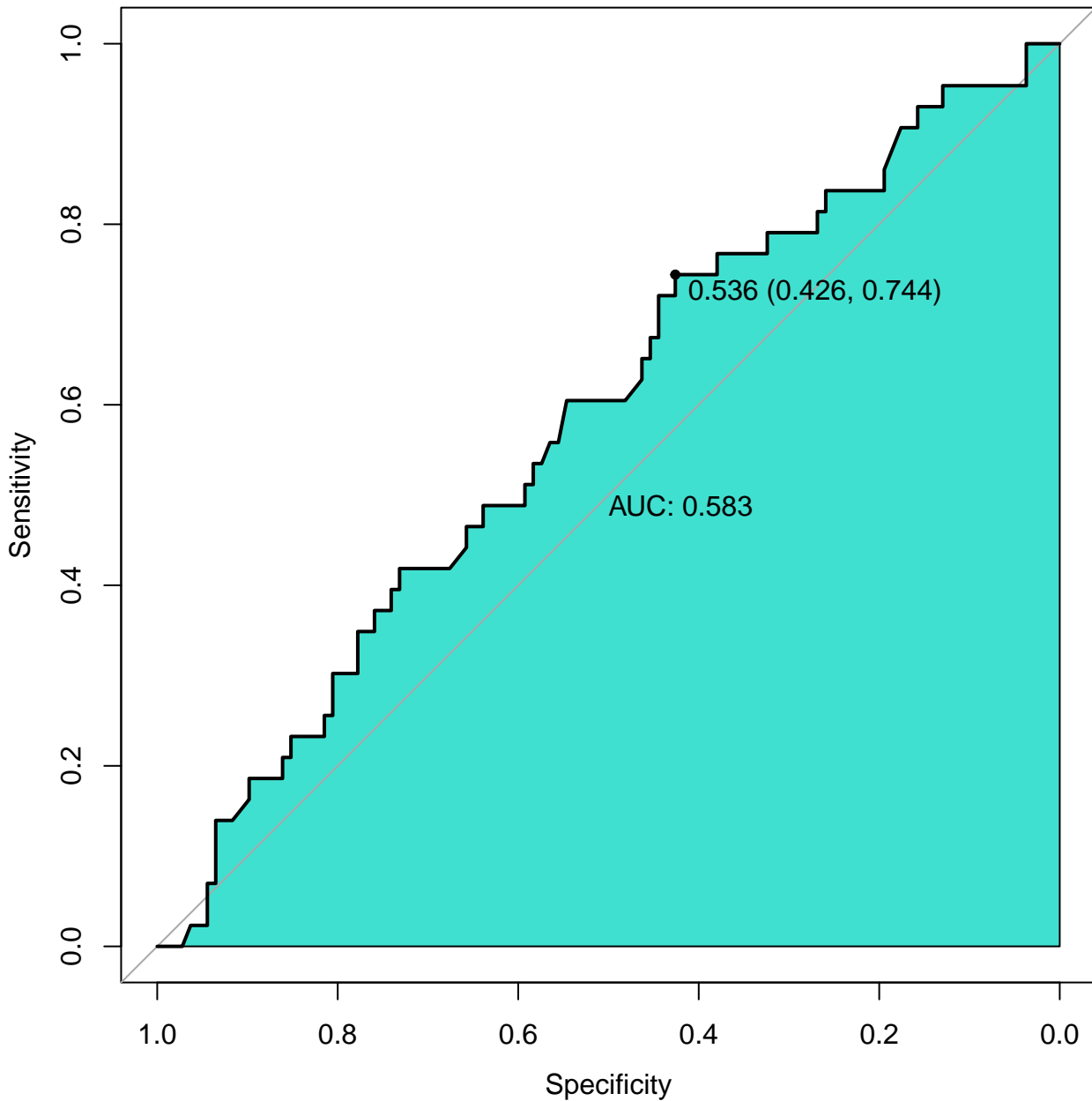

Supplement: Supplementary file 1 [file DataSheet1.zip › Supplement/ROC/trioxypurine.pdf]

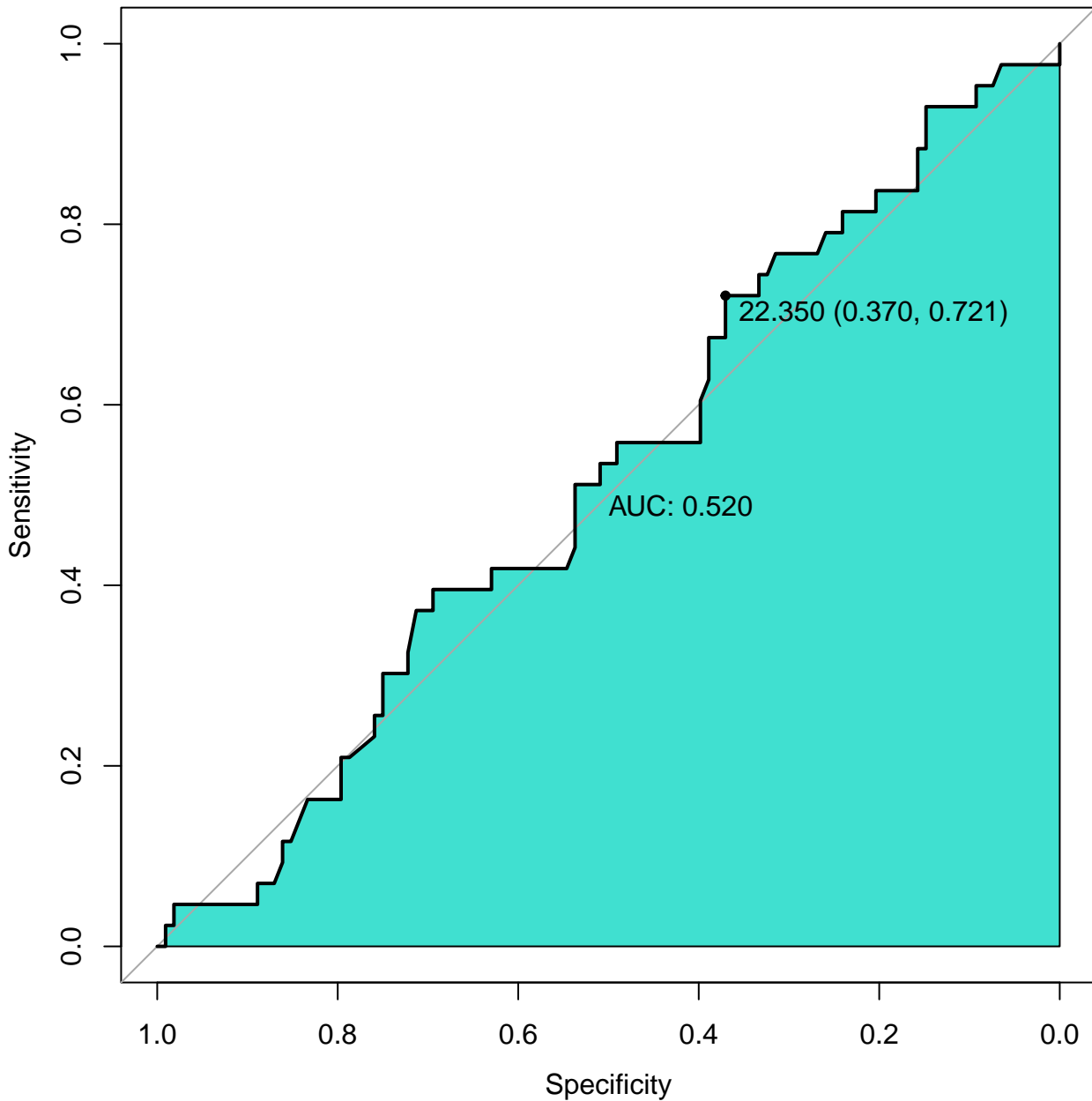

Supplement: Supplementary file 1 [file DataSheet1.zip › Supplement/ROC/vitD.pdf]

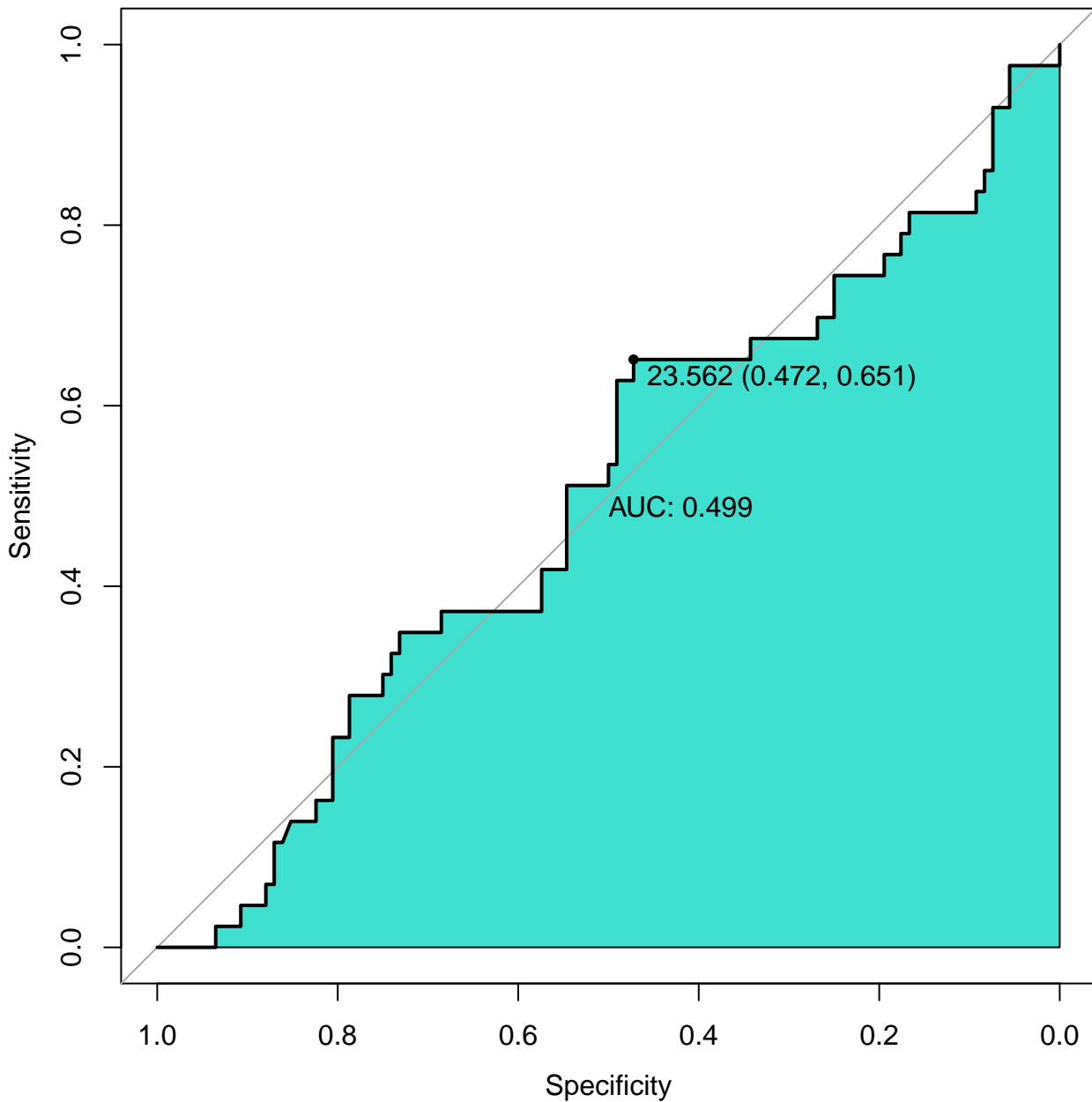

Supplement: Supplementary file 1 [file DataSheet1.zip › Supplement/ROC/BMI.pdf]

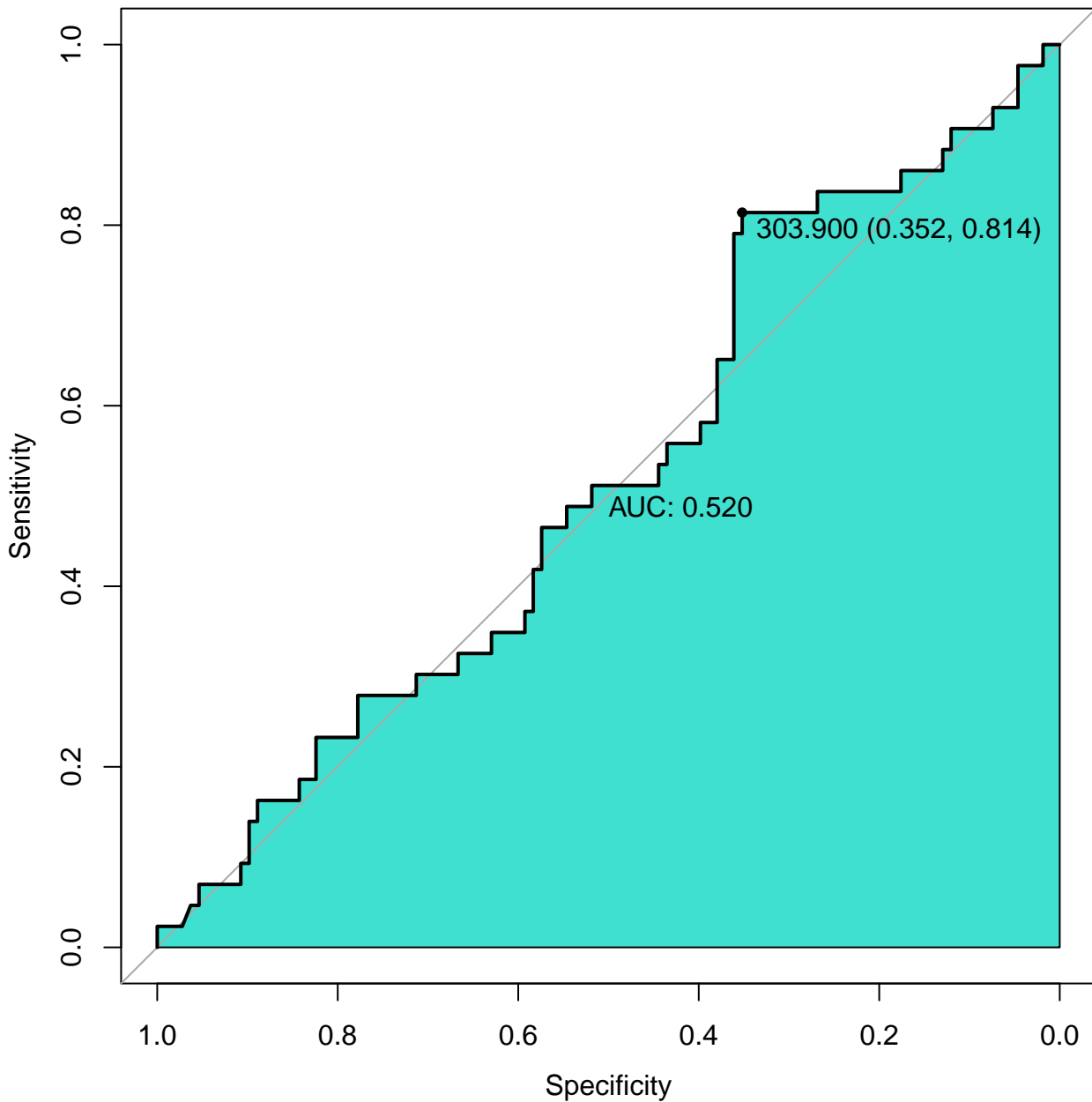

Supplement: Supplementary file 1 [file DataSheet1.zip › Supplement/ROC/parathyrin.pdf]

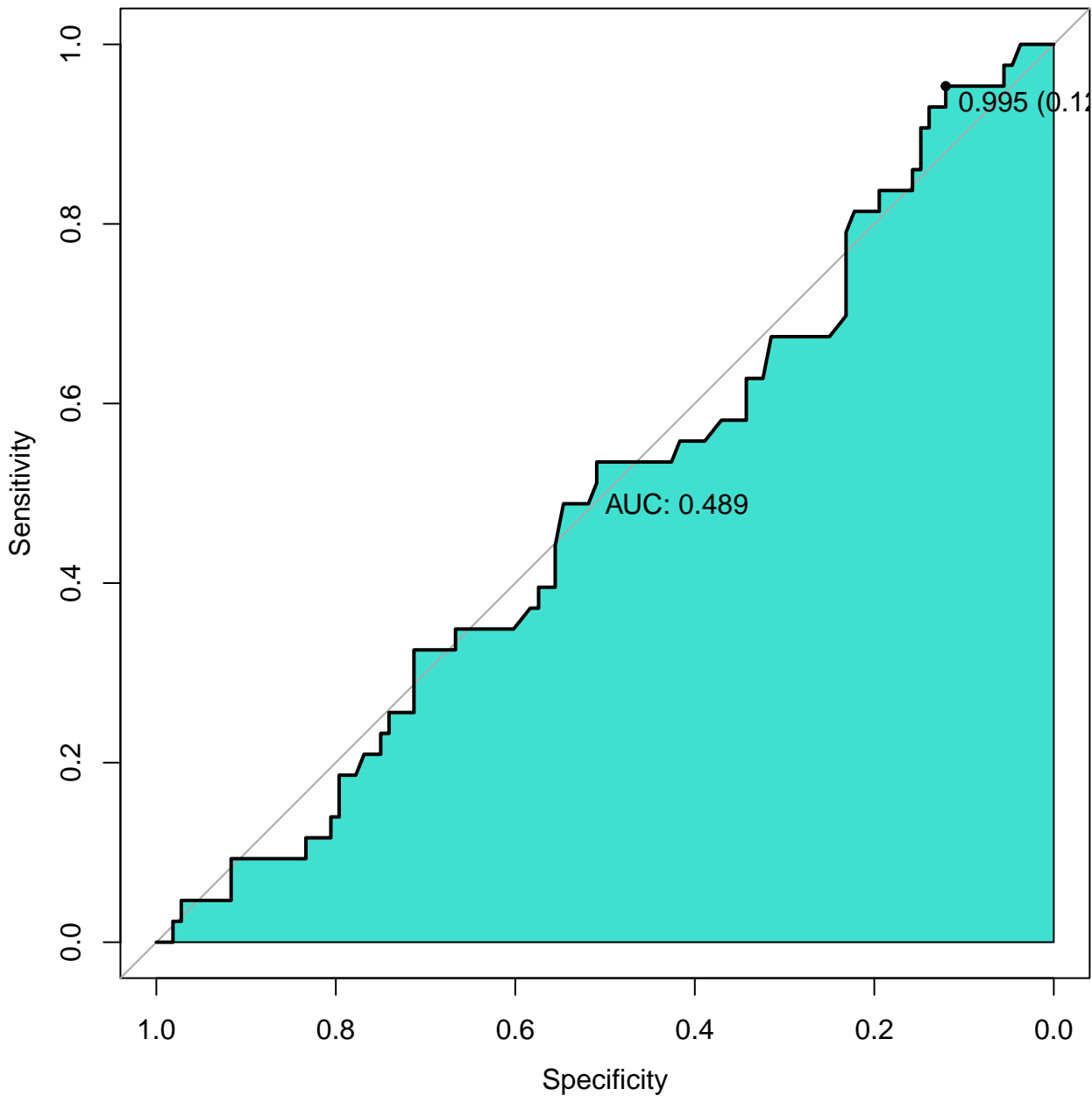

Supplement: Supplementary file 1 [file DataSheet1.zip › Supplement/ROC/triglyceride.pdf]

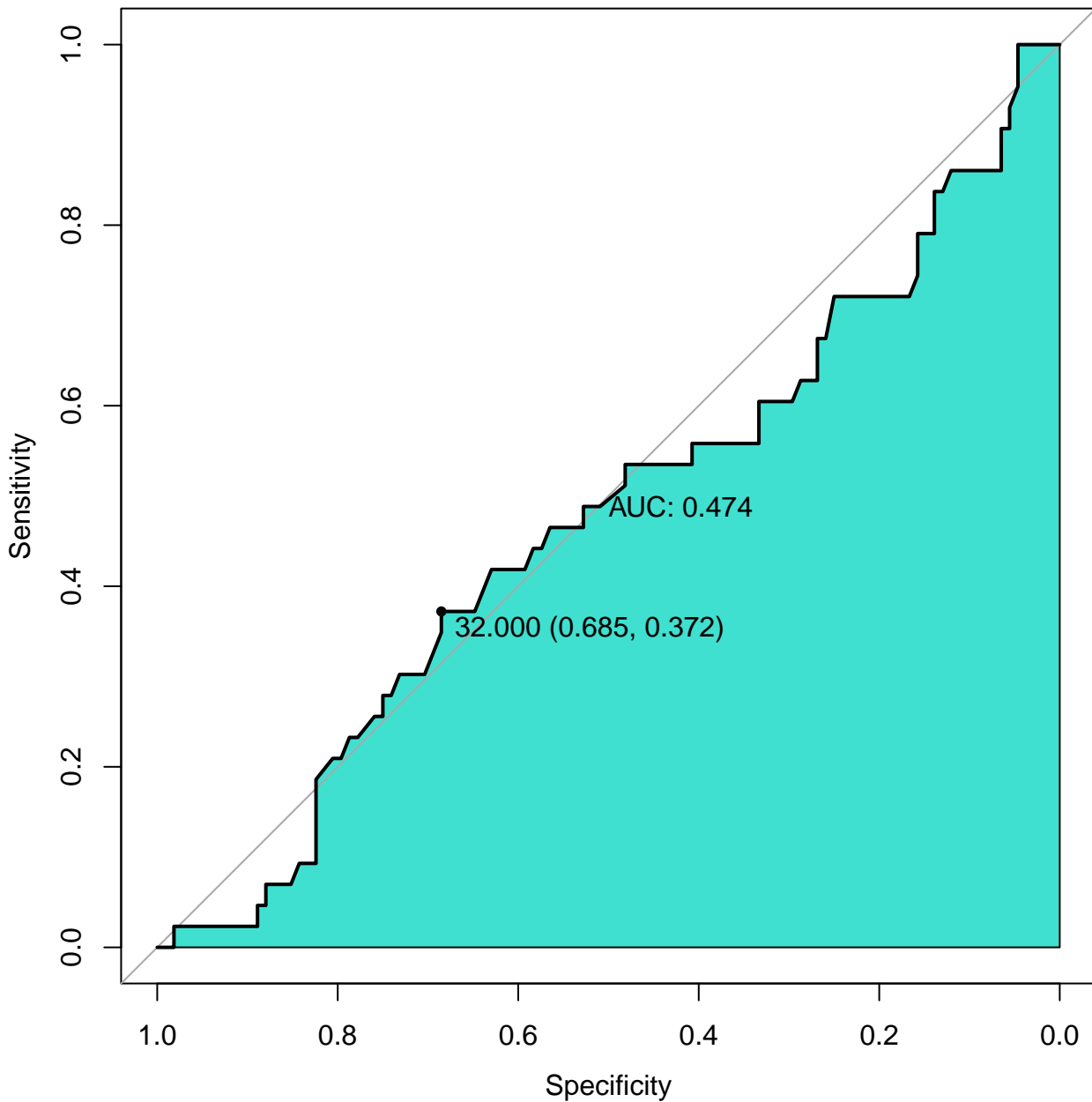

Supplement: Supplementary file 1 [file DataSheet1.zip › Supplement/ROC/urea_before_dialysis.pdf]

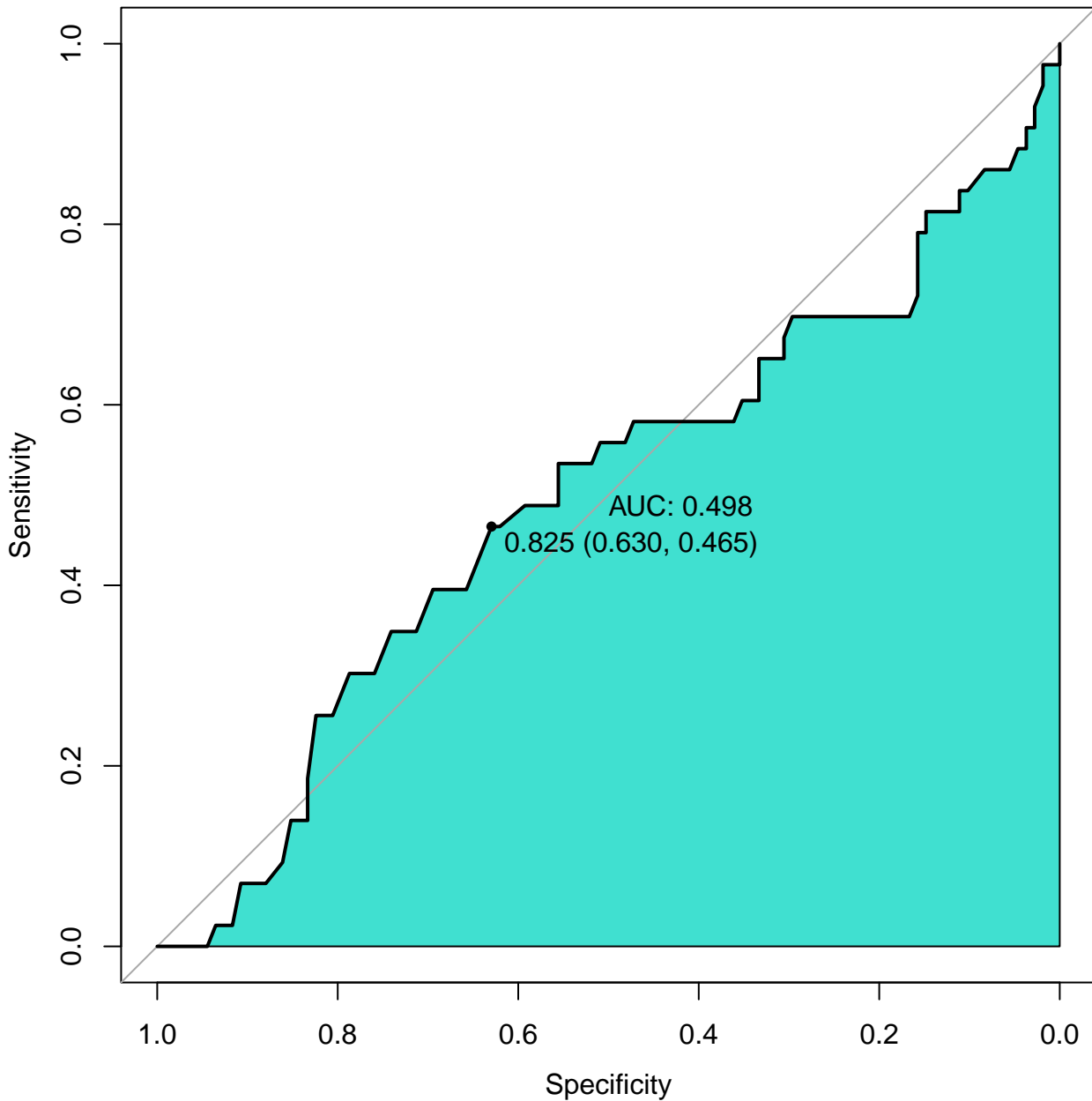

Supplement: Supplementary file 1 [file DataSheet1.zip › Supplement/ROC/HDL.pdf]

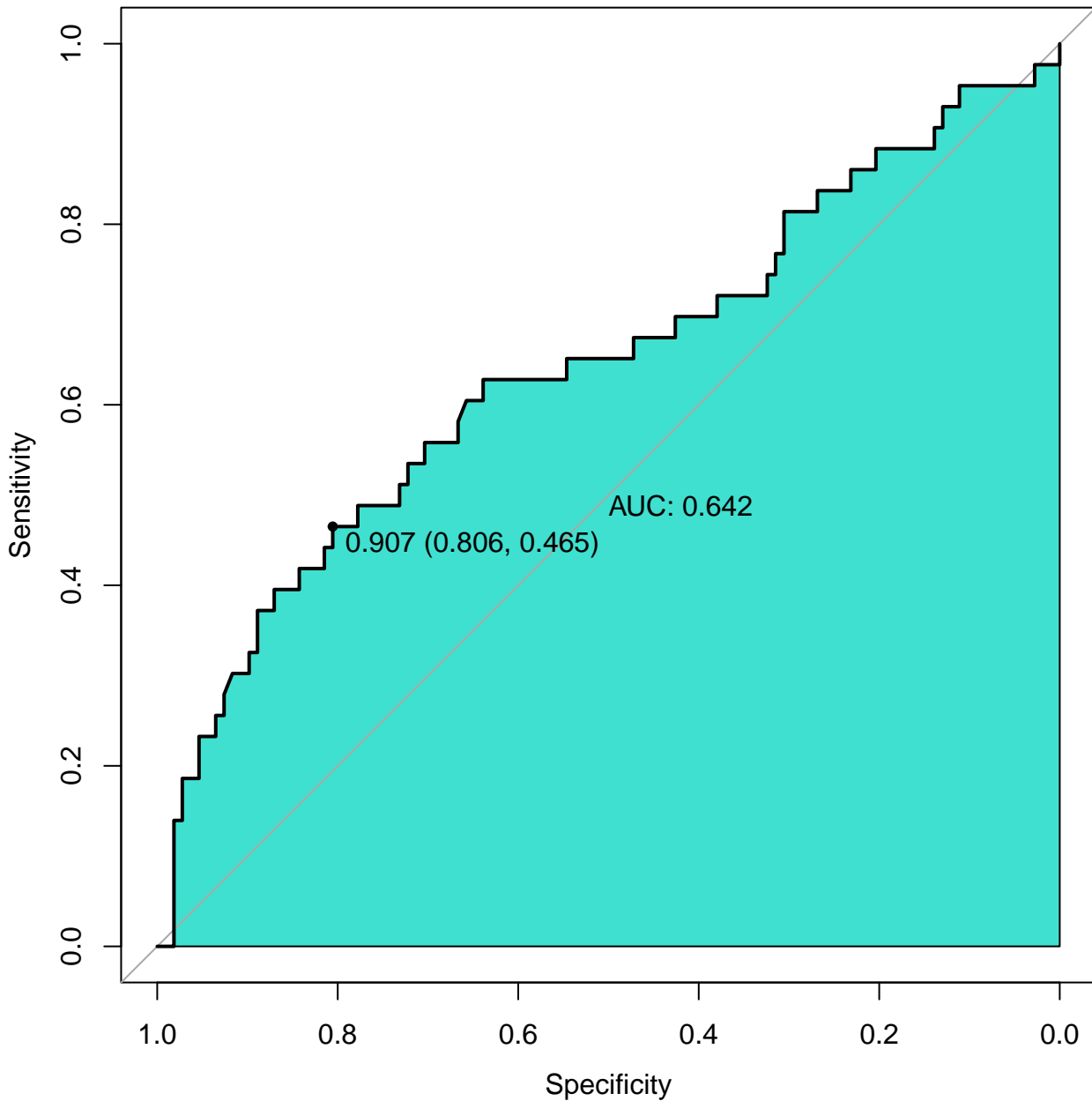

Supplement: Supplementary file 1 [file DataSheet1.zip › Supplement/ROC/average_gait_speed.pdf]

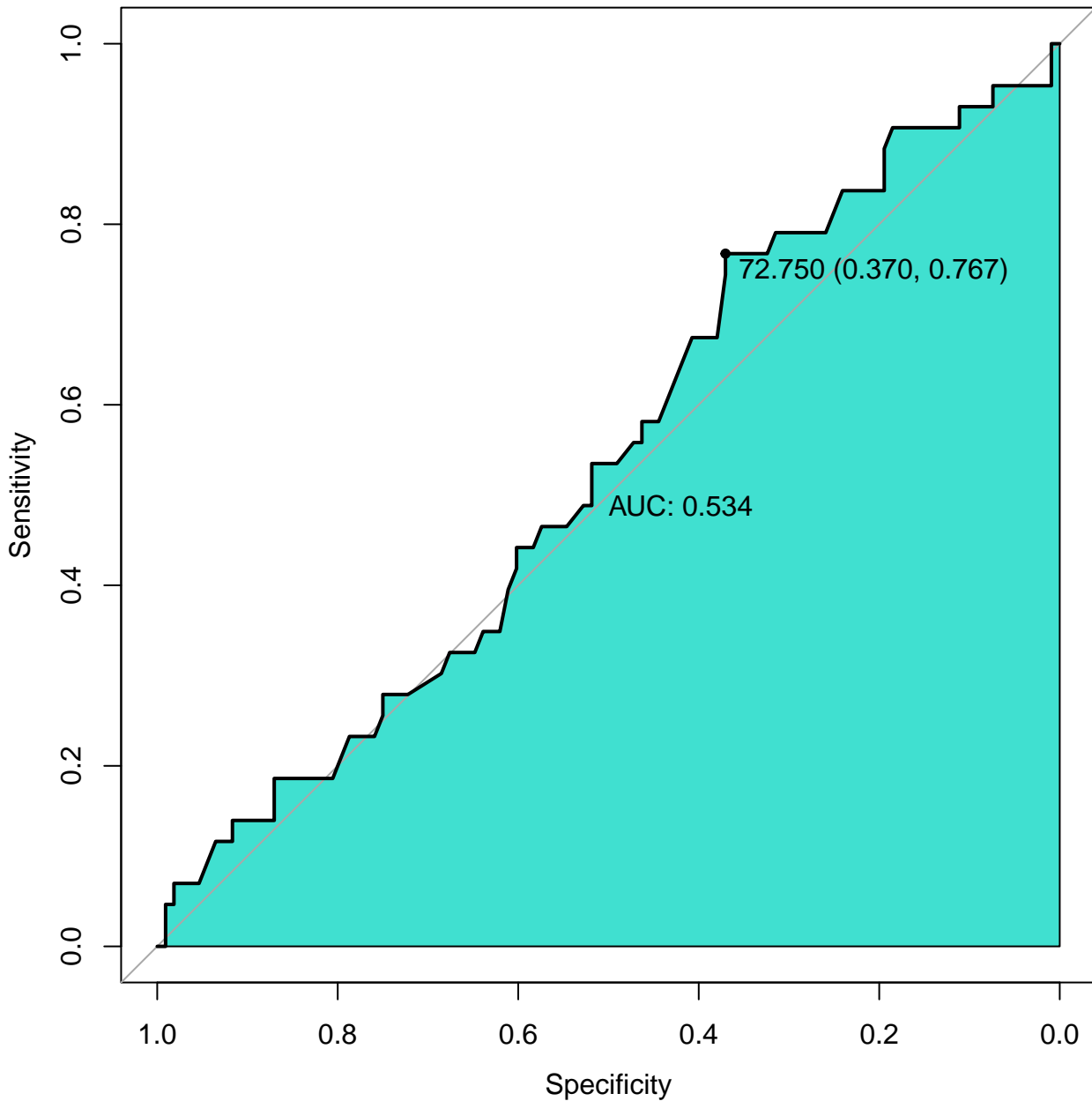

Supplement: Supplementary file 1 [file DataSheet1.zip › Supplement/ROC/total_protein.pdf]

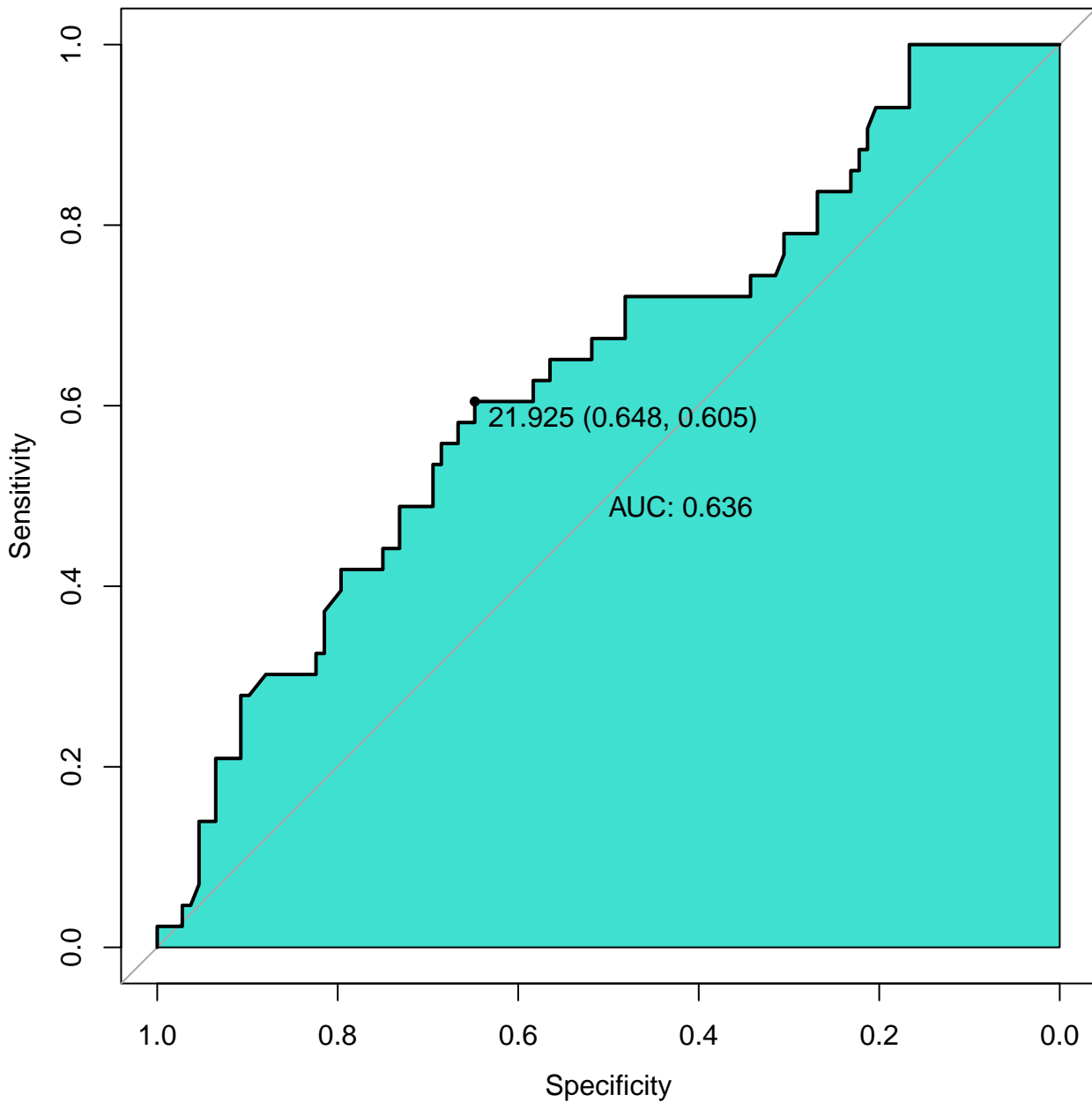

Supplement: Supplementary file 1 [file DataSheet1.zip › Supplement/ROC/average_hand_grip.pdf]

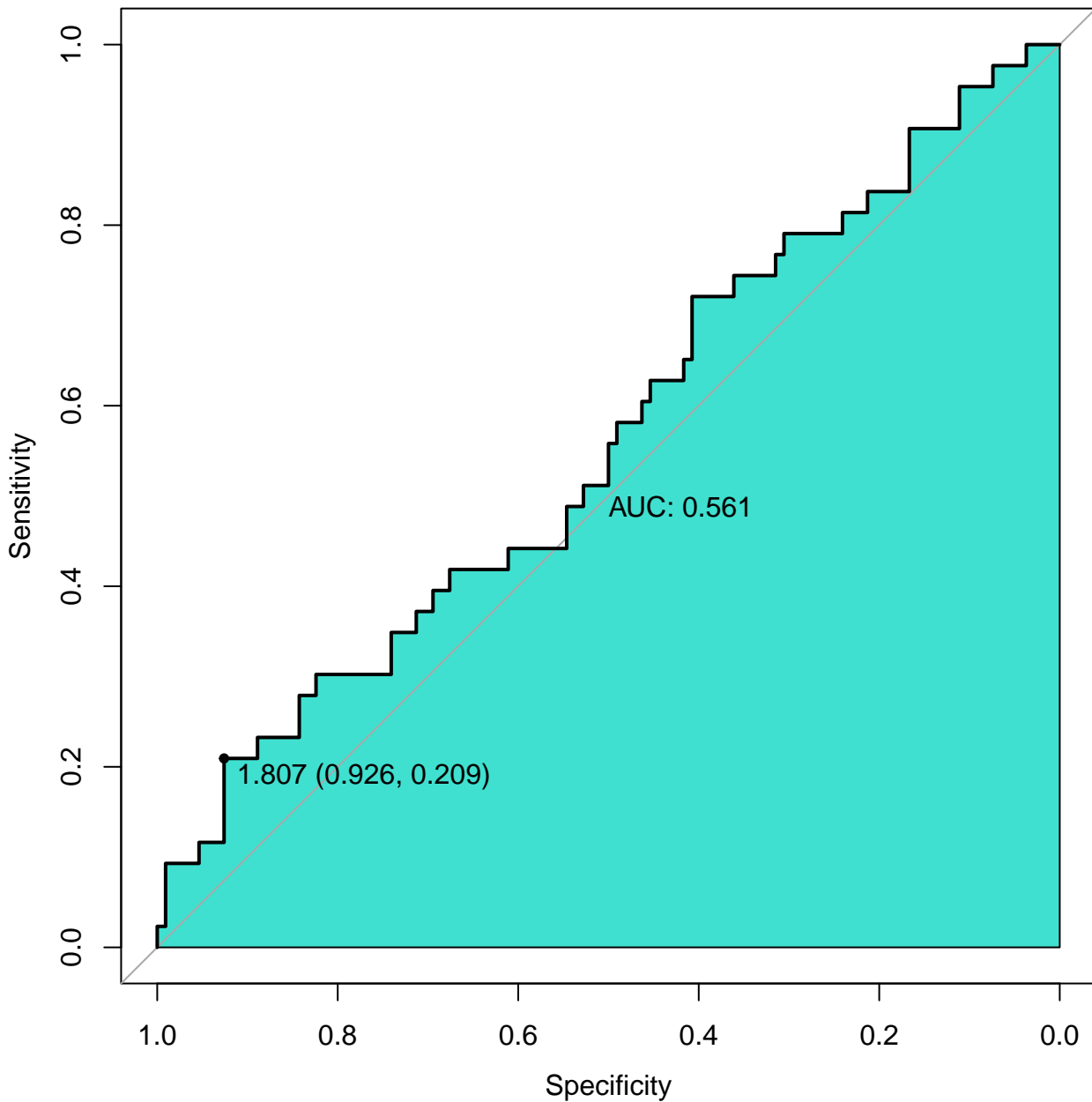

Supplement: Supplementary file 1 [file DataSheet1.zip › Supplement/ROC/Kt_V.pdf]

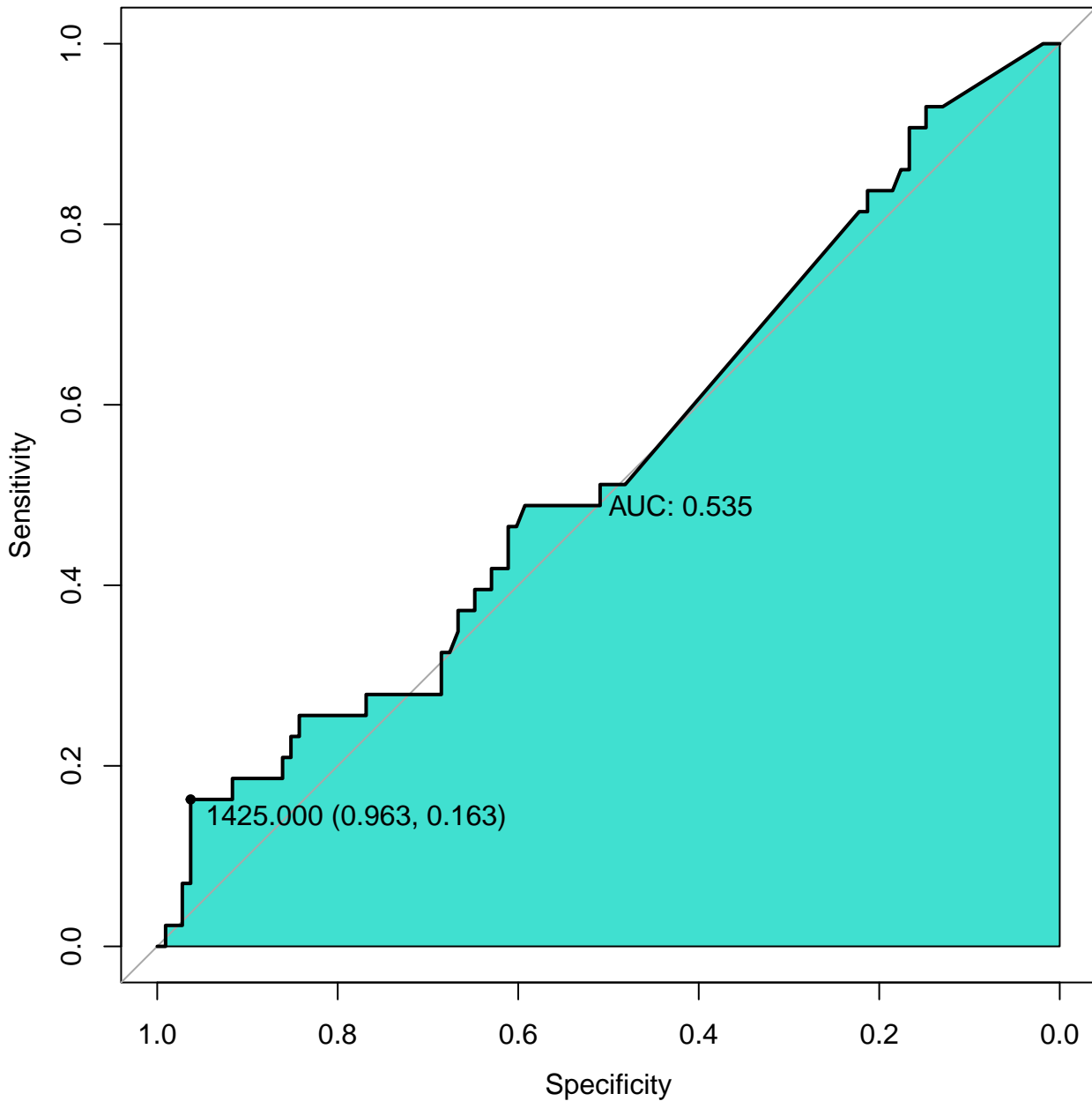

Supplement: Supplementary file 1 [file DataSheet1.zip › Supplement/ROC/ferritin.pdf]

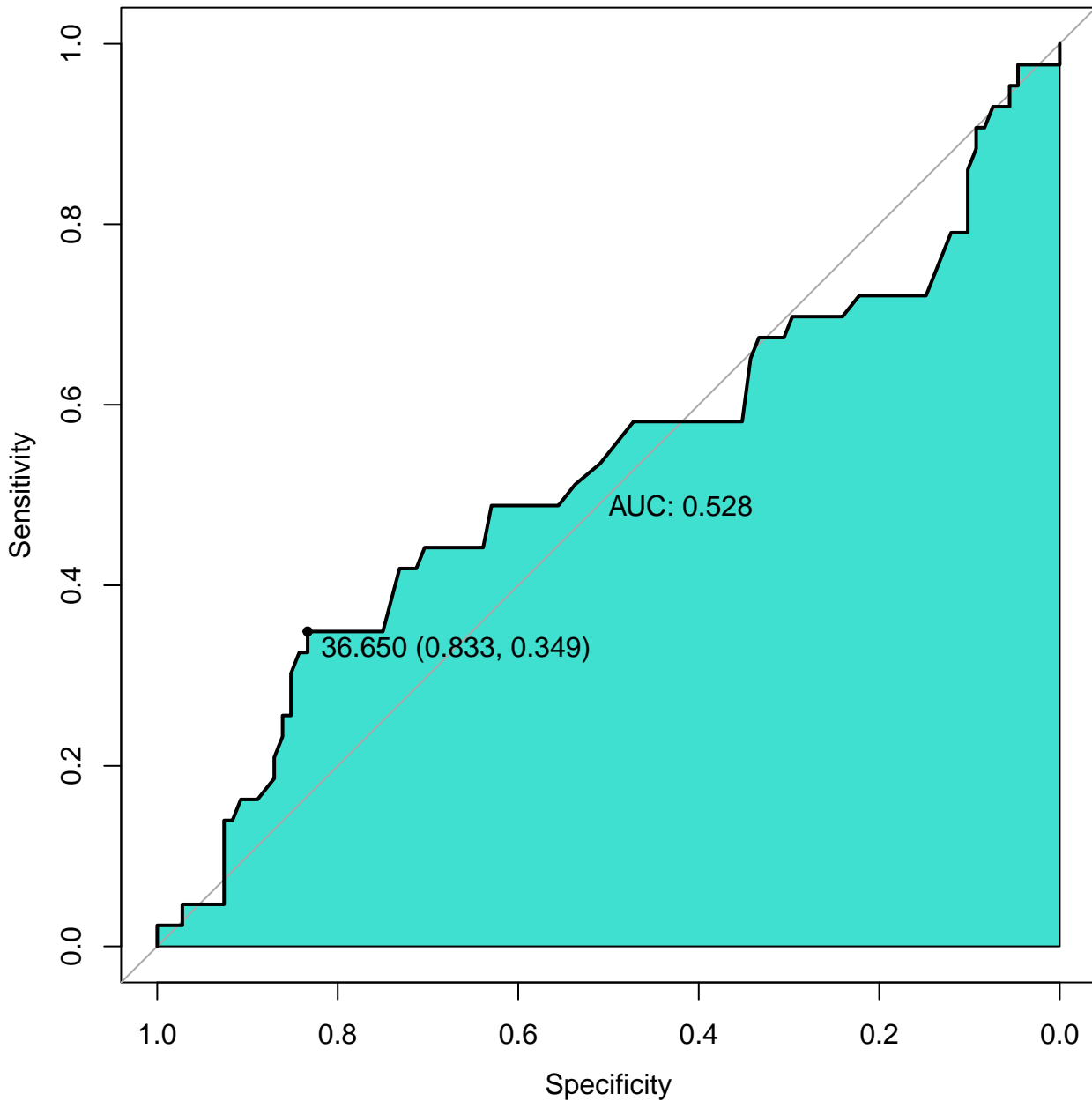

Supplement: Supplementary file 1 [file DataSheet1.zip › Supplement/ROC/albumin.pdf]

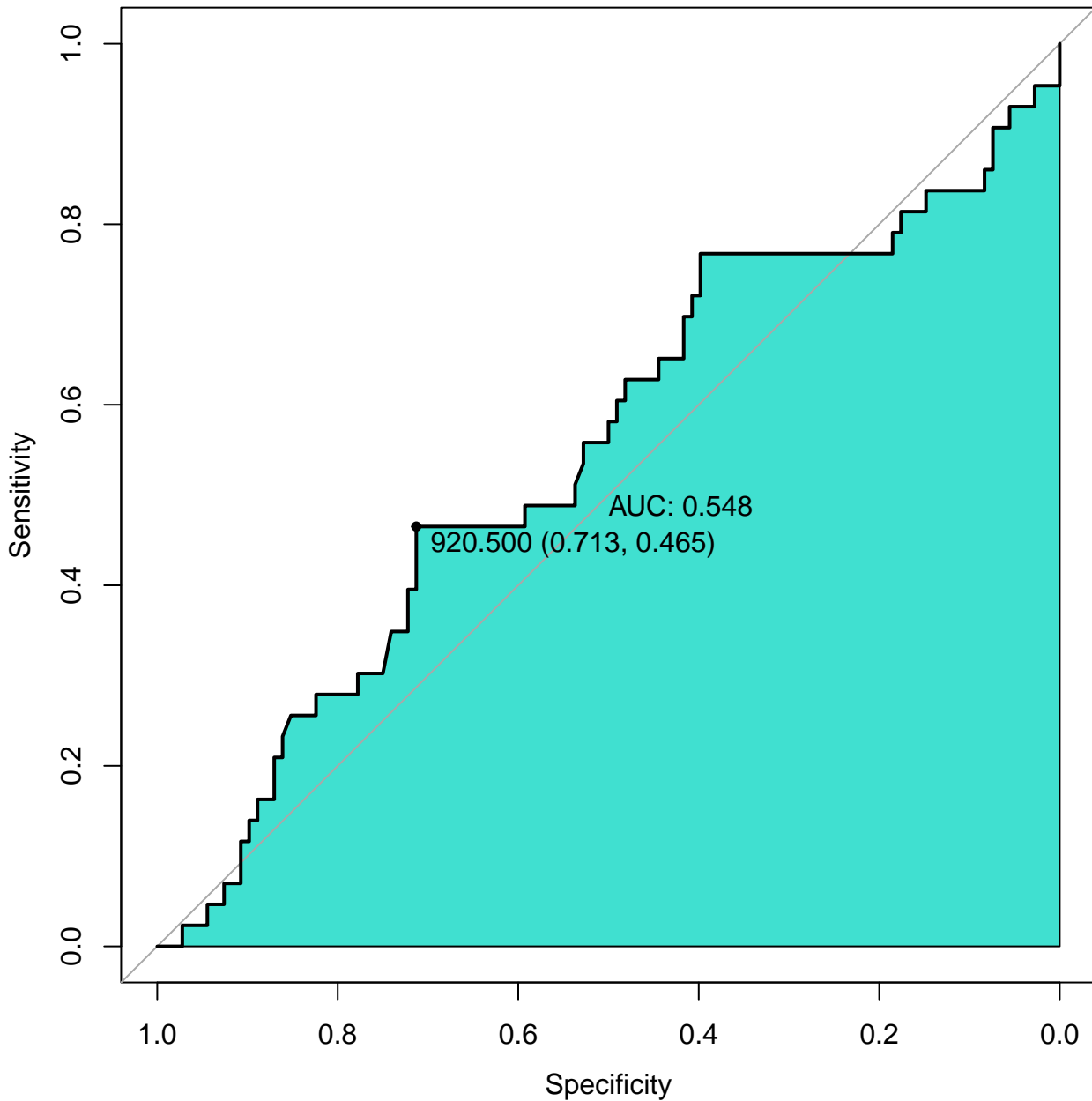

Supplement: Supplementary file 1 [file DataSheet1.zip › Supplement/ROC/creatinine_before_dialysis.pdf]

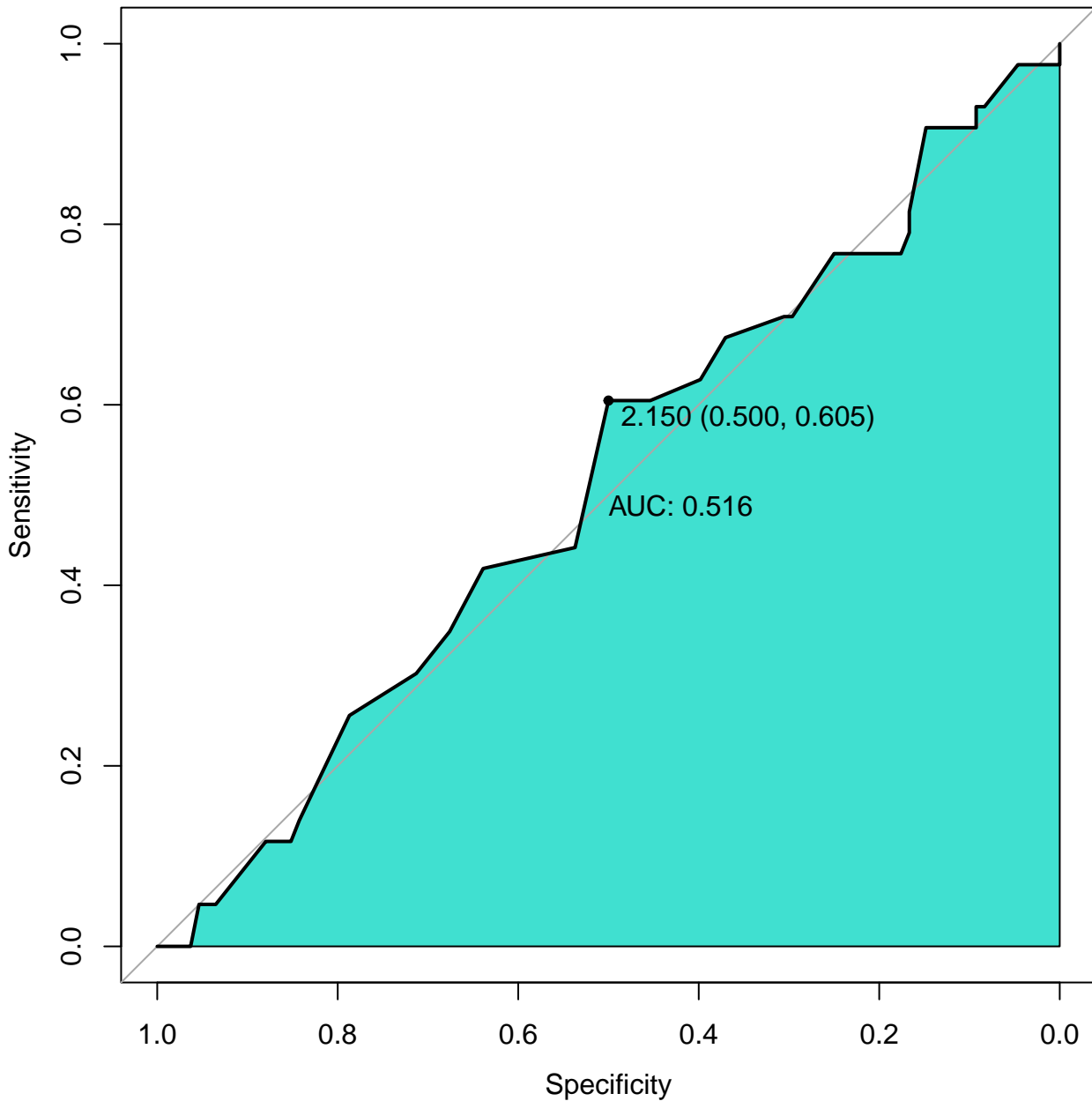

Supplement: Supplementary file 1 [file DataSheet1.zip › Supplement/ROC/LDL.pdf]

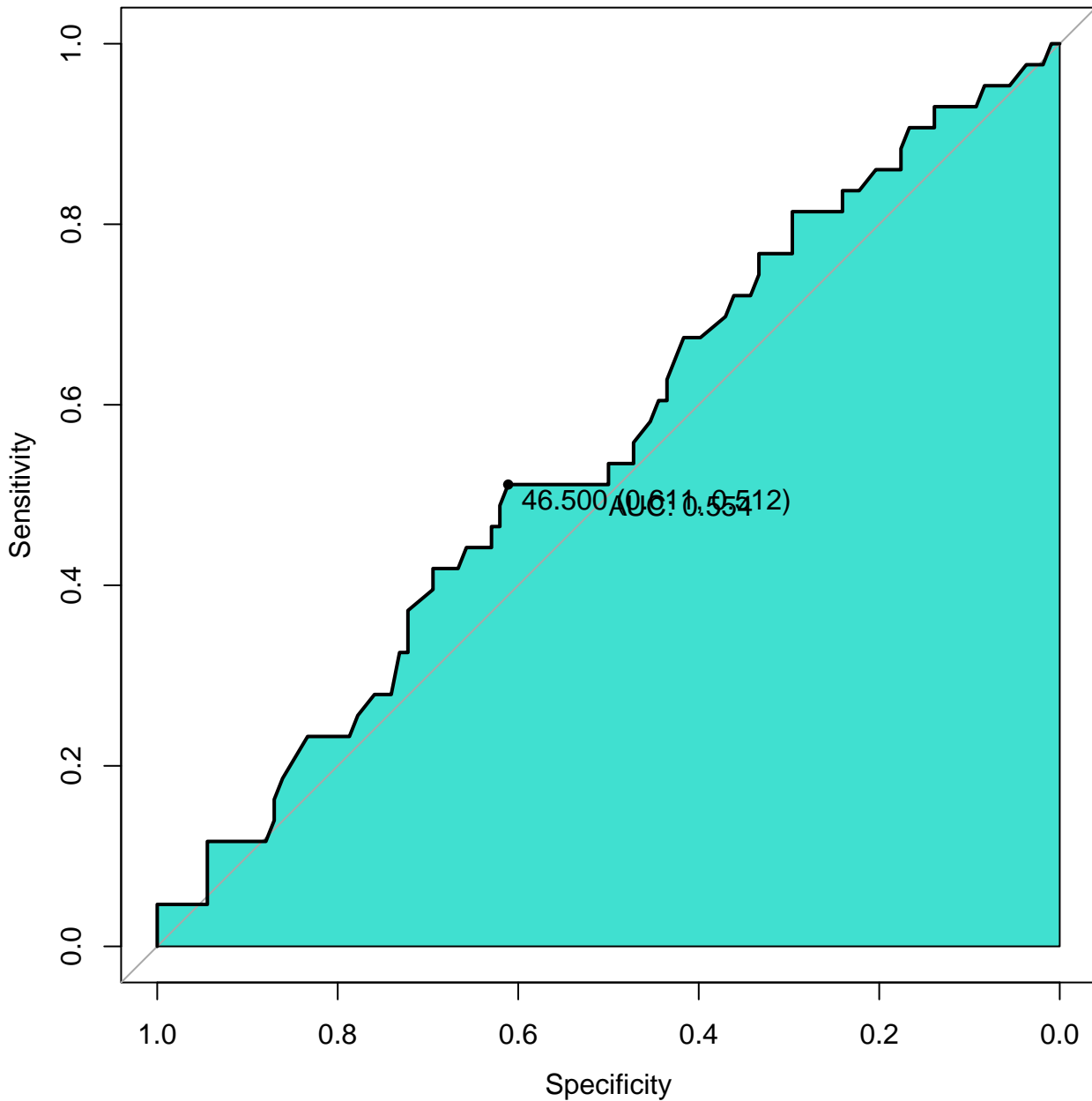

Supplement: Supplementary file 1 [file DataSheet1.zip › Supplement/ROC/dialysis_time.pdf]

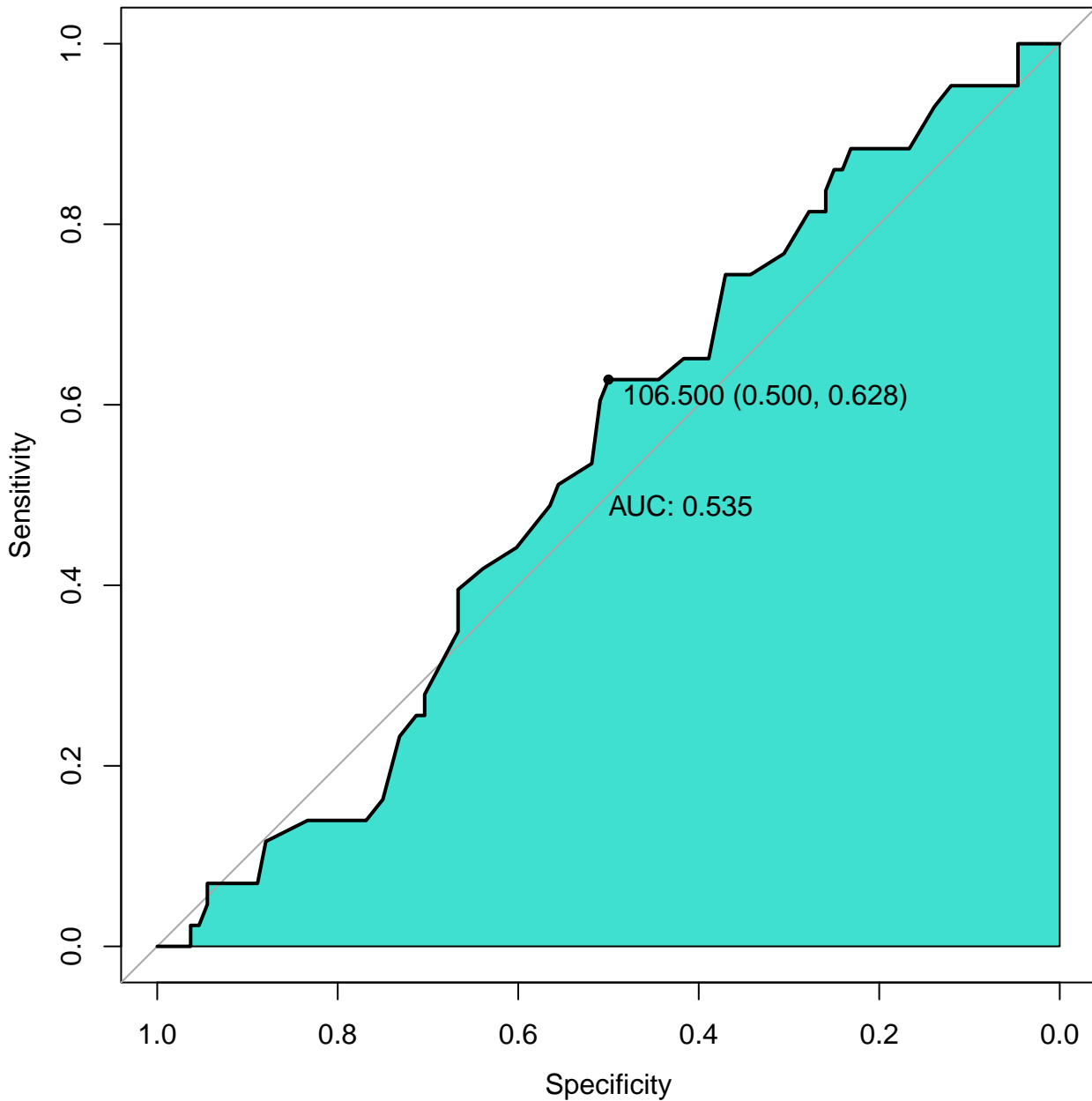

Supplement: Supplementary file 1 [file DataSheet1.zip › Supplement/ROC/HB.pdf]

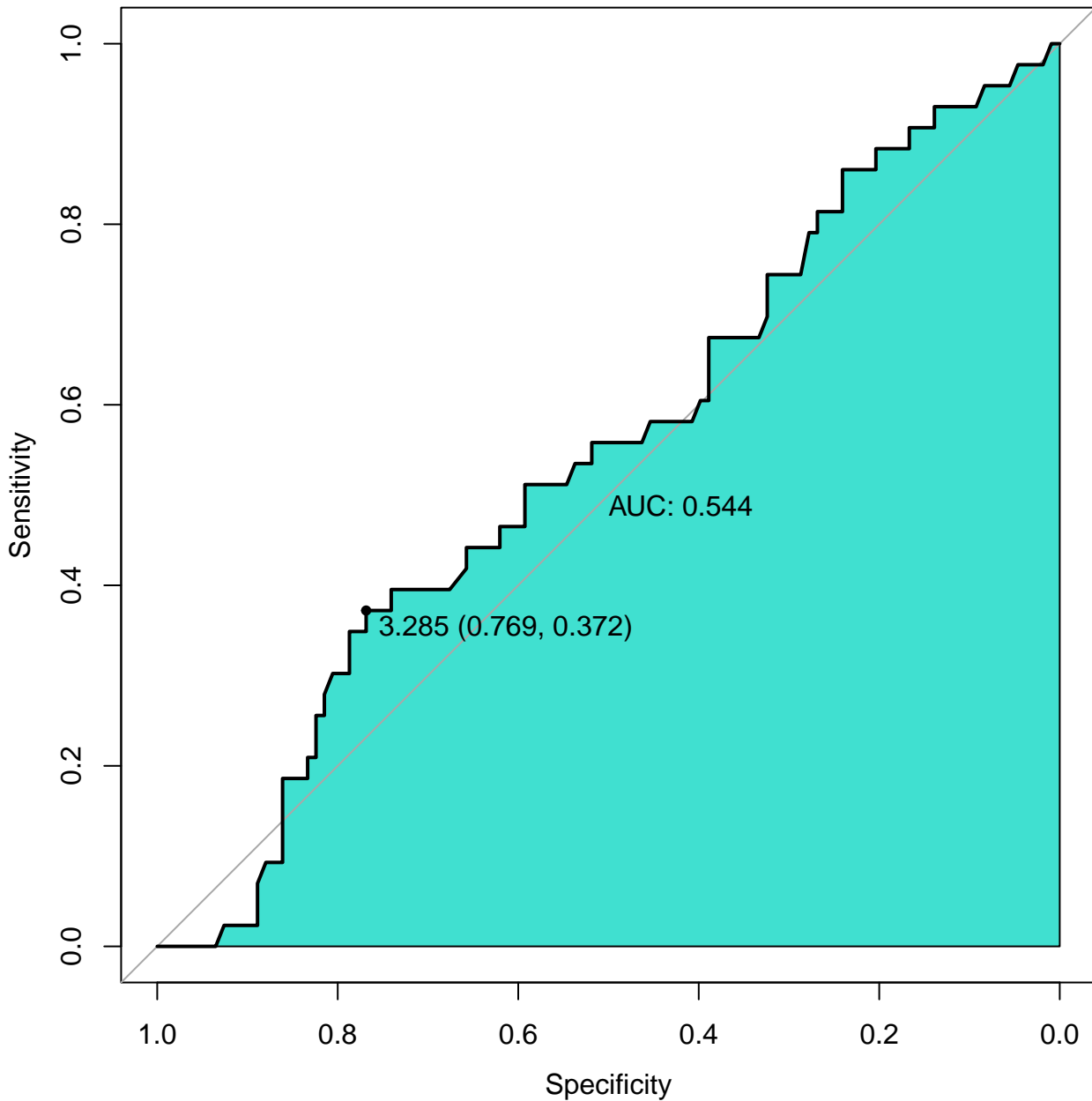

Supplement: Supplementary file 1 [file DataSheet1.zip › Supplement/ROC/cholesterol.pdf]

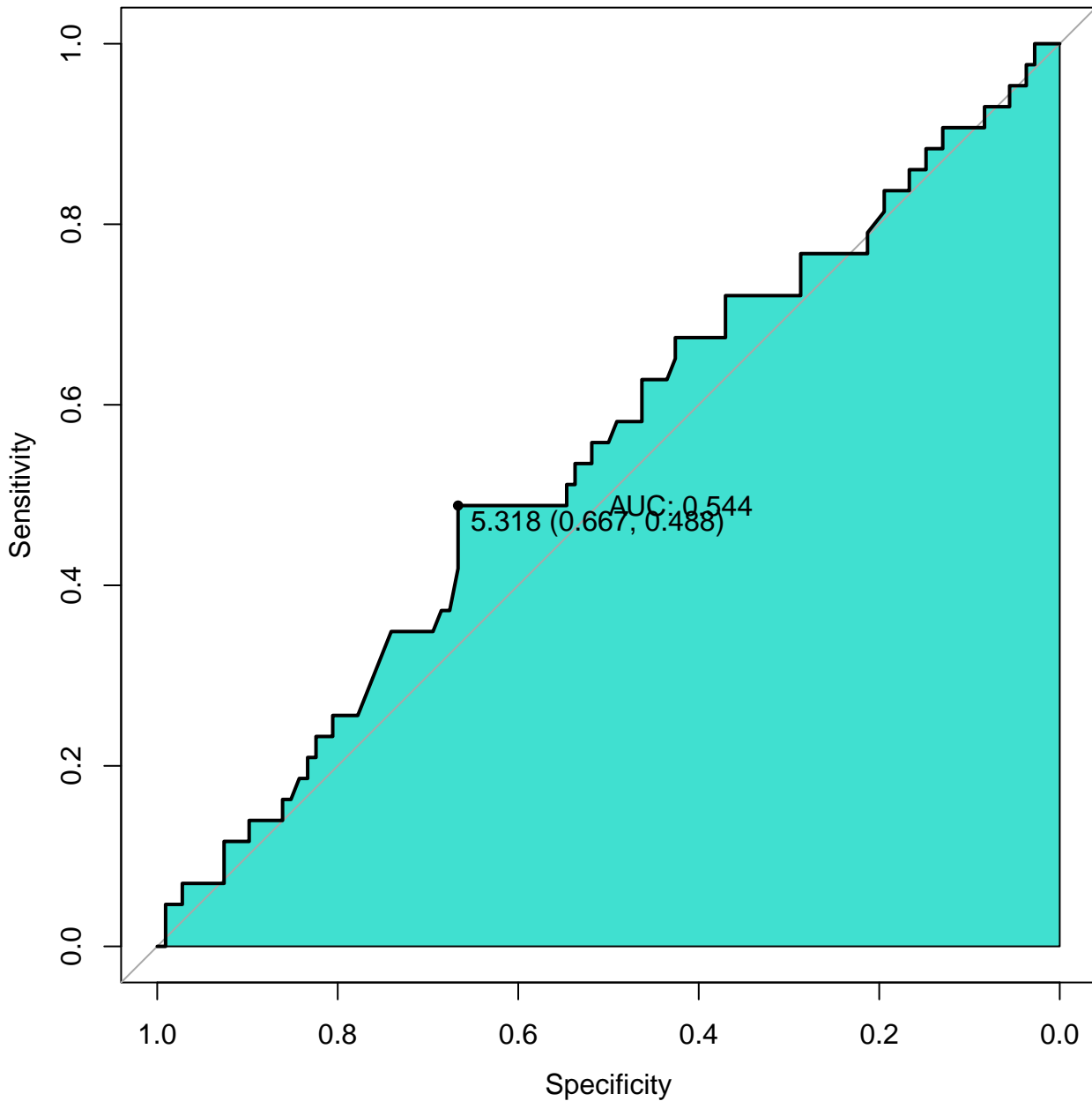

Supplement: Supplementary file 1 [file DataSheet1.zip › Supplement/ROC/pct_of_ultrafiltration.pdf]

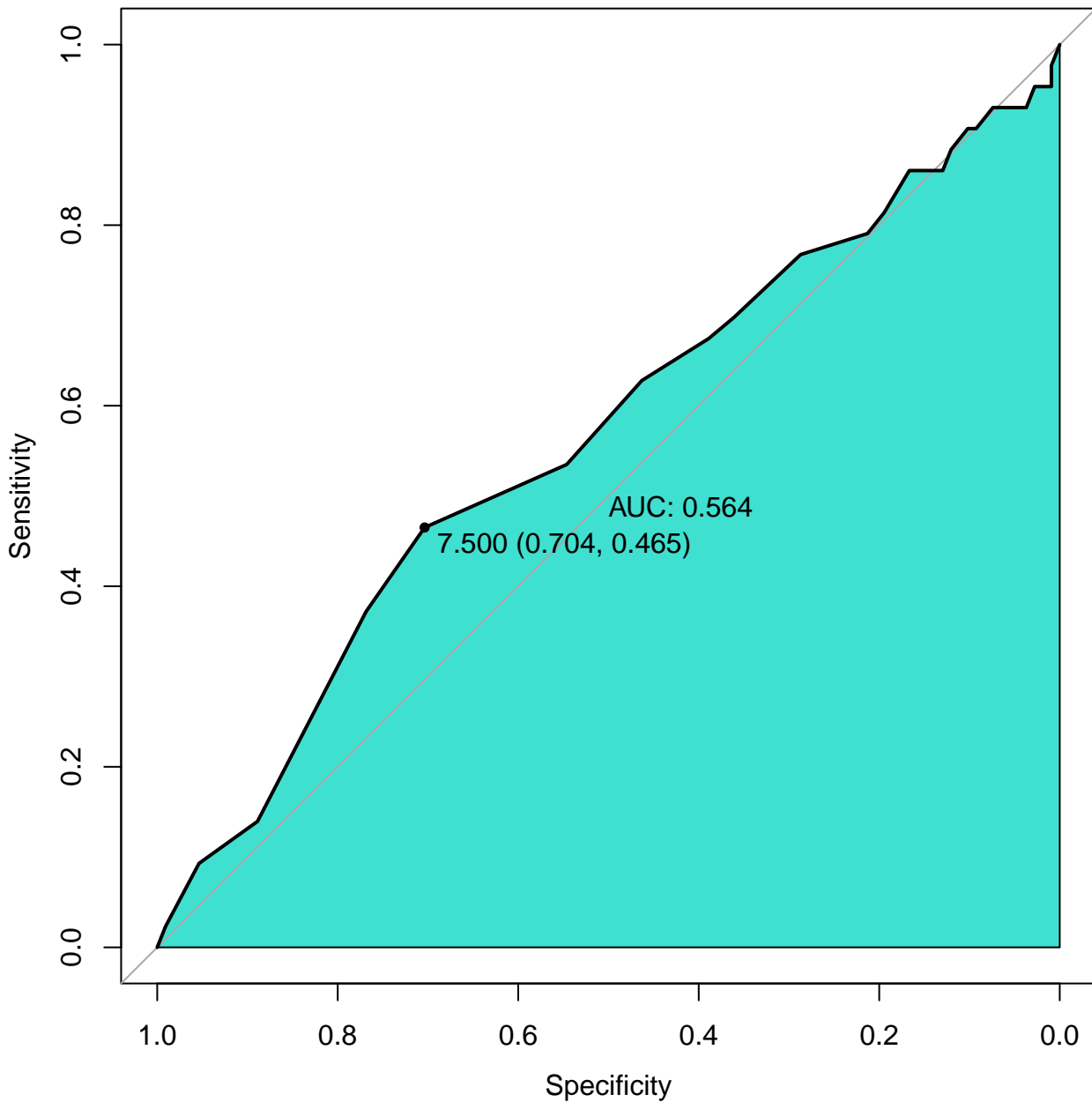

Supplement: Supplementary file 1 [file DataSheet1.zip › Supplement/ROC/ALT.pdf]

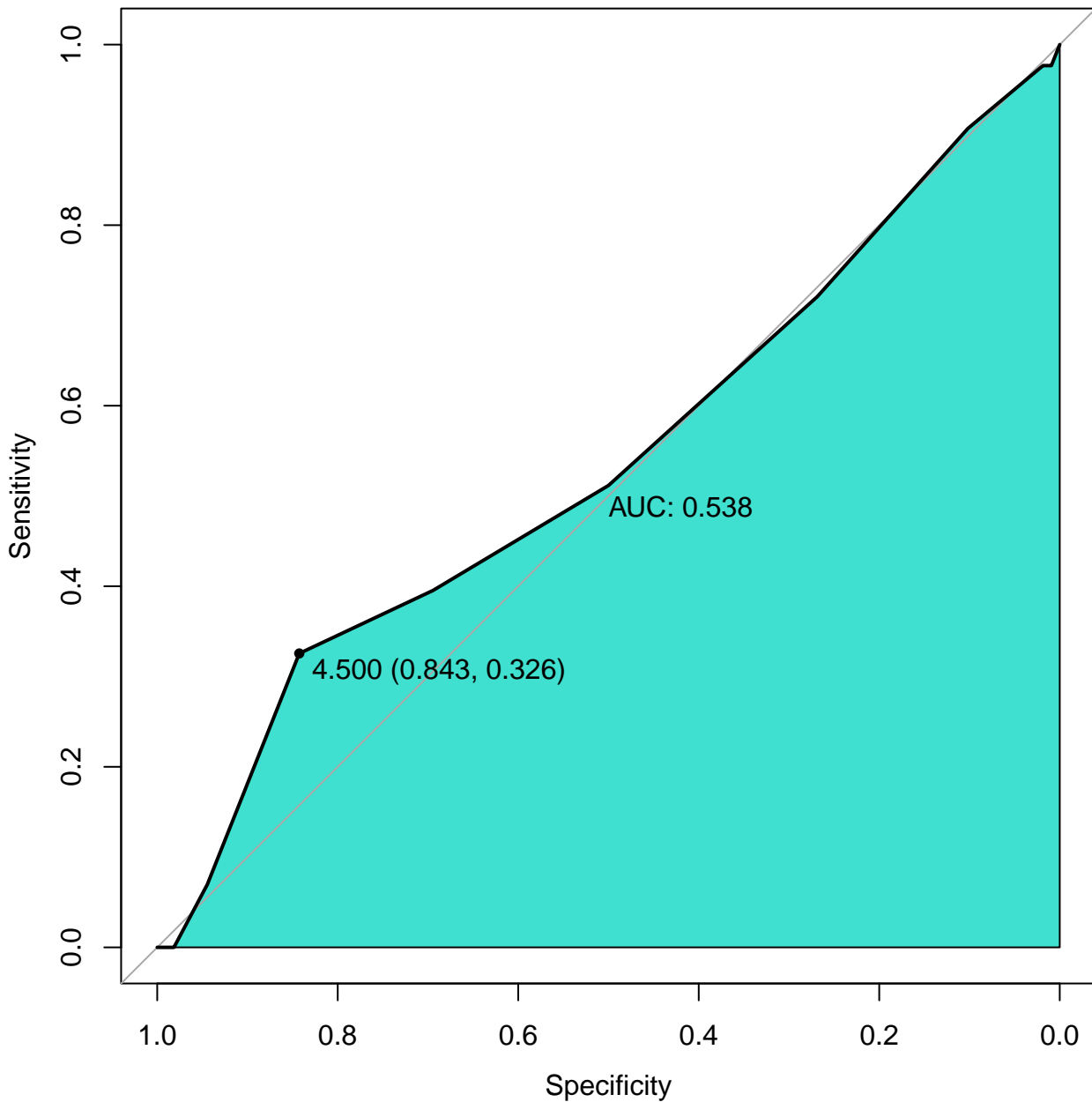

Supplement: Supplementary file 1 [file DataSheet1.zip › Supplement/ROC/sleep_time.pdf]

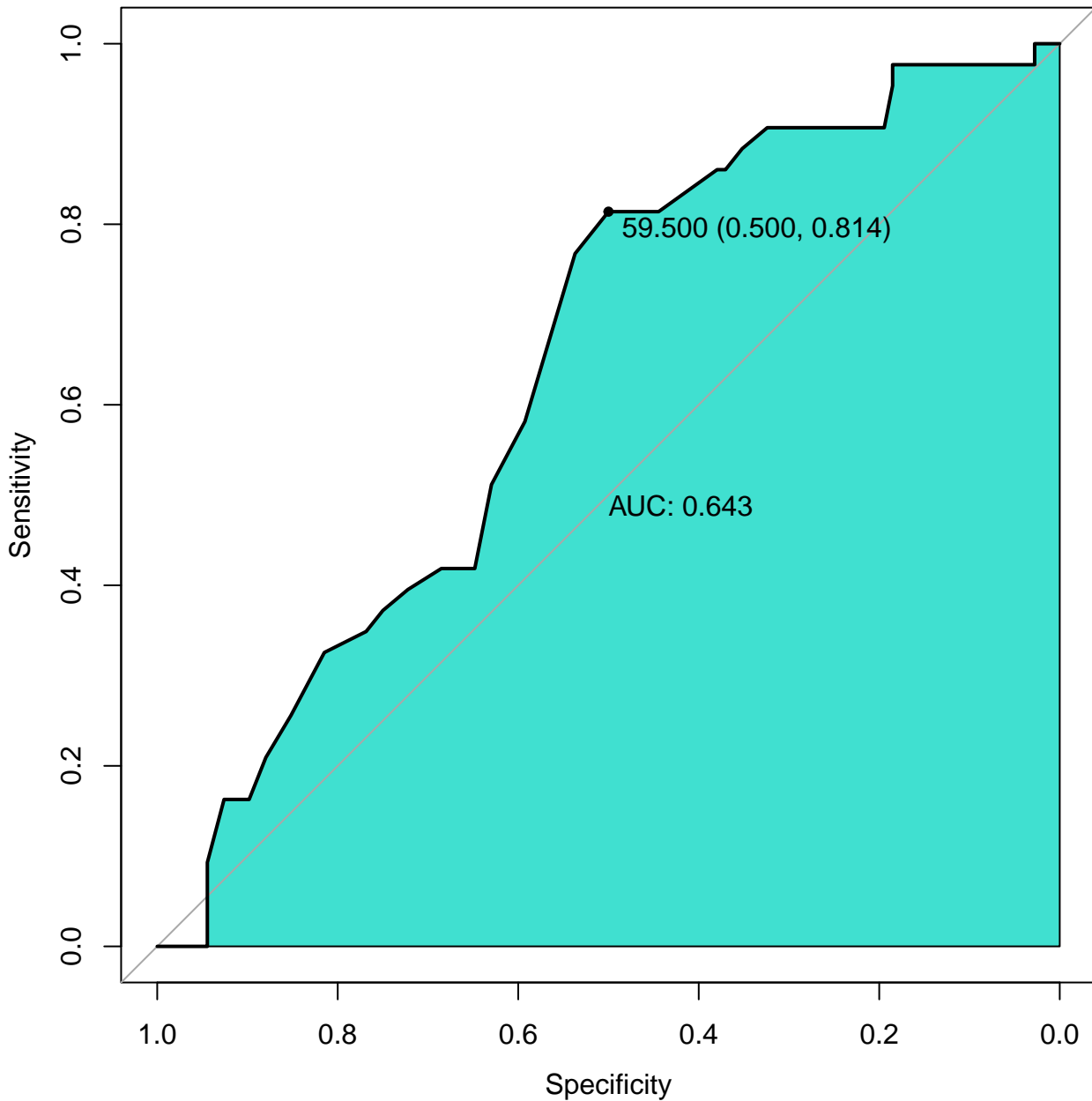

Supplement: Supplementary file 1 [file DataSheet1.zip › Supplement/ROC/age.pdf]

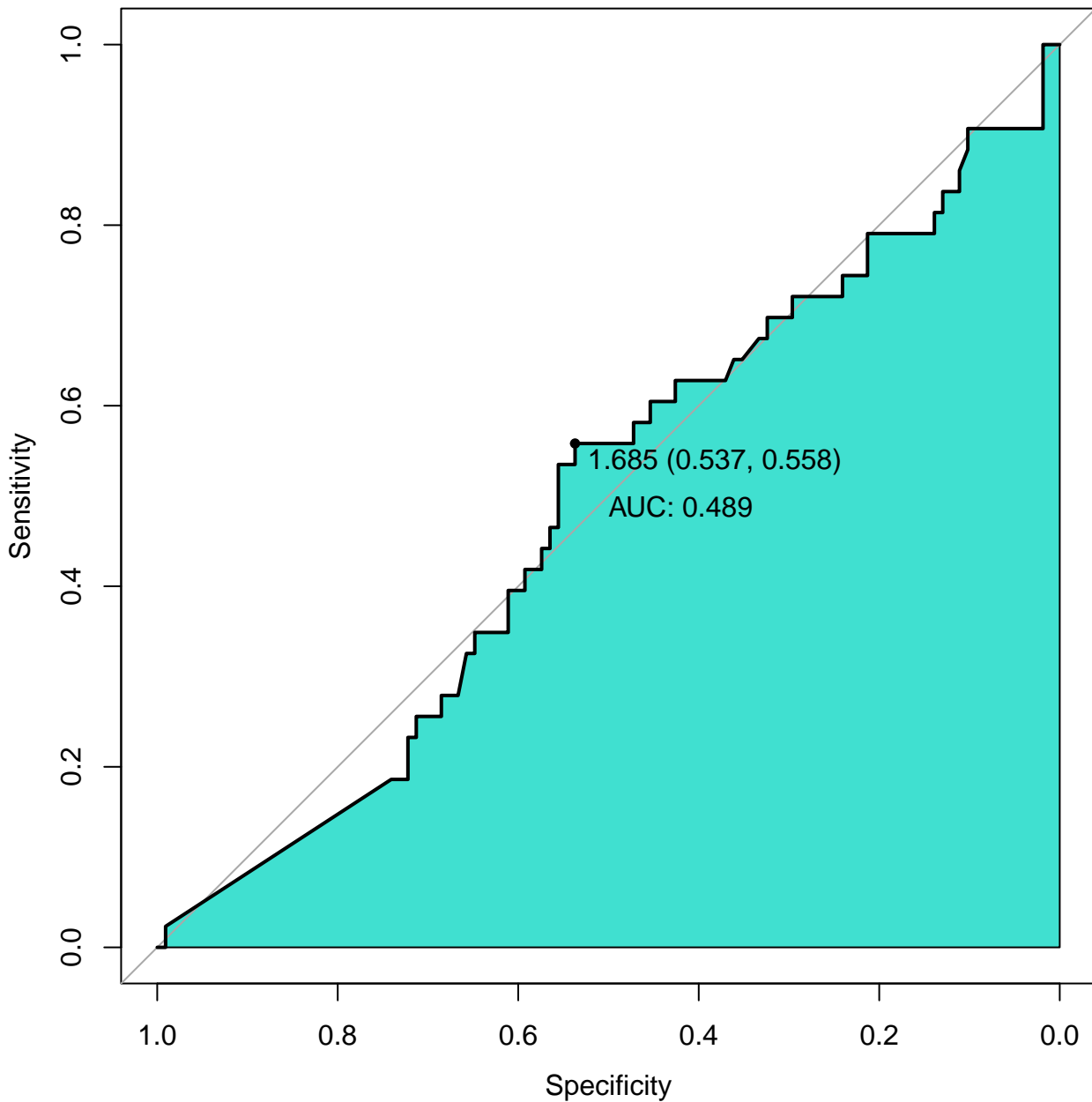

Supplement: Supplementary file 1 [file DataSheet1.zip › Supplement/ROC/CRP.pdf]

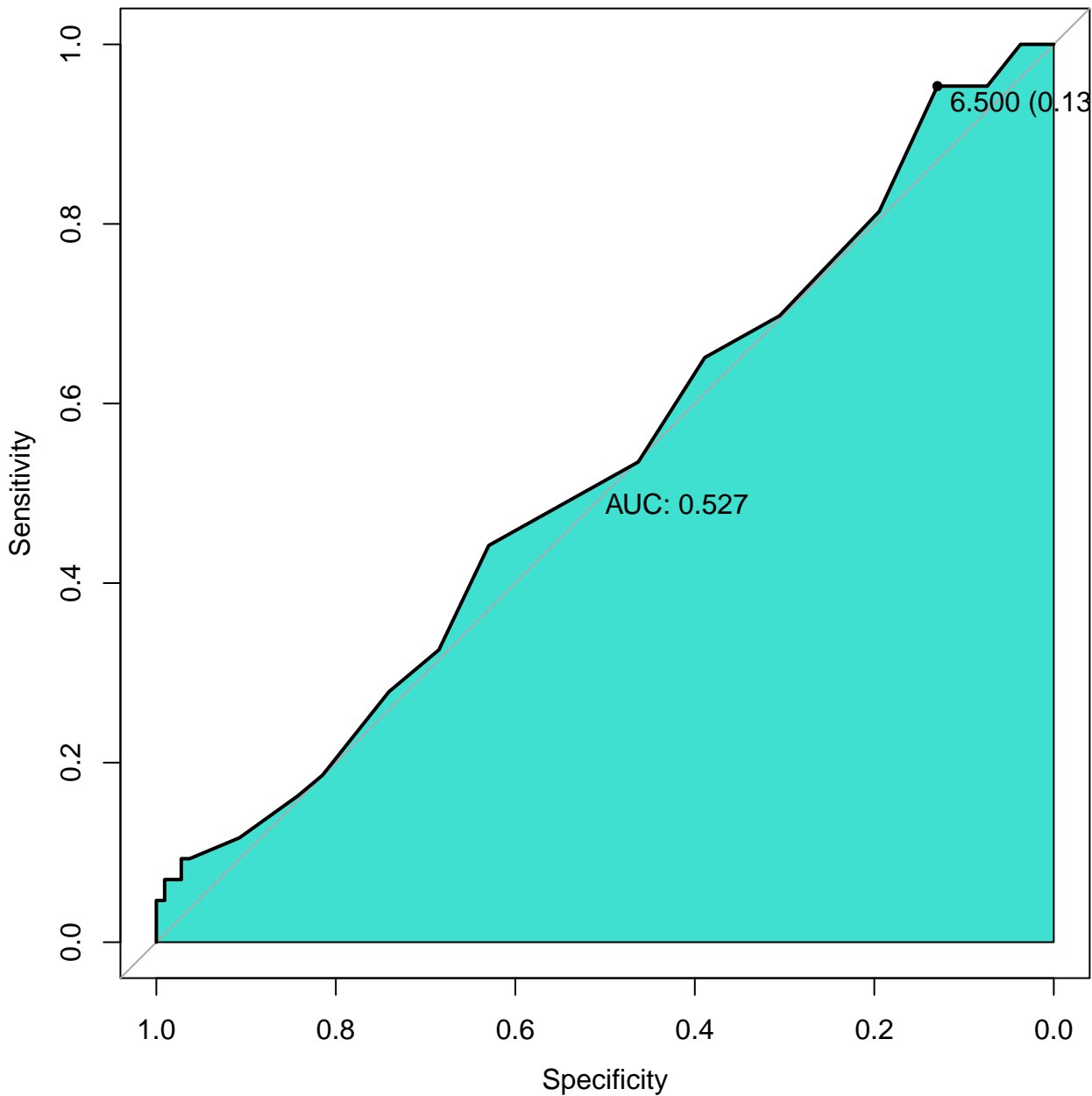

Supplement: Supplementary file 1 [file DataSheet1.zip › Supplement/ROC/AST.pdf]

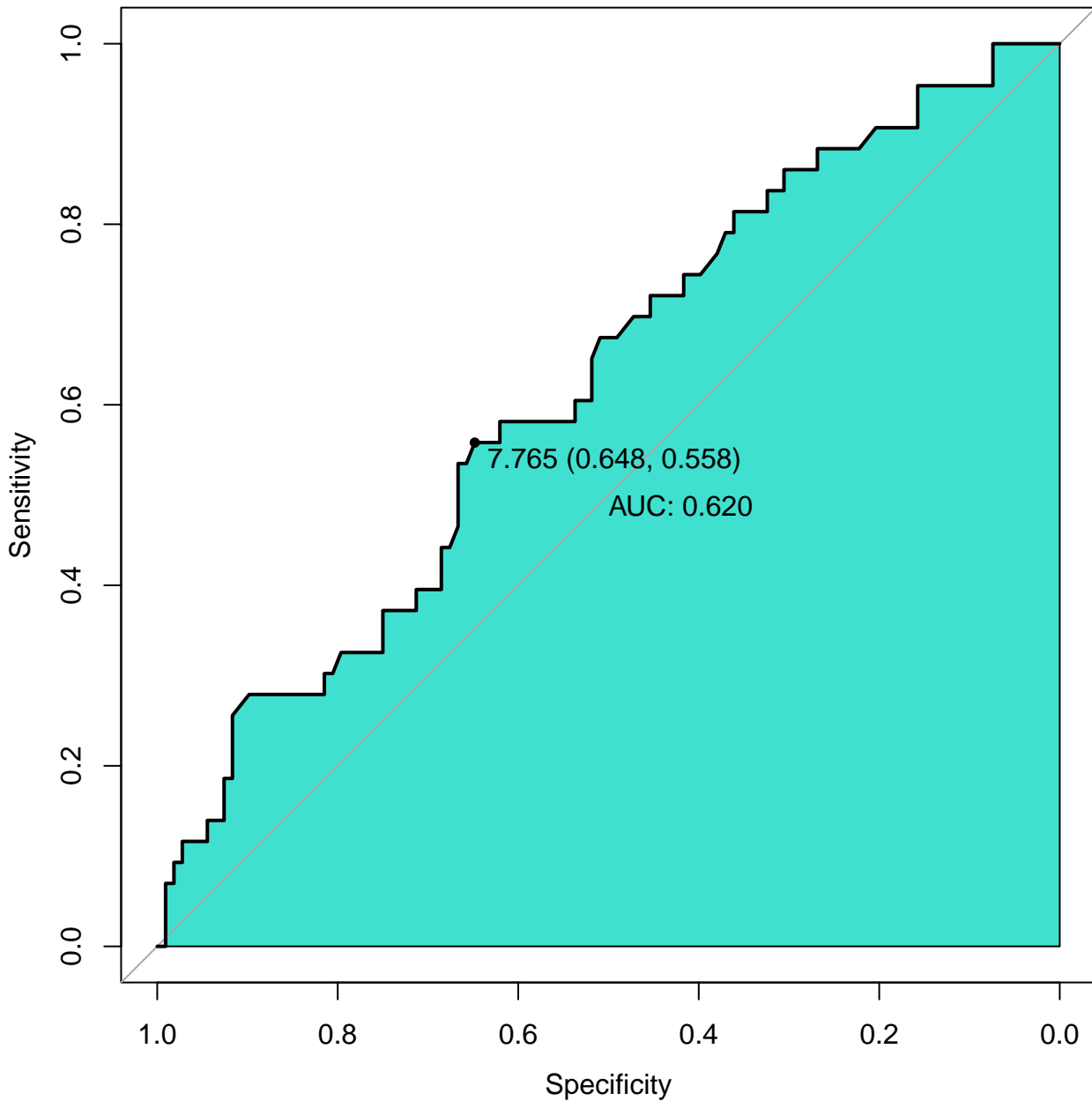

Supplement: Supplementary file 1 [file DataSheet1.zip › Supplement/ROC/m3_round_trip_walk_time.pdf]

## Histogram of age

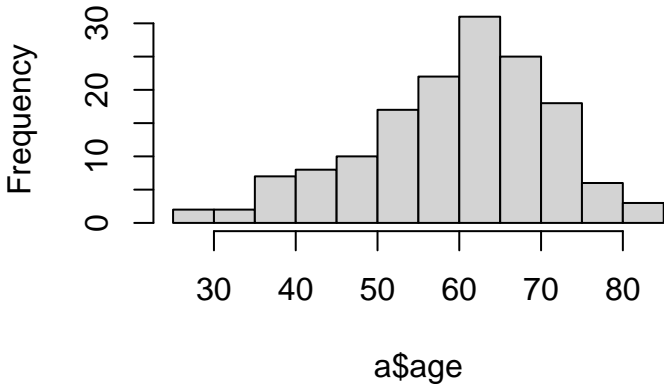

Supplement: Supplementary file 1 [file DataSheet1.zip › Supplement/age/age_hist.pdf]

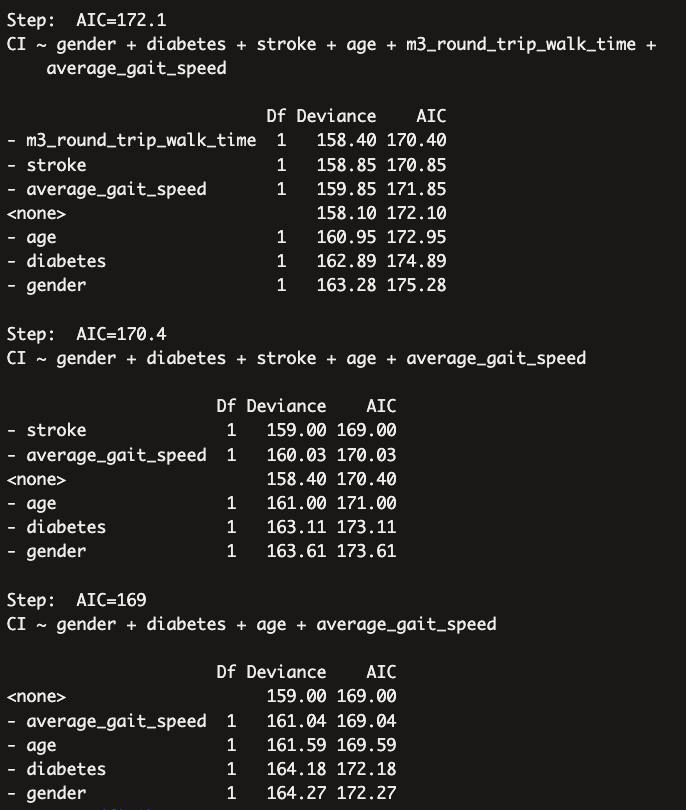

Supplement: Supplementary file 1 [file DataSheet1.zip › Supplement/stepwise/step2.png]

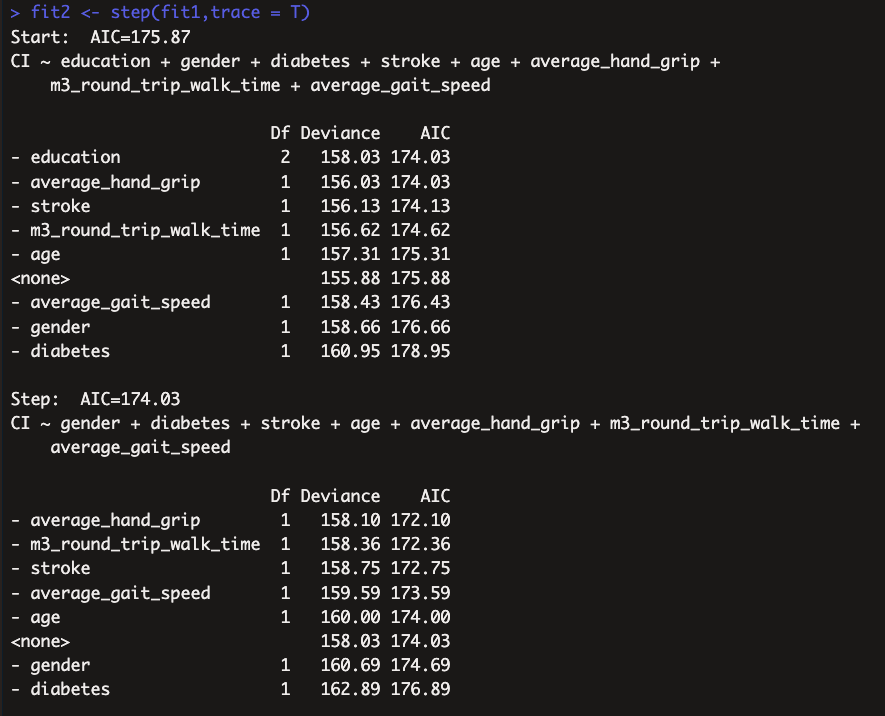

Supplement: Supplementary file 1 [file DataSheet1.zip › Supplement/stepwise/step1.png]
